# Prevalence and risk factors of adverse work outcomes among manufacturing workers with Long COVID in Malaysia – A nationwide study

**DOI:** 10.1371/journal.pone.0357125

**Published:** 2026-09-11

**Authors:** Hanizah Mohd Yusoff, Sheng Qian Yew, Azmawati Mohd Nawi, Noorlaili Mohd Tohit, Nor Ba’yah Abdul Kadir, Anita Abd Rahman, Rahmat Dapari, Ohnmar Htwe, Muhamad Hazizi Muhamad Hasani, Sivarajan Ramasamy, Zuraida Mohamad, Radzi Abdul Rashid, Mazrura Sahani, Azuraliza Abu Bakar, Ida Rosnita Ismail, Muhamad Ariff Muhamad Noordin, Fazlin Mohd, Amalia Sakina, Kamilah Amnani

**Affiliations:** 1 Department of Public Health Medicine, Faculty of Medicine, Universiti Kebangsaan Malaysia, Bandar Tun Razak, Kuala Lumpur, Malaysia; 2 Department of Family Medicine, Faculty of Medicine, Universiti Kebangsaan Malaysia, Bandar Tun Razak, Kuala Lumpur, Malaysia; 3 Center for Psychology and Human Well-Being, Faculty of Social Sciences and Humanitarian, Universiti Kebangsaan Malaysia, Bangi, Selangor, Malaysia; 4 Department of Community Health, Faculty of Medicine and Health Science, Universiti Putra Malaysia, UPM Serdang, Serdang, Selangor, Malaysia; 5 Faculty of Medicine, Universiti Sultan Zainal Abidin Medical, Kuala Terengganu, Terengganu, Malaysia; 6 Negeri Sembilan State Health Department, Seremban, Negeri Sembilan, Malaysia; 7 OSH ProCare Consultant and Services, G&C Township, Seremban, Negeri Sembilan, Malaysia; 8 Faculty of Health Sciences, Centre of Health and Applied Sciences, Universiti Kebangsaan Malaysia, Kuala Lumpur, Malaysia; 9 Faculty of Information Science and Technology, Center for Artificial Intelligence Technology, Universiti Kebangsaan Malaysia, UKM, Bangi, Selangor, Malaysia; 10 Graduate School of Business, Universiti Kebangsaan Malaysia, Bangi, Selangor, Malaysia; 11 Research Management Center, National Institute of Occupational Safety and Health, Bandar Baru Bangi, Selangor, Malaysia; 12 UKM Pakarunding Sdn Bhd, Level 3, Universiti Kebangsaan Malaysia, Bangi, Selangor, Malaysia; University of Nottingham Malaysia, MALAYSIA

## Abstract

**Introduction:**

The increasing prevalence and multiorgan involvement of Long COVID among the general population has raised concerns about the possible long-term effect of this condition among industrial workers. Therefore, this study seeks to determine the prevalence and risk factors of adverse work outcomes among Malaysian manufacturing workers affected by Long COVID.

**Materials and methods:**

A cross-sectional survey was conducted using the Long COVID Questionnaire, Work-Related Quality of Life (WRQoL) Scale, and the Work Productivity and Activity Impairment (WPAI) Questionnaire, which were physically distributed to previously-COVID-19 infected participants via convenient sampling. Simple logistic regression and multiple logistic regression were used to determine the association between the various risk factors and adverse work outcomes.

**Results:**

Among the 797 participants, a quarter (24.7%) of the workers with Long COVID demonstrated poor WRQoL. A total of 12.8% and 42.4% of workers with Long COVID had issues of sickness absenteeism and sickness presenteeism, respectively. Only 6.3% of the Long COVID workers had safety issues at work. Socio-clinical factors [i.e., older age (aOR=1.02) and smoking (aOR=1.38)], Long COVID symptoms [i.e., increased number of Long COVID symptoms (aOR=1.15)], work factors [i.e., engaged in shift work (aOR=1.25), handling of chemicals hazardous to health (aOR=2.51), exposure to confined space entry (aOR=1.60), exposure to excessive noise (aOR=1.55), and manual handling (aOR=1.33)], organisational factors [i.e., absence of sickness absence policy (aOR=2.77)] were significant risk factors of adverse work outcomes among workers with Long COVID. Conversely, personal factors [i.e., informing employers about the Long COVID symptom (aOR=0.32)] had a protective effect on adverse work outcomes.

**Conclusions:**

The impact of Long COVID on WRQoL, sickness absenteeism, sickness presenteeism, safety issues at work, as well as the multifactorial nature of adverse work outcomes highlights the need for targeted workplace interventions, supportive policies, early detection, and inclusive occupational health frameworks at the workplace.

## 1. Introduction

The manufacturing sector is a key contributor to Malaysia’s economy, accounting for the second-largest share of the country’s gross domestic product (GDP) and an annual contribution exceeding RM300 billion [1]. Furthermore, it provides employment for approximately 2.33 million individuals as of November 2022 [2]. The industry’s reliance on skilled labour is crucial for its efficient operation, making the retention of talented, skilled, and experienced workers essential for enhancing overall performance, quality, efficiency, effectiveness, and productivity within the sector [3]. Consequently, maintaining a healthy and productive workforce is vital. Unfortunately, the high number of COVID-19 clusters among manufacturing workers during the pandemic, along with the subsequent high prevalence of Long COVID, has raised concerns regarding the long-term sustainability of this sector [4].

The World Health Organization (WHO) defines Long COVID as a condition that develops in individuals with a prior SARS-CoV-2 infection, typically occurring three months after the initial onset of COVID-19 [5]. Additionally, the symptoms must persist for at least two months and cannot be attributed to any other underlying condition. Generally, Long COVID presents with a wide range of symptoms, which are thought to result from a pathology similar to that of acute COVID-19. Some of the most commonly reported symptoms include fatigue, shortness of breath, and cognitive impairment [6].

Given its reported impacts on multiple organ systems among the general population, Long COVID could possibly affect daily functioning, including workplace activities. For instance, workers experiencing Long COVID may face various negative work-related consequences, such as decreased productivity and performance, absenteeism, presenteeism, a higher risk of occupational injuries, and a reduced work-related quality of life [7,8]. Furthermore, workers requiring prolonged medical leave for recovery, ongoing treatment, or those unable to return to work may contribute to increased operational expenses for employers. It is also worth mentioning that the extent of these adverse work outcomes may vary depending on several factors, including the availability of workplace adjustment, company policies on sick leave, and return-to-work strategies implemented within the organisation.

While previous research has documented various negative effects of Long COVID, most studies have primarily focused on the general population [9,10]. Consequently, there is limited knowledge regarding the specific impact of Long COVID on work-related outcomes among manufacturing workers. An updated study on the burden of Long COVID within this sector would help workers, employers, policymakers, and other stakeholders better understand the scale of the issue affecting the manufacturing industry. On the basis of these research gaps and this rationale, this study aims to assess the prevalence and risk factors associated with adverse work outcomes among manufacturing workers in Malaysia who are affected by Long COVID.

## 2. Materials and methods

A cross-sectional survey was conducted from April 2024 to October 2024 across Malaysia’s five geographical zones (i.e., East Coast, Northern, Southern, Central, and East Malaysia). The target population consisted of workers from manufacturing companies listed in the Federation of Malaysian Manufacturers (FMM) directory, with eligibility criteria aligned with those specified in Table 1. Initially, a multistage sampling strategy was planned for the selection of manufacturing companies, involving cluster sampling within each geographical zone based on the total number of companies, followed by proportionate stratified sampling to select workers from the states within each zone. However, due to a very low response rate (e.g., companies declining participation), the researchers opted to use a convenience sampling method instead. Under this approach, any company that provided consent to participate from each zone in Malaysia was included. This resulted in three, four, three, six, and two companies from the East Coast, Northern, Southern, Central, and East Malaysia respectively participating. This was followed by a universal sampling to select workers from each of these companies. Given a precision level of 0.05, a confidence level of 95%, a prevalence of adverse work outcomes of 50.0% (in the absence of data on prevalence of adverse work outcome among workers with Long COVID), and a non-response rate of 50%, the total sample size required nationwide was 768. This sample size estimation was calculated using Kish’s formula [11].

**Table 1:** Inclusion and exclusion criteria for manufacturing workers.

Inclusion criteria	Exclusion criteria
1. Workers of manufacturing company (including electrical, electronics, petroleum, chemical, rubber, plastic, non-metallic mineral products, basic metal, fabricated metal, food and beverages, tobacco, wood, furniture, paper products, printing, textile, wearing apparel, leather, footwear, and transport equipment sectors). 2. Workers diagnosed with COVID-19 between 1^st^ January 2022 and 30^th^ April 2024. 3. Malaysian and legal foreign workers. 4. Worked for at least six months prior to the acute phase of COVID-19. 5. Able to communicate in English and/or Malay.	1. Workers who had resigned or terminated. 2. Temporary workers. 3. Pregnant women during data collection or during acute phase of COVID-19. 4. Workers who did not consent for participation.

Three previously validated questionnaires, namely the Long COVID Questionnaire, Work-Related Quality of Life (WRQoL) Scale [12], and the Work Productivity and Activity Impairment (WPAI) Questionnaire [13], were utilised to assess the prevalence and risk factors of adverse work outcomes. Permission to use these questionnaires was obtained from their respective developers.

The 67-item Long COVID Questionnaire was used to evaluate both the presence of Long COVID symptoms and the associated adverse work outcomes among manufacturing workers [14]. The questionnaire is divided into three sections. Section A comprises seven items designed to collect socio-clinical data, including age, gender, BMI (from self-reported weight and height), comorbidities, and smoking status. Section B contains 57 items assessing the presence of Long COVID, where a worker is classified as having Long COVID if they respond with “Yes” for part a and b and “No” for part c of the symptoms listed. Lastly, Section C consists of three items aimed at identifying workplace safety concerns. A worker is considered to have safety issues at work if they select “unsure,” “affected,” or “very much affected” for at least one item in this section.

The 24-item WRQoL Scale was used to assess the perceived quality of life of the manufacturing workers with Long COVID [12]. WRQoL was measured through six psychosocial subscales (i.e., general well-being, home-work interface, job-career satisfaction, control at work, working conditions, and stress at work). For score interpretation, items 7, 9, and 19 are reverse-coded. In this study, the total WRQoL score was derived by summing all 24 item scores, with a score of 83 or higher indicating a high quality of work life, while a score below 83 is classified as low WRQoL.

**Fig 1:**
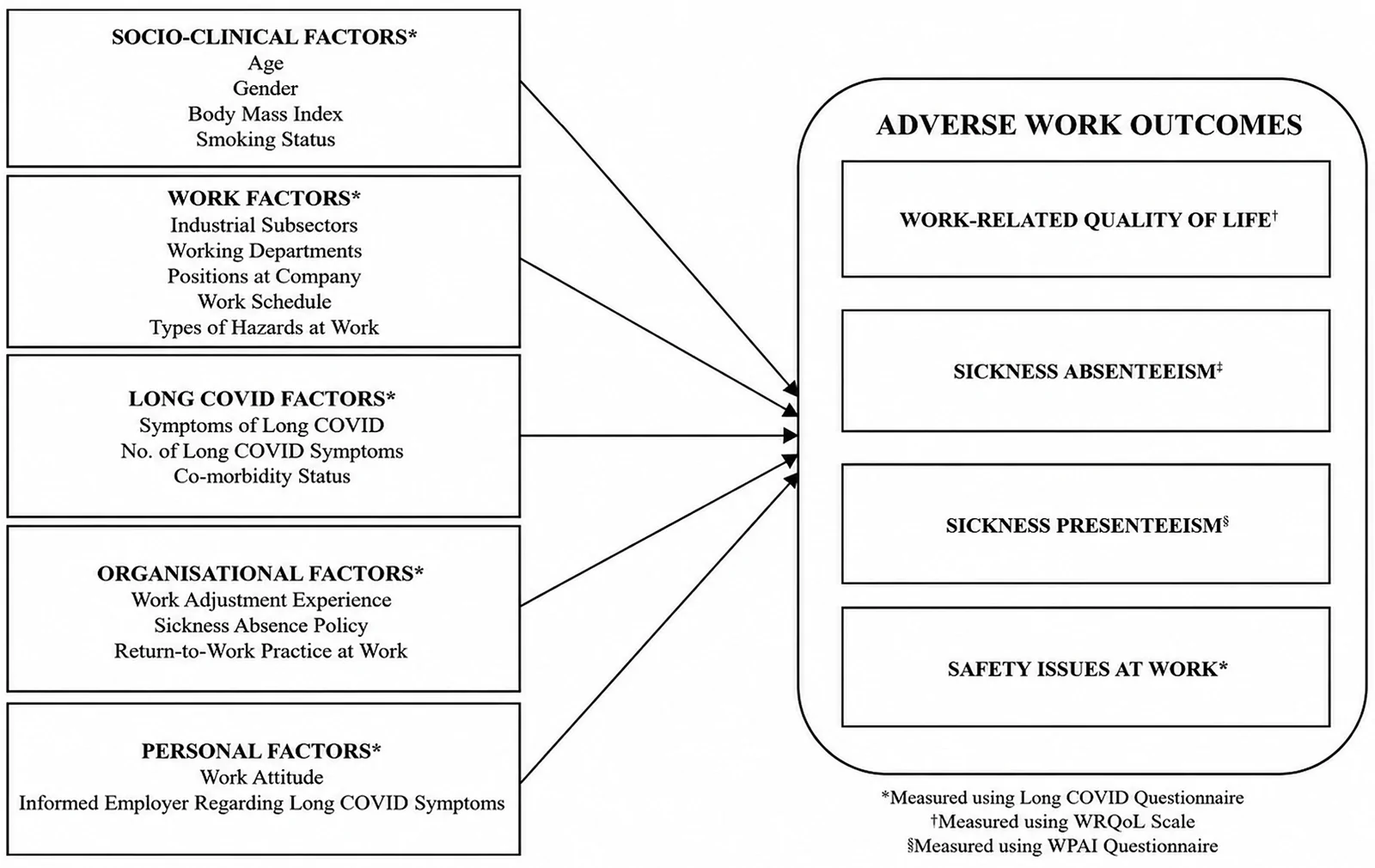
The conceptual framework of the study.

The 6-item, unidimensional WPAI Questionnaire was employed to evaluate sickness absenteeism and presenteeism among manufacturing workers affected by Long COVID [13]. A worker is classified as having sickness absenteeism if they missed at least 1% of their assigned work time in the past seven days. Likewise, sickness presenteeism is identified when a worker reports at least a 1% reduction in work performance due to health issues within the same period. The conceptual framework of the study is shown in Fig 1.

Data collection involved on-site visits by 2–5 researchers and/or enumerators at each selected manufacturing company. In total, there were 32 researchers and/or enumerators, who were trained using the standardised data collection protocol. This allowed the study to be conducted nationwide over the same period using a standardised protocol. After obtaining informed consent from the company, the human resource personnel were contacted to provide a list of workers (i.e., the sampling frame) who had been diagnosed with and documented as having acute COVID-19. With the cooperation of the management, the selected workers were assembled in a designated room within the management office. Each worker was then screened for eligibility based on the criteria outlined in Table 1. Eligible workers were provided with a briefing on the study objectives, instructions for questionnaire administration, and their rights before providing informed consent to participate. Those who were deemed ineligible or who declined participation were thanked and permitted to return to their work. Prior to commencing the survey, informed consent was obtained from all eligible workers. They were encouraged to complete the questionnaire with the assistance of the researchers and/or enumerators, and sufficient time was allocated to ensure high-quality data collection. The role of the researcher and/or enumerators was to clarify items and explain complex medical terminology in the questionnaire. Workers’ anonymity and confidentiality were upheld throughout the study. To minimise disruptions to workplace operations, data collection was conducted in a staggered manner, with only one to two workers completing the questionnaire at a time.

Descriptive analysis was conducted to present the prevalence of adverse work outcomes and risk factors of adverse work outcomes. Simple logistic regression was performed to examine the association between the various risk factors (including socio-clinical, Long COVID symptoms, work, organisational, and personal factors) and adverse work outcomes. Risk factors with a p-value < 0.25 were included in a multiple logistic regression model. Additionally, the moderating effects of organisational and personal factors on Long COVID were assessed using multiple logistic regression. All statistical analyses were performed using SPSS version 28.

Ethical approval for this study was obtained from the Research Ethics Committee of the National University of Malaysia (JEP-2023–607) and the Medical Research and Ethics Committee (MREC) of the Ministry of Health Malaysia (NMRR ID-23–03310-H3E). Ethical approval was obtained from the Medical Research and Ethics Committee (MREC) as the study included healthcare professionals from the Ministry of Health Malaysia. Participants recruitment began on 15^th^ January 2024 and ended on 16^th^ December 2024. All participants provided written informed consent prior to their participation. In addition, written or electronic consent was secured from the participating manufacturing companies prior to data collection.

## 3. Results

In the present study, adverse work outcomes included poor WRQoL, work productivity (sickness absenteeism and sickness presenteeism), and safety issues at work (Table 2). In terms of the prevalence of adverse work outcomes, almost a quarter of the workers with Long COVID (24.7%) exhibited poor WRQoL. Sickness absenteeism and sickness presenteeism were reported by 12.8% and 42.4% of workers with Long COVID, respectively. Only a small number of workers (6.3%) reported experiencing safety issues at work.

**Table 2:** Prevalence of adverse work outcomes among the manufacturing workers with Long COVID.

Adverse work outcomes	Workers with LC, n (%) N = 797
**Work-related quality of life (WRQoL)**	
Poor	197 (24.7)
Good	600 (75.3)
**Work productivity (sickness absenteeism)**	
Yes	102 (12.8)
No	695 (87.2)
**Work productivity (sickness presenteeism)**	
Yes	338 (42.4)
No	459 (57.6)
**Safety issues at work**	
Yes	50 (6.3)
No	747 (93.7)

The risk factors of adverse work outcomes investigated in the present study include socio-clinical, Long COVID symptoms, work, organisational, and personal factors. The socio-clinical factors of the manufacturing workers with Long COVID were presented in Table 3. The mean age of the workers was 37.4 ± 9.5 years. Most workers were female (54.5%), overweight (37.6%) and did not have comorbidities (64.4%). Among those with co-morbidities, hypertension (13.2%) was the most common, followed by asthma (7.8%) and diabetes (5.4%). Additionally, the majority of workers were non-smokers (78.2%).

**Table 3:** Socio-clinical factors of the manufacturing workers with Long COVID.

Individual factors among manufacturing workers	Mean (SD)	n (%) N = 797
**Age (years)**	37.4 (9.5)	–
**Gender**		
Male	–	363 (45.5)
Female	–	434 (54.5)
**BMI category (kg/m**^**2**^)		
< 18.5 (underweight)	–	38 (4.8)
18.5–24.9 (normal)	–	276 (34.6)
25.0–29.9 (overweight)	–	300 (37.6)
> 30 (obese)	–	183 (23.0)
**Comorbidities**		
Total comorbidities (may overlap)	–	284 (35.6)
Hypertension	–	105 (13.2)
Asthma	–	62 (7.8)
Diabetes	–	43 (5.4)
Anxiety/Panic attack	–	16 (2.0)
Heart disease	–	11 (1.4)
Autoimmune disease	–	10 (1.3)
Chronic kidney disease	–	4 (0.5)
Chronic liver disease	–	1 (0.1)
Cancer	–	0 (0.0)
HIV Infection	–	0 (0.0)
Others^┼^	–	94 (11.8)
**Smoking status**		
Non-smokers	–	623 (78.2)
Smokers	–	174 (21.8)

*Range of age = 22–63 years old.

^┼^Others include gastritis, dyslipidaemia, gout, and psoriasis.

As presented in Table 4, among the 797 manufacturing workers with Long COVID, the most common symptoms for Long COVID were post-exertional malaise (50.9%), followed by fatigue (37.4%), and joint pain (25.6%). The number of Long COVID symptoms experienced per worker was 3.2 ± 2.8.

**Table 4:** Long COVID symptoms of the manufacturing workers.

Long COVID symptoms	n (%) N = 797	Mean (SD)
Post-exertional malaise	406 (50.9)	
Fatigue	298 (37.4)	
Joint pain	204 (25.6)	
Muscle pain	171 (21.5)	
Cough	164 (20.6)	
Headache	142 (17.8)	
Tingling	140 (17.6)	
Cognitive dysfunction	136 (17.1)	
Dyspnoea	133 (16.7)	
Tachycardia	133 (16.7)	
Chest Pain	120 (15.1)	
Sleeping disturbance	105 (13.2)	
Intermittent fever	93 (11.7)	
Dizziness	91 (11.4)	
Tinnitus	77 (9.7)	
Diarrhoea	55 (6.9)	
Altered sensation	39 (4.9)	
Abdominal pain	27 (3.4)	
Depression	26 (3.3)	
**No. of Long COVID Symptom(s)**		3.2 (2.8)

Table 5 presents the work factors of the manufacturing workers with Long COVID. Most of the workers interviewed were working in the electrical and electronics subsectors (52.3%) and most of them were working in the operation/supply chain department (84.8%)”. Additionally, most of the workers were operators (40.8%) and primarily worked normal office hours (51.9%). In terms of types of hazards at work, the most commonly identified workplace hazards included visual display units (VDU) (61.9%), manual handling (40.8%), and excessive noise (29.5%).

**Table 5:** Description of work factors of the manufacturing workers with Long COVID.

Work factors of the manufacturing workers	n (%) N = 797
**Industrial subsectors**	
Electrical and electronics	417 (52.3)
Transport, vehicle and equipment	163 (20.5)
Rubber	119 (14.9)
Textile, wearing apparel and leather	74 (9.3)
Chemical (including petroleum)	11 (1.4)
Paper, printing, and publishing	10 (1.3)
Food, beverage, and tobacco	3 (0.4)
**Working departments**	
Operations/supply chain	676 (84.8)
Research and development (R&D)	29 (3.6)
Human resource	23 (2.9)
Finance (including accounts)	20 (2.5)
Facilities	17 (2.1)
Sales	8 (1.0)
Procurement	8 (1.0)
Marketing	5 (0.6)
Corporate	3 (0.4)
Others	8 (1.0)
**Positions at company**	
Operator	325 (40.8)
Technician, specialist or permit holder	199 (25.0)
Executive	77 (9.7)
Supervisor	74 (9.3)
Officer	47 (5.9)
Manager	46 (5.8)
Clerk	15 (1.9)
General worker	5 (0.6)
Support services	4 (0.5)
Director	1 (0.1)
Others	4 (0.5)
**Work schedule**	
Normal/office hours	414 (51.9)
Shift including staggered shift	382 (47.9)
Flexible	1 (0.1)
Work from home	0 (0.0)
**Types of hazards at work**	
VDU (visual display unit)/video (computer)	493 (61.9)
Manual handling	325 (40.8)
Excessive noise	235 (29.5)
Handling of hazardous chemicals	206 (25.8)
Electrical work	158 (19.8)
Working in extreme temperatures (hot or cold)	99 (12.4)
Confined space entry	91 (11.4)
Working at heights	60 (7.5)
Lone/isolated work	45 (5.6)
Driving vehicle	35 (4.4)
Working on roofs	33 (4.1)
Construction work	30 (3.8)
Driving lift truck	28 (3.5)
^┼^Others	40 (5.0)

^┼^Example: Working with sharp objects and risk of falling objects.

Analysis of the organisational factors found that 53.3% of the workers with Long COVID perceived that work adjustment was feasible at their workplaces (Table 6). Furthermore, almost half (49.4%) of the workers with Long COVID perceived that a sickness absence policy was present at their workplace. Only 32.9% of the workers believed that return-to-work (RTW) practices were present at their workplace.

**Table 6:** Description of organisational factors of manufacturing workers with Long COVID.

Organisational factors of manufacturing workers with Long COVID	n (%) N = 797	
**Yes (%)**	**No (%)**
Presence of work adjustment feasibility	425 (53.3)	372 (46.7)
Presence of sickness absence policy at workplace	394 (49.4)	403 (50.6)
Presence of RTW practices at workplace	262 (32.9)	535 (67.1)

In terms of personal factors, most of the workers with Long COVID had a good work attitude (93.9%), despite their condition (Table 7). Among the workers with Long COVID, most did not inform their employers (89.1%).

**Table 7:** Description of personal factors of manufacturing workers with Long COVID = .

Personal factors of manufacturing workers with Long COVID	Workers with LC, n (%) N = 797
**Work attitude**	
Good	748 (93.9)
Poor	49 (6.1)
**Employer informed regarding Long COVID**	
Yes	87 (10.9)
No	710 (89.1)

To investigate the association with WRQoL, a total of 21 risk factors (i.e., socio-clinical, Long COVID symptoms, work, organisational, and personal factors with p < 0.25 in the simple logistic regression) were modelled using multiple logistic regression (MLR) with a forward method (S1 Table). All assumptions of MLR (i.e., linearity, interaction, multicollinearity, and outliers) were met. Out of the 21 risk factors, only five risk factors were significantly associated with WRQoL, namely age, smoking status, excessive noise, confined space entry and sickness absence policy at workplace. For workers with Long COVID, each additional year of age is associated with a 2% increase in the odds of experiencing poor WRQOL (aOR = 1.02, p < 0.001). Workers with Long COVID who smoke have 1.38 times higher odds of suffering from poor WRQOL (aOR = 1.38, p < 0.001), compared with those who did not smoke. Workers with Long COVID who were exposed to excessive noise had 1.55 times higher odds of suffering from poor WRQOL (aOR = 1.55, p < 0.001), compared with those who were not exposed. Workers with Long COVID subjected to confined space entry had 1.60 times higher odds of suffering from poor WRQOL (aOR = 1.60, p = 0.013), compared with those who were not exposed. Workers who have experienced the absence of a sickness absence policy at the workplace had 2.77 times higher odds of suffering from poor WRQOL (aOR = 2.77, p < 0.001), compared with those who had a sickness absence policy.

To investigate the association with sickness absenteeism, a total of 10 risk factors (i.e., socio-clinical, Long COVID symptoms, work, organisational, and personal factors with p < 0.25 in the simple logistic regression) were modelled using MLR with a forward method (S2 Table). All assumptions of MLR (i.e., linearity, interaction, multicollinearity, and outliers) were met. Out of the 10 risk factors, only manual handling was significantly associated with sickness absenteeism. Workers with Long COVID who are exposed to manual handling had 1.33 times higher odds of having sickness absenteeism (aOR = 1.33, p = 0.037), compared with those who were not exposed.

To investigate the association with sickness presenteeism, a total of 17 risk factors (i.e., socio-clinical, Long COVID symptoms, work, organisational, and personal factors with p < 0.25 in the simple logistic regression) were modelled using MLR with a forward method (S3 Table). All assumptions of MLR (i.e., linearity, interaction, multicollinearity, and outliers) were met. Out of the 17 risk factors, only three risk factors were significantly associated with sickness presenteeism, namely work schedule, number of Long COVID symptoms, and employers informed regarding the Long COVID symptoms. Workers with Long COVID who engaged in shift work had 1.25 times higher odds of having sickness presenteeism (aOR = 1.25, p = 0.027), compared with those who engaged in normal working hours. The odds of having sickness presenteeism increased by 1.15 times for every increase in the number of Long COVID symptoms (aOR = 1.15, p < 0.001). Informing the employer regarding the Long COVID has been found to be a protective factor against sickness presenteeism where they had 42% lower odds of having sickness presenteeism (aOR = 0.58, p = 0.019), compared with those who did not inform.

To investigate the association with safety issues at work, a total of 26 risk factors (i.e., socio-clinical, Long COVID symptoms, work, organisational, and personal factors with p < 0.25 in the simple logistic regression) were modelled using MLR with a forward method (S4 Table). All assumptions of MLR (i.e., linearity, interaction, multicollinearity, and outliers) were met. Out of the 26 risk factors, only two risk factors were significantly associated with safety at work, namely handling of chemicals hazardous to health and employers informed regarding Long COVID symptoms. Workers with Long COVID who handled chemicals hazardous to health had 2.51 times higher odds of having safety issues at work (aOR = 2.51, p < 0.001), compared with those who did not handle. Employer informed regarding their Long COVID has been found to be a protective factor against safety issues at work where they had 68% lower odds of having safety issues at work (aOR = 0.32, p = 0.007), compared with those who did not inform.

According to the multiple logistic regression, it was found that none of the organisational factors and personal factors had a moderating effect on the relationship between Long COVID symptoms and adverse work outcomes (S5 Table).

## 4. Discussion

Our findings suggest that a relatively low prevalence (less than a quarter) of workers with Long COVID had poor WRQoL. In fact, a recent study also found that there were no significant differences in the QoL between those with and without Long COVID [15]. With regard to the seemingly paradoxical finding of relatively high WRQoL despite the presence of Long COVID, several explanations are possible. Firstly, sufficient coping strategies among workers may mitigate the perceived impact of persistent symptoms on WRQoL. For instance, work-focused rehabilitation, multidisciplinary inpatient and outpatient rehabilitation, psychoeducation, pacing, and breathing strategies, have been shown to shift focus from symptom monitoring to optimising functional outcomes. These strategies allowed workers affected by Long COVID to maintain good WRQoL. Secondly, many participants may have experienced mild or manageable symptoms (as most participants experienced only one symptom) that did not substantially impair their work functioning, as observed in the low proportion of workers with sickness absenteeism, sickness presenteeism, and safety issues at work. Hence, it can be concluded that although Long COVID could affect multiple body systems, these symptoms are manageable and have little impact on WRQoL.

It is noteworthy that although only 12.8% of the workers with Long COVID absent from their work due to the symptoms, a relatively large proportion of workers (42.4%) continued to work despite having Long COVID. This may lead to reduced productivity, prolonged recovery times, and potential worsening of symptoms due to the lack of rest and proper medical care [16]. This highlights the importance of workplace accommodations, such as flexible work hours, remote work options, or lighter workloads, to support employees dealing with persistent symptoms and help them achieve a full recovery while maintaining their employment.

Workers with Long COVID who are older, smokers, exposed to excessive noise, working in confined spaces, and without a sickness absence policy face higher odds of experiencing poor WRQoL due to several factors. Older workers often have weakened immune systems and slower recovery times [17], which can exacerbate fatigue and mobility issues, leading to reduced work capacity. Smoking worsens respiratory symptoms, reduces lung function, and slows the healing process [18], making recovery from Long COVID more challenging. Exposure to excessive noise can contribute to fatigue, stress, headaches, and sleep disturbances [19], all of which may amplify Long COVID symptoms. Additionally, working in confined spaces with poor ventilation can worsen Long COVID symptoms, especially within the respiratory system [20]. Lastly, the absence of a sickness absence policy adds to the problem by preventing proper rest and recovery [21], as workers may feel pressured to continue working despite their symptoms, leading to stress and anxiety. All these factors can create a cycle of deteriorating health and diminished WRQoL. As such, there is an urgent need for supportive workplace policies and accommodations to help mitigate risks and support recovery.

Workers with Long COVID who are exposed to manual handling have higher odds of sickness absenteeism due to the physical and physiological demands of their work. Chronic fatigue and muscle weakness, common in Long COVID, can make the physical exertion required for tasks like lifting, pushing, or pulling heavy objects difficult to sustain. Persistent joint and muscle pain can be worsened by manual handling [22], leading to the need for time off to recover. Respiratory symptoms, such as dyspnoea, further reduces stamina and makes manual tasks particularly challenging [23]. Additionally, post-exertional malaise, where symptoms worsen after physical activity [24], can prolong recovery and contribute to more frequent sickness absences. These factors highlight the need for workplace accommodations, such as task modifications or lighter duties, to help workers manage their symptoms and maintain their employment.

Workers with Long COVID who engage in shift work and experience a high number of symptoms are more likely to face sickness presenteeism. Shift work disrupts normal sleep patterns and can lead to chronic fatigue [25], which compounds Long COVID symptoms such as exhaustion, brain fog, and muscle pain. The irregular hours can also interfere with the workers’ ability to recover, leading to persistent or worsened symptoms. However, many workers may continue to attend work despite their symptoms due to fear of job loss, financial insecurity, or a workplace culture that discourages absences. In addition, the high number of Long COVID symptoms further exacerbates sickness presenteeism, as these workers may be physically and/or mentally unfit to perform their duties effectively but continue working out of necessity or obligation [26]. On the other hand, employer informed regarding the Long COVID was a protective factor against sickness presenteeism. When employers are aware of an employee’s health condition, they are more likely to implement supportive measures such as flexible work arrangements, reduced workload, or modified duties tailored to the employee’s physical and mental limitations [27]. These accommodations can help reduce the pressure to attend work while unwell and allow for proper symptom management and recovery. Additionally, open communication can reduce fear of job loss or stigma associated with taking sick leave, creating a more supportive work environment.

Workers with Long COVID who handle hazardous chemicals may face higher odds of safety issues at work because fatigue and malaise can impair their ability to respond quickly to chemical hazards or emergencies [28]. Additionally, cognitive impairments such as brain fog, poor memory, and difficulty concentrating increase the likelihood of errors in handling hazardous chemicals [29]. On the other hand, workers with Long COVID who inform their employers about their symptoms may experience fewer safety issues. This is because informing employers allows workers to receive reasonable accommodations [30], such as adjusted workloads, flexible schedules, or reduced exposure to hazardous chemicals, which can help mitigate safety risks.

### 4.1. Strengths and limitations of the study

This study has several key strengths. Firstly, its nationwide coverage, which includes manufacturing workers from all states in Malaysia, enhances the generalisability of its findings. Secondly, to the best of the researchers’ knowledge, this is the first study to determine the prevalence and risk factors of adverse work outcomes among manufacturing workers, offering valuable insights for occupational health policies and interventions. To fully account for potential confounding symptoms or pre-existing conditions that could overlap with or mimic Long COVID, such as bronchial asthma and chronic obstructive pulmonary disease, several measures were incorporated into the study design and data collection process. Firstly, information on pre-existing comorbidities (e.g., respiratory, cardiovascular, and metabolic conditions) was collected and participants were included in the multivariate analysis to allow adjustment for potential confounders. Secondly, participants were required to have a confirmed diagnosis of COVID-19 within a defined time frame (i.e., fully fulfilling the WHO definition), to ensure temporal relevance to Long COVID assessment. Thirdly, symptoms were assessed in relation to their onset following COVID-19 infection, which helped differentiate new or persistent symptoms from pre-existing conditions.

Although the study has notable strengths, it also has some limitations. A major limitation is its cross-sectional design, which prevents the establishment of causal relationships between risk factors and adverse work outcomes. Secondly, the use of convenience sampling may have introduced selection bias, as participants who were more willing or available to participate could differ systematically from those who did not participate. Consequently, the findings may not be fully generalisable to all Malaysian manufacturing workers with Long COVID. Thirdly, the study relied on self-reported information regarding previous COVID-19 infection, Long COVID symptoms, workplace exposures, and adverse work outcomes, which may be subject to recall bias. Participants may have underreported or overreported their symptoms and occupational experiences, particularly for events occurring several months prior to the survey. In addition, there is a possibility of misclassification bias, particularly in the identification of Long COVID status and workplace exposures, as no objective clinical verification or workplace exposure measurements were performed. Some participants may have inaccurately classified their symptoms or exposures, potentially affecting the observed associations. Nevertheless, the study provides important preliminary evidence on the occupational impacts of Long COVID among manufacturing workers and highlights the need for further prospective studies using probability sampling methods and objective assessments.

### 4.2. Recommendations for future studies

Future research should consider using a longitudinal study design to determine causal relationships between risk factors and adverse work outcomes. Additionally, integrating objective clinical assessments alongside self-reported data could help reduce recall bias and improve the accuracy of symptom reporting. This may include medical evaluations, workplace health monitoring, or biomarkers for Long COVID. To improve generalisability, future studies should expand beyond the manufacturing sector to include workers from various industries with different occupational exposures. This would provide a broader understanding of Long COVID’s impact across work environments and support the development of industry-specific recommendations.

### 4.3. Study implications

The findings of this study have important implications for workplace interventions and occupational health policies. Firstly, identifying key risk factors for adverse work outcomes underscores the need for targeted workplace interventions. Employers should implement preventive measures such as enhancing workplace ventilation, minimising exposure to hazardous chemicals, and addressing ergonomic risks associated with prolonged VDU use. Additionally, the study highlights the importance of early detection and management of Long COVID among workers, which could be facilitated through regular health screenings and medical support programmes. Moreover, the findings suggest that the lack of a structured sickness absence policy may contribute to negative work outcomes. Employers should establish clear guidelines on sickness absence and return-to-work strategies to support employees recovering from Long COVID. Furthermore, the study indicates that disclosing Long COVID symptoms to employers may have a protective effect, emphasising the importance of fostering a supportive and open work environment. This calls for awareness campaigns and training programmes to educate both employers and employees on the impact of Long COVID and the benefits of workplace adjustments.

The study also has wider implications for organisational health policies and regulations. Policymakers should incorporate Long COVID management into existing occupational health and safety frameworks to ensure adequate support for affected workers. Furthermore, based on the study’s identification of specific risk factors, future workplace guidelines could include sector-specific recommendations to help reduce the impact of Long COVID.

## 5. Conclusions

Three-quarters of the manufacturing workers with Long COVID demonstrated high WRQoL despite their illness. Approximately four in 10 workers with Long COVID had issues of sickness presenteeism while about 1 in 10 workers with Long COVID workers had issues of sickness absenteeism. A very small number of workers with Long COVID had safety issues at work. Individual factors (i.e., older age, female gender, and smoking), Long COVID symptoms (i.e., increased number of Long COVID symptoms), work factor (i.e., engaged in shift work, handling hazardous chemicals, exposure to confined space entry, and exposure to excessive noise), organisational factor (i.e., absence of sickness absence policy) were associated with adverse work outcomes among workers with Long COVID. Meanwhile, personal factors such as informing employers about Long COVID seem to have a protective effect on adverse work outcomes among the workers with Long COVID symptoms.

## Supporting information

S1 Table Association between various risk factors and poor work-related quality of life among manufacturing workers with Long COVID. (DOCX)

S2 Table Association between various risk factors and sickness absenteeism among manufacturing workers with Long COVID. (DOCX)

S3 Table Association between various risk factors and sickness presenteeism among manufacturing workers with Long COVID. (DOCX)

S4 Table Association between various risk factors and safety issues at work among manufacturing workers with Long COVID. (DOCX)

S5 Table Moderating effects in the association between Long COVID and adverse work outcomes. (DOCX)
