# Supplementary material for: Prevalence and risk factors of adverse work outcomes among manufacturing workers with Long COVID in Malaysia – A nationwide study
Source: PLoS One. 2026 Sep 11;21(9):e0357125. doi: 10.1371/journal.pone.0357125 (PMC13567834; doi:10.1371/journal.pone.0357125)
Supplement: Supplementary file 1 [file pone.0357125.s006.pdf]

Manuscript title : Prevalence and risk factors of adverse work outcomes among manufacturing workers with Long COVID in Malaysia – a nationwide study

Comments/suggestions to authors for further improvement:

No.	Section in manuscript	Comments/suggestions
1.	Title	▪ The title is clear and correctly reflects the key components of the study.
2.	Abstract	Introduction:<br>▪ The two sentences seem to contradict each other. The first statement mentions a high prevalence of Long COVID, while the second indicates that the study's goal was to determine the prevalence. Consider rephrasing.<br>▪ Consider highlighting the knowledge gap and problem statement in this section.<br><br>Materials and methods<br>▪ The instruments used are mentioned but the methods used to distribute the instruments are not. Please state accordingly.<br>▪ Consider moving the total number of respondents (i.e., 797) to the results section.<br>▪ Consider adding the inclusion and exclusion criteria of participants.<br>▪ Line 52 – to correct the spelling of the word 'utilized'.<br><br>Results<br>▪ For better clarity, consider to restructure the first part of the results section. I would suggest starting with the number of respondents, followed by prevalence and so on.<br>▪ Line 59-60: There is a repetition of information i.e., 42.4%. Consider rephrasing.<br>▪ Regarding the reported risk factors, consider reporting key effect sizes (e.g., adjusted odds ratios) with 95% confidence intervals to allow readers to interpret the magnitude of the associations.<br><br>Conclusion<br>The conclusion mostly repeats the results and is quite general. Consider making it stronger by emphasizing the main implications of the findings for occupational health practices or workplace policies that support workers dealing with Long COVID. This would give readers a clearer idea of the importance of the study.
3.	Introduction	Overall, this section is very well written, clearly outlining key definitions and emphasizing the problem statement and research gap.
4.	Methods	Study design<br>▪ It is clearly stated. I would recommend using the phrase 'cross-sectional survey' instead.<br><br>Sampling and population<br>▪ Sampling methods are clearly stated.<br>▪ Sample size and its calculation method is clearly stated.<br>▪ The inclusion and exclusion criteria of participants are clear.

No.	Section in manuscript	Comments/suggestions
4.	Methods (cont)	Instruments<br>▪ The instruments are well-described i.e., sections, score interpretations etc.<br>▪ Permission to use the instruments is clearly stated.<br><br>Data collection protocol<br>▪ The protocol was described but could be further elaborated.<br>▪ Consider including these information for better clarity:<br>i. Any method used to select the manufacturing companies<br>ii. The individual that the researcher reached out to provide the list.<br>iii. Were similar data collection protocols used at all companies visited.<br>iv. The number of researchers present at the site (if relevant).<br>v. The kind of assistance provided during the data collection e.g., explanation on medical terms such as tachycardia, tinnitus etc.<br>▪ Consider incorporating the specific types of demographic data gathered in this section. Furthermore, it would be beneficial to indicate the methods used for data collection, such as whether body weight and height were self-reported or measured.<br><br>Ethical approval<br>▪ It is unclear why the study requires MREC approval. To clarify.<br>▪ Consider indicating if any authorization was provided from the manufacturing companies to visit and conduct data collection.
5.	Results	Background data<br>▪ Consider presenting the geographic locations of the companies involved in this study. This would help readers better assess the representativeness of the sample and the generalizability of the findings to Malaysian manufacturing companies.<br>▪ Although it is included in Table 2 (currently referred to as Table 1), I suggest that the socio-clinical data of the participants be presented separately.<br><br>Prevalence of adverse work outcomes<br>▪ The prevalence is not explicitly mentioned. It remains uncertain whether the WRQoL results reflect the prevalence of negative work outcomes. In the subsequent sections (Discussion and Conclusion), different narratives were provided i.e., poor vs high WRQoL. Consider rephrasing.<br><br>Risk factors<br>▪ In my opinion, Table 3-7 solely present the data in percentage rather than factors that are determined by statistical analysis. I would strongly suggest renaming the table titles to prevent any potential misunderstanding among readers.<br>▪ Consider indicating the type of statistical analysis used, as footnote, in all tables provided as Appendix 1-4.
6.	Discussion	Overall, the extent and depth of the discussion is appropriate. All main results are discussed accordingly.
7.	Conclusion	The conclusion was drawn from main findings of the study and is appropriate.

No.	Section in manuscript	Comments/suggestions
8.	Others	Overall, the language and terminologies used are appropriate. The structure of the sentences aligns well with scientific writing.

Review completed on 17 March 2026
