# Supplementary material for: Prevalence and risk factors of adverse work outcomes among manufacturing workers with Long COVID in Malaysia – A nationwide study
Source: PLoS One. 2026 Sep 11;21(9):e0357125. doi: 10.1371/journal.pone.0357125 (PMC13567834; doi:10.1371/journal.pone.0357125)
Supplement: Supplementary file 2 [file pone.0357125.s007.pdf]

PLOS One

Prevalence and risk factors of adverse work outcomes among manufacturing workers with Long COVID in Malaysia – a nationwide study

--Manuscript Draft--

**Manuscript Number:**	PONE-D-25-68765
**Article Type:**	Research Article
**Full Title:**	Prevalence and risk factors of adverse work outcomes among manufacturing workers with Long COVID in Malaysia – a nationwide study
**Short Title:**	Adverse work outcomes among manufacturing workers with Long COVID
**Corresponding Author:**	Sheng Qian Yew<br>National University of Malaysia: Universiti Kebangsaan Malaysia<br>Serdang, Selangor MALAYSIA
**Keywords:**	Work impact; Post COVID; manufacturer; employer; employee
**Abstract:**	Introduction: The high prevalence of Long COVID among manufacturing workers, along with its impact on multiple organs, has raised concerns about the long-term sustainability of this industrial sector. Therefore, this study seeks to determine the prevalence and risk factors of adverse work outcomes among Malaysian manufacturing workers affected by Long COVID.<br><br>Materials and Methods: A cross-sectional study was conducted among 797 manufacturing workers across the country. Long COVID Questionnaire, Work-Related Quality of Life (WRQoL) Scale, and the Work Productivity and Activity Impairment (WPAI) Questionnaire, were utilied to assess the prevalence and risk factors of adverse work outcomes. Simple logistic regression and multiple logistic regression were used to determine the association between the various risk factors (i.e., socio-clinical, Long COVID, work, organisational, and personal factors) and adverse work outcomes.<br><br>Results: A majority (75.3%) of the workers with Long COVID demonstrates high WRQoL despite their illness. A total of 42.4% of workers with Long COVID had issues of sickness presenteeism (42.4%). Only 12.8% of the Long COVID workers had sickness absenteeism. Socio-clinical factors (i.e., increased in age, female gender, and smoking), Long COVID factors (i.e., increased number of Long COVID symptoms), work factors (i.e., engaged in shift work, handling of chemical hazardous to health, exposure to confined space entry, and exposure to excessive noise), organisational factors (i.e., absence of sickness absence policy) were significant risk factors of work outcomes among workers with Long COVID. Meanwhile, personal factor (i.e., informing employers about the Long COVID symptom) had protective effect on adverse work outcomes.<br><br>Conclusions: While most Long COVID workers reported high WRQoL, many experience sickness presenteeism and sickness absenteeism, with adverse work outcomes associated with socio-clinical, Long COVID, work and organisational factors. Personal factors appear to mitigate the adverse work outcomes.
**Order of Authors:**	Hanizah Mohd Yusoff
	Sheng Qian Yew
	Azmawati Mohd Nawi
	Noorlaili Mohd Tohit
	Nor Ba'yah Abdul Kadir
	Anita Abd Rahman
	Rahmat Dapari
	Ohnmar Htwe

Powered by Editorial Manager® and ProduXion Manager® from Aries Systems Corporation

	Muhamad Hazizi Muhamad Hasani
	Sivarajan Ramasamy
	Zuraida Mohamad
	Radzi Abdul Rashid
	Mazrura Sahani
	Azuraliza Abu Bakar
	Ida Rosnita Ismail
	Muhamad Ariff Muhamad Noordin
	Fazlin Mohd
	Amalia Sakina
	Kamilah Amnani
**Additional Information:**	
**Question**	**Response**
**Financial Disclosure**<br><br>Enter a financial disclosure statement that describes the sources of funding for the work included in this submission. Review the submission guidelines for detailed requirements. View published research articles from *PLOS ONE* for specific examples.<br><br>This statement is required for submission and **will appear in the published article** if the submission is accepted. Please make sure it is accurate.<br><br>**Funded studies**<br>Enter a statement with the following details:<br>• Initials of the authors who received each award<br>• Grant numbers awarded to each author<br>• The full name of each funder<br>• URL of each funder website<br>• Did the sponsors or funders play any role in the study design, data collection and analysis, decision to publish, or preparation of the manuscript?<br><br>Did you receive funding for this work?	Yes
Please add funding details.<br>as follow-up to "**Financial Disclosure**	This work was supported by the National Institute of Occupational Safety and Health (NIOSH) Malaysia [grant number: UKMP-S230424] to [HMY].

Enter a financial disclosure statement that describes the sources of funding for the work included in this submission. Review the submission guidelines for detailed requirements. View published research articles from *PLOS ONE* for specific examples.<br><br>This statement is required for submission and **will appear in the published article** if the submission is accepted. Please make sure it is accurate.<br><br>**Funded studies**<br>Enter a statement with the following details:<br>• Initials of the authors who received each award<br>• Grant numbers awarded to each author<br>• The full name of each funder<br>• URL of each funder website<br>• Did the sponsors or funders play any role in the study design, data collection and analysis, decision to publish, or preparation of the manuscript?<br><br>Did you receive funding for this work?"	
Please select the country of your main research funder (please select carefully as in some cases this is used in fee calculation).<br>as follow-up to "**Financial Disclosure**<br><br>Enter a financial disclosure statement that describes the sources of funding for the work included in this submission. Review the submission guidelines for detailed requirements. View published research articles from *PLOS ONE* for specific examples.<br><br>This statement is required for submission and **will appear in the published article** if the submission is accepted. Please make sure it is accurate.	MALAYSIA - MY

**Funded studies**<br>Enter a statement with the following details:<br>• Initials of the authors who received each award<br>• Grant numbers awarded to each author<br>• The full name of each funder<br>• URL of each funder website<br>• Did the sponsors or funders play any role in the study design, data collection and analysis, decision to publish, or preparation of the manuscript?<br><br>Did you receive funding for this work?"	
**Competing Interests**<br><br>Use the instructions below to enter a competing interest statement for this submission. On behalf of all authors, disclose any competing interests that could be perceived to bias this work—acknowledging all financial support and any other relevant financial or non-financial competing interests.<br><br>This statement is required for submission and **will appear in the published article** if the submission is accepted. Please make sure it is accurate and that any funding sources listed in your Funding Information later in the submission form are also declared in your Financial Disclosure statement.<br><br>View published research articles from *PLOS ONE* for specific examples.	The authors declare that they have no competing interests.

Powered by Editorial Manager® and ProduXion Manager® from Aries Systems Corporation

**NO authors have competing interests**<br><br>Enter: *The authors have declared that no competing interests exist.*<br><br>**Authors with competing interests**<br><br>Enter competing interest details beginning with this statement:<br><br>*I have read the journal's policy and the authors of this manuscript have the following competing interests: [insert competing interests here]*<br><br>* typeset	
**Ethics Statement**<br><br>Enter an ethics statement for this submission. This statement is required if the study involved:<br><br>• Human participants<br>• Human specimens or tissue<br>• Vertebrate animals or cephalopods<br>• Vertebrate embryos or tissues<br>• Field research<br><br>Write "N/A" if the submission does not require an ethics statement.<br><br>General guidance is provided below. Consult the submission guidelines for detailed instructions. **Make sure that all information entered here is included in the Methods section of the manuscript.**	Ethical approval for this study was obtained from the Research Ethics Committee of the National University of Malaysia (JEP-2023-607) and the Medical Research and Ethics Committee (MREC) of the Ministry of Health Malaysia (NMRR ID-23-03310-H3E). All participants provided written consents prior to their participations.

Powered by Editorial Manager® and ProduXion Manager® from Aries Systems Corporation

**Format for specific study types**<br><br>**Human Subject Research (involving human participants and/or tissue)**<br>• Give the name of the institutional review board or ethics committee that approved the study<br>• Include the approval number and/or a statement indicating approval of this research<br>• Indicate the form of consent obtained (written/oral) or the reason that consent was not obtained (e.g. the data were analyzed anonymously)<br><br>**Animal Research (involving vertebrate animals, embryos or tissues)**<br>• Provide the name of the Institutional Animal Care and Use Committee (IACUC) or other relevant ethics board that reviewed the study protocol, and indicate whether they approved this research or granted a formal waiver of ethical approval<br>• Include an approval number if one was obtained<br>• If the study involved *non-human primates*, add *additional details* about animal welfare and steps taken to ameliorate suffering<br>• If anesthesia, euthanasia, or any kind of animal sacrifice is part of the study, include briefly which substances and/or methods were applied<br><br>**Field Research**<br><br>Include the following details if this study involves the collection of plant, animal, or other materials from a natural setting:<br>• Field permit number<br>• Name of the institution or relevant body that granted permission	
**Data Availability**<br><br>Authors are required to make all data underlying the findings described fully available, without restriction, and from the time of publication. PLOS allows rare exceptions to address legal and ethical concerns. See the PLOS Data Policy and FAQ for detailed information.	No - some restrictions will apply

A Data Availability Statement describing where the data can be found is required at submission. Your answers to this question constitute the Data Availability Statement and **will be published in the article**, if accepted.<br><br>**Important:** Stating 'data available on request from the author' is not sufficient. If your data are only available upon request, select 'No' for the first question and explain your exceptional situation in the text box.<br><br>Do the authors confirm that all data underlying the findings described in their manuscript are fully available without restriction?	
**Describe where the data may be found in full sentences. If you are copying our sample text, replace any instances of XXX with the appropriate details.**<br><br>• If the data are **held or will be held in a public repository**, include URLs, accession numbers or DOIs. If this information will only be available after acceptance, indicate this by ticking the box below. For example: *All XXX files are available from the XXX database (accession number(s) XXX, XXX.).*<br>• If the data are all contained **within the manuscript and/or Supporting Information files**, enter the following: *All relevant data are within the manuscript and its Supporting Information files.*<br>• If neither of these applies but you are able to provide **details of access elsewhere**, with or without limitations, please do so. For example:<br><br>*Data cannot be shared publicly because of [XXX]. Data are available from the XXX Institutional Data Access / Ethics Committee (contact via XXX) for researchers who meet the criteria for access to confidential data.*<br><br>*The data underlying the results presented in the study are available from (include the name of the third party*	Data cannot be shared publicly because it consists of current and past medical history of the workers, which may affect their employment. Data are available from the Research Ethics Committee of the National University of Malaysia (contact via +603-9145 5046 / +603-9145 5048) for researchers who meet the criteria for access to confidential data.

Powered by Editorial Manager® and ProduXion Manager® from Aries Systems Corporation

and contact information or URL).<br>• This text is appropriate if the data are owned by a third party and authors do not have permission to share the data.<br><br>* typeset	
Additional data availability information:	

Powered by Editorial Manager® and ProduXion Manager® from Aries Systems Corporation

Manuscript Click here to access/download;Manuscript;Manuscript.docx

Full Title: Prevalence and risk factors of adverse work outcomes among manufacturing workers with Long COVID in Malaysia – a nationwide study

Short Title: Adverse work outcomes among manufacturing workers with Long COVID

Hanizah Mohd Yusoff [a], Yew Sheng Qian [a]*, Azmawati Mohd Nawi [a], Noorlaili Mohd Tohit [b], Nor Ba'yah Abdul Kadir [c], Anita Abd Rahman [d], Rahmat Dapari [d], Ohnmar Htwe [e], Muhamad Hazizi Muhamad Hasani [f], Sivarajan Ramasamy [f], Zuraida Mohamad [f], Radzi Abdul Rashid [g], Mazrura Sahani [h], Azuraliza Abu Bakar [i], Ida Rosnita Ismail [j], Muhamad Ariff Muhamad Noordin [k], Fazlin Mohd [l], Amalia Sakina [l], Kamilah Amnani [l]

[a] Department of Public Health Medicine, Faculty of Medicine, Universiti Kebangsaan Malaysia, Jalan Yaacob Latif, Bandar Tun Razak, 56000 Cheras, Kuala Lumpur, Malaysia.

[b] Department of Family Medicine, Faculty of Medicine, Universiti Kebangsaan Malaysia, Jalan Yaacob Latif, Bandar Tun Razak, 56000 Cheras, Kuala Lumpur, Malaysia.

[c] Faculty of Social Sciences and Humanitarian, Universiti Kebangsaan Malaysia, 43600, Bangi, Selangor, Malaysia.

[d] Faculty of Medicine and Health Science, Universiti Putra Malaysia, 43400 UPM Serdang, Selangor, Malaysia.

[e] Faculty of Medicine, Universiti Sultan Zainal Abidin Medical, Jalan Sultan Mahmud 20400 Kuala Terengganu, Terengganu, Malaysia.

[f] Negeri Sembilan State Health Department, Jalan Rasah, Bukit Rasah, 70300 Seremban, Negeri Sembilan, Malaysia.

[g] OSH ProCare Consultant and Services, G&C Township, 44, Jln RK 6/10, 1388 Rasah-Kemayan, 70300 Seremban, Negeri Sembilan, Malaysia.

[h] Centre of Health and Applied Sciences, Faculty of Health Sciences, National University of Malaysia, Jalan Raja Muda Abdul Aziz, 50300 Kuala Lumpur, Malaysia.

[i] Faculty of Information Science and Technology, Universiti Kebangsaan Malaysia, 43600 UKM, Bangi, Selangor, Malaysia.

[j] Graduate School of Business, Universiti Kebangsaan Malaysia, 43600 Bangi, Selangor, Malaysia.

[k] Research Management Center, National Institute of Occupational Safety and Health, Lot 1, Jalan 15/1, Section 15, 43650 Bandar Baru Bangi, Selangor, Malaysia.

[l] UKM Pakarunding Sdn Bhd, Level 3, Universiti Kebangsaan Malaysia, Bangunan Wawasan, 43600 Bangi, Selangor, Malaysia.

Correspondence:

Sheng Qian Yew

Email: shengqian@ukm.edu.my

Tel: 03-91458792

Address: Department of Public Health Medicine, Faculty of Medicine, Universiti Kebangsaan Malaysia, Jalan Yaacob Latif, Bandar Tun Razak, 56000 Cheras, Kuala Lumpur, Malaysia.

Abstract

Introduction: The high prevalence of Long COVID among manufacturing workers, along with its impact on multiple organs, has raised concerns about the long-term sustainability of this

industrial sector. Therefore, this study seeks to determine the prevalence and risk factors of adverse work outcomes among Malaysian manufacturing workers affected by Long COVID.

Materials and Methods: A cross-sectional study was conducted among 797 manufacturing workers across the country. Long COVID Questionnaire, Work-Related Quality of Life (WRQoL) Scale, and the Work Productivity and Activity Impairment (WPAI) Questionnaire, were utilied to assess the prevalence and risk factors of adverse work outcomes. Simple logistic regression and multiple logistic regression were used to determine the association between the various risk factors (i.e., socio-clinical, Long COVID, work, organisational, and personal factors) and adverse work outcomes.

Results: A majority ~~(75.3%)~~ of the workers with Long COVID demonstrates high WRQoL despite their illness. A total of 42.4% of workers with Long COVID had issues of sickness presenteeism ~~(42.4%)~~. Only 12.8% of the Long COVID workers had sickness absenteeism. Socio-clinical factors (~~i.e.~~, increased in age, female gender, and smoking), Long COVID factors (i.e., increased number of Long COVID symptoms), work factors (i.e., ~~engaged in~~ shift work, handling ~~of~~ chemical hazardous to health, exposure to confined space entry, and exposure to excessive noise), organisational factors (i.e., absence of sickness absence policy) were significant risk factors of work outcomes among workers with Long COVID. Meanwhile, personal factor (~~i.e.,~~ informing employers about the Long COVID symptom) had protective effect on adverse work outcomes.

Conclusions: While most Long COVID workers reported high WRQoL, many experience sickness presenteeism and sickness absenteeism, with adverse work outcomes associated with

socio-clinical, Long COVID, work and organisational factors. Personal factors appear to mitigate the adverse work outcomes.

Keywords: Work impact, Post COVID, manufacturer, employer, employee

1. Introduction

The manufacturing sector is a key contributor to Malaysia's economy, accounting for the second-largest share of the country's gross domestic product (GDP) and an annual contribution exceeding RM300 billion (1). Furthermore, it provides employment for approximately 2.33 million individuals as of November 2022 (2). The industry's reliance on skilled labour is crucial for its efficient operation, making the retention of talented, skilled, and experienced workers essential for enhancing overall performance, quality, efficiency, effectiveness, and productivity within the sector (3). Consequently, maintaining a healthy and productive workforce is vital. Unfortunately, the high number of COVID-19 clusters among manufacturing workers during the pandemic, along with the subsequent high prevalence of Long COVID, has raised concerns regarding the long-term sustainability of this sector.

The World Health Organization (WHO) defines Long COVID as a condition that develops in individuals with a prior SARS-CoV-2 infection, typically occurring three months after the initial onset of COVID-19 (4). Additionally, the symptoms must persist for at least two months and cannot be attributed to any other underlying condition. Generally, Long COVID presents with a wide range of symptoms, which are thought to result from a pathology similar to that of acute

COVID-19. Some of the most commonly reported symptoms include fatigue, shortness of breath, and cognitive impairment (5).

Given its impact on multiple organ systems, Long COVID can significantly affect daily functioning, including workplace activities. For instance, workers experiencing Long COVID may face various negative work-related consequences, such as decreased productivity and performance, absenteeism, presenteeism, a higher risk of occupational injuries, and a reduced quality of work life (6, 7). Furthermore, workers requiring prolonged medical leave for recovery, ongoing treatment, or those unable to return to work may contribute to increased operational expenses for employers. It is also worth mentioning that the extent of these adverse work outcomes may vary depending on several factors, including the availability of workplace adjustment, company policies on sick leave, and return-to-work strategies implemented within the organisation.

While previous research has documented various negative effects of Long COVID, most studies have primarily focused on the general population (8, 9). Consequently, there is limited knowledge regarding the specific impact of Long COVID on work-related outcomes among manufacturing workers. An updated study on the burden of Long COVID within this sector would help workers, employers, policymakers, and other stakeholders better understand the scale of the issue affecting the manufacturing industry. Based on these research gaps and underlying justifications, this study aims to assess the prevalence and risk factors associated with adverse work outcomes among manufacturing workers in Malaysia who are affected by Long COVID.

2. Materials and Methods

A cross-sectional study was conducted from April 2024 to October 2024 across the entire Malaysia. The target population consisted of workers from manufacturing companies listed in the Federation of Malaysian Manufacturers (FMM) directory, with eligibility criteria aligned with those specified in Table 1. Initially, a multistage sampling approach was intended for selecting manufacturing factories. However, due to a very low response rate, the researchers opted to use a convenience sampling method instead. This was followed by a universal sampling to select workers from each factory. Given that precision level = 0.05, confidence level = 95%, prevalence of adverse work outcome = 50.0% (in the absence of data on prevalence of adverse work outcome among workers with Long COVID), non-response rate = 50%, the total sample size required across the nation was 768. This sample size estimation was calculated using Kish's formula (10).

Table 1: Inclusion and exclusion criteria of the manufacturing workers

Inclusion Criteria	Exclusion Criteria
1. Workers of the manufacturing company (including electrical, electronics, petroleum, chemical, rubber, plastic, non-metallic mineral products, basic metal, fabricated metal, food and beverages, tobacco, wood, furniture, paper products, printing, textile, wearing apparel, leather, footwear, and transport equipment sectors).	1. Workers who had resigned or terminated.<br>2. Temporary workers.<br>3. Pregnant women during data collection or during acute phase of COVID-19.<br>4. Workers who did not consent for participation.

2. Workers diagnosed of COVID-19 from 1st January 2022 until 30th April 2024.
3. Malaysian and legal foreign worker.
4. Worked for at least six months prior to the acute phase of COVID-19.
5. Able to communicate in English and/or Malay languages.

Three previously validated questionnaires, namely the Long COVID Questionnaire, Work-Related Quality of Life (WRQoL) Scale (11), and the Work Productivity and Activity Impairment (WPAI) Questionnaire (12), were utilised to assess the prevalence and risk factors of adverse work outcomes. Permission to use these questionnaires was obtained from their respective developers.

The 67-item Long COVID Questionnaire was used to evaluate both the presence of Long COVID symptoms and the associated adverse work outcomes among manufacturing workers (13). The questionnaire is divided into three sections. Section A comprises seven items designed to collect socio-clinical data. Section B contains 57 items assessing the presence of Long COVID, where a worker is classified as having Long COVID if they respond with "Yes," "Yes," and "No" for parts a, b, and c of each symptom listed. Lastly, Section C consists of three items aimed at identifying workplace safety concerns. A worker is considered to have safety issues at work if they select "unsure," "affected," or "very much affected" for at least one item in this section.

The 24-item Work-Related Quality of Life (WRQoL) Scale was used to assess the perceived quality of life of the manufacturing workers with Long COVID (11). WRQoL was measured through six psychosocial subscales (i.e., general well-being, home-work interface, job-career satisfaction, control at work, working conditions, and stress at work). For score interpretation, items 7, 9, and 19 are reverse-coded. In this study, the total WRQoL score is derived by summing all 24 item scores, with a score of 83 or higher indicating a high quality of work life, while a score below 83 is classified as low WRQoL.

The 6-item, unidimensional Work Productivity and Activity Impairment (WPAI) Questionnaire was employed to evaluate sickness absenteeism and presenteeism among manufacturing workers affected by Long COVID (12). A worker is classified as having sickness absenteeism if they missed at least 1% of their assigned work time in the past seven days. Likewise, sickness presenteeism is identified when a worker reports at least a 1% reduction in work performance due to health issues within the same period.

Data collection involved on-site visits by researchers to the selected manufacturing companies across the entire nation. These companies were requested to provide a list of workers who had been diagnosed with and documented as having acute COVID-19. With the cooperation of the management, the selected workers were assembled in a designated room within the management office. Each worker was then screened for eligibility based on the criteria outlined in Table 1. Eligible workers were provided with a briefing on the study objectives, instructions for questionnaire administration, and their rights before providing informed consent to participate. Those who were deemed ineligible or who declined participation were thanked and permitted to

return to their work. Prior to commencing the survey, informed consent was obtained from all eligible workers. They were encouraged to complete the questionnaire with the assistance of the researchers, and sufficient time was allocated to ensure high-quality data collection. Workers' anonymity and confidentiality were upheld throughout the study. To minimise disruptions to workplace operations, data collection was conducted in a staggered manner, with only one to two workers completing the questionnaire at a time.

Descriptive analysis was conducted to present the prevalence of adverse work outcomes and risk factors of adverse work outcomes. Simple logistic regression was performed to examine the association between the various risk factors (including socio-clinical, Long COVID, work, organisational, and personal factors) and adverse work outcomes. Risk factors with a p-value < 0.25 were included in a multiple logistic regression model. Additionally, the moderating effects of organisational and personal factors on Long COVID symptoms were assessed using multiple logistic regression. All statistical analyses were performed using SPSS version 28.

Ethical approval for this study was obtained from the Research Ethics Committee of the National University of Malaysia (JEP-2023-607) and the Medical Research and Ethics Committee (MREC) of the Ministry of Health Malaysia (NMRR ID-23-03310-H3E). Participants recruitment began at 15th January 2024 and ended on 16th December 2024. All participants provided written informed consent prior to their participation.

3. Results

In the present study, adverse work outcomes included poor work-related quality of life, work productivity (sickness absenteeism and sickness presenteeism), and safety issues at work (Table 1). In terms of the prevalence of adverse work outcomes, workers with Long COVID exhibited a high work-related quality of life, with 75.3% maintaining high level despite their health condition. Sickness absenteeism was reported by only 12.8% of workers with Long COVID. Total of 42.4% of the workers experienced sickness presenteeism. Only a small number of workers (6.3%) reported experiencing safety issues at work.

Table 1: Prevalence of adverse work outcomes among the manufacturing workers with Long COVID

**Adverse Work Outcomes**	**Workers with LC, n (%)**<br>**N = 797**
**Work-Related Quality of Life (WRQOL)**	
Low	197 (24.7)
High	600 (75.3)
**Work Productivity (Sickness Absenteeism)**	
Yes	102 (12.8)
No	695 (87.2)
**Work Productivity (Sickness Presenteeism)**	
Yes	338 (42.4)
No	459 (57.6)
**Safety Issues at Work**	

Yes	50 (6.3)
No	747 (93.7)

The risk factors of adverse work outcomes investigated in the present study include socio-clinical, Long COVID, work, organisational, and personal factors. The socio-clinical factors of the manufacturing workers with Long COVID were presented in Table 3. The mean age of the workers was 37.4 ± 9.5 years old. Most workers were females (54.5%), overweight (37.6%) and did not have co-morbidities (64.4%). Among those with co-morbidities, hypertension (13.2%) was the most common, followed by asthma (7.8%) and diabetes (5.4%). Additionally, the majority of workers were non-smokers (78.2%).

Table 3: Socio-clinical factors of the manufacturing workers with Long COVID

**Individual Factors of the Manufacturing Workers**	**Mean (SD)**	**n (%) N = 797**
**Age (years old)**	37.4 (9.5)	-
**Gender**		
Male	-	363 (45.5)
Female	-	434 (54.5)
**BMI Category (kg/m^2)**		
< 18.5 (underweight)	-	38 (4.8)
18.5 – 24.9 (normal)	-	276 (34.6)
25.0 – 29.9 (overweight)	-	300 (37.6)

> 30 (obese)	-	183 (23.0)
**Co-morbidities**		
Total co-morbidities (may overlap)	-	284 (35.6)
Hypertension	-	105 (13.2)
Asthma	-	62 (7.8)
Diabetes	-	43 (5.4)
Anxiety/Panic Attack	-	16 (2.0)
Heart disease	-	11 (1.4)
Autoimmune disease	-	10 (1.3)
Chronic kidney disease	-	4 (0.5)
Chronic liver disease	-	1 (0.1)
Cancer	-	0 (0.0)
HIV Infection	-	0 (0.0)
Others[†]	-	94 (11.8)
**Smoking Status**		
Non-smokers	-	623 (78.2)
Smokers	-	174 (21.8)

**Range of age = 22 – 63 years old*

[†]*Others include gastritis, dyslipidaemia, gout, and psoriasis.*

As presented in Table 4, among the 797 manufacturing workers with Long COVID, most common symptoms for Long COVID were post-exertional malaise (50.9%), followed by fatigue (37.4%),

and joint pain (25.6%). Of note, the workers experienced a plethora of symptoms, ranging from one symptom to 19 symptoms, with a majority of them experienced only one symptom (35.3%).

Table 4: Long COVID factors of the manufacturing workers

Long Covid Symptoms	n (%)<br>N = 797
Post-exertional malaise	406 (50.9)
Fatigue	298 (37.4)
Joint Pain	204 (25.6)
Muscle pain	171 (21.5)
Cough	164 (20.6)
Headache	142 (17.8)
Tingling	140 (17.6)
Cognitive Dysfunction	136 (17.1)
Dyspnoea	133 (16.7)
Tachycardia	133 (16.7)
Chest Pain	120 (15.1)
Sleeping Disturbance	105 (13.2)
Intermittent Fever	93 (11.7)
Dizziness	91 (11.4)
Tinnitus	77 (9.7)
Diarrhoea	55 (6.9)

Altered Sensation	39 (4.9)
Abdominal Pain	27 (3.4)
Depression	26 (3.3)
**No. of Long COVID Symptom(s) Experienced**	
1	281 (35.3)
2	158 (19.8)
3	109 (13.7)
4	76 (9.5)
5	42 (5.3)
6	38 (4.8)
7	22 (2.8)
8	18 (2.3)
9	17 (2.1)
10	13 (1.6)
11	6 (0.8)
12	5 (0.6)
13	7 (0.9)
14	1 (0.1)
15	2 (0.2)
16	1 (0.1)
17	0 (0.0)
18	1 (0.1)
19	0 (0.0)

Table 5 presents the work factors of the manufacturing workers with Long COVID. Most of the workers interviewed were working in the "electrical and electronics" subsectors (52.3%) and most of them were working under the "operation/supply chain" department (84.8%)". Additionally, most of the workers were operators (40.8%) and primarily worked normal office hours (51.9%). In terms of types of hazards at work, the most commonly identified workplace hazards included visual display units (VDU) (61.9%), manual handling (40.8%), and excessive noise (29.5%).

Table 5: Work factors of the manufacturing workers with Long COVID

**Work Factors of the Manufacturing Workers**	**n (%)**<br>**N = 797**
**Industrial Subsectors**	
Electrical and Electronics	417 (52.3)
Transport, Vehicle and Equipment	163 (20.5)
Rubber	119 (14.9)
Textile, Wearing Apparel and Leather	74 (9.3)
Chemical (including Petroleum)	11 (1.4)
Paper, Printing, and Publishing	10 (1.3)
Food, Beverage, and Tobacco	3 (0.4)
**Working Departments**	
Operations/Supply Chain	676 (84.8)
Research and Development (R&D)	29 (3.6)

Human Resource	23 (2.9)
Finance (Including Accounts)	20 (2.5)
Facilities	17 (2.1)
Sales	8 (1.0)
Procurement	8 (1.0)
Marketing	5 (0.6)
Corporate	3 (0.4)
Others	8 (1.0)
**Positions at Company**	
Operator	325 (40.8)
Technician, Specialist or Permit Holder	199 (25.0)
Executive	77 (9.7)
Supervisor	74 (9.3)
Officer	47 (5.9)
Manager	46 (5.8)
Clerk	15 (1.9)
General Worker	5 (0.6)
Support Services	4 (0.5)
Director	1 (0.1)
Others	4 (0.5)
**Work Schedule**	
Normal/Office hours	414 (51.9)
Shift including Staggered Shift	382 (47.9)

Flexible	1 (0.1)
Work from Home	0 (0.0)
**Types of Hazards at Work**	
VDU (Visual Display Unit) / Video (Computer)	493 (61.9)
Manual Handling	325 (40.8)
Excessive Noise	235 (29.5)
Handling of Chemical Hazardous to Health	206 (25.8)
Electrical Work	158 (19.8)
Working in extreme temperatures (hot or cold)	99 (12.4)
Confined Space Entry	91 (11.4)
Working at Heights	60 (7.5)
Lone / Isolated Work	45 (5.6)
Driving Vehicle	35 (4.4)
Working on Roofs	33 (4.1)
Construction Work	30 (3.8)
Driving Lift Truck	28 (3.5)
†Others	40 (5.0)

†*Example: Working with sharp objects and risk of falling objects*

Analysis of the organisational factors found that 53.3% of the workers with Long COVID perceived presence of work adjustment feasibility at their workplaces (Table 6). Furthermore, almost half (49.4%) of the workers with Long COVID perceived that a sickness absence policy

was present at their workplace. Only 32.9% of the workers believed that RTW practices were present at their workplace.

Table 6: Organisational factors of manufacturing workers with Long COVID symptoms

Organisational Factors of Manufacturing Workers with Long COVID Symptoms	n (%) N = 797	
	Yes (%)	No (%)
Presence of Work Adjustment Feasibility	425 (53.3)	372 (46.7)
Presence of Sickness Absence Policy at Workplace	394 (49.4)	403 (50.6)
Presence of RTW Practices at Workplace	262 (32.9)	535 (67.1)

In terms of personal factor, most of the workers with Long COVID had good work attitude (93.9%), despite suffering the condition (Table 7). Among the workers with Long COVID symptoms, a majority of them did not inform their employers (89.1%).

Table 7: Personal factors of manufacturing workers with Long COVID symptoms

Personal Factors of Manufacturing Workers with Long COVID Symptoms	Workers with LC, n (%) N = 797
**Work Attitude**	
Good	748 (93.9)
Poor	49 (6.1)

**Informed Employer on Long Covid**	
Yes	87 (10.9)
No	710 (89.1)

To investigate the association with WRQoL, a total of 21 risk factors (i.e., socio-clinical, Long COVID, work, organisational, and personal factors with $p<0.25$ in the simple logistic regression) were modelled using a multiple logistic regression (MLR) with forward method (Appendix 1). All assumptions of MLR (i.e., linearity, interaction, multicollinearity, and outliers) were met. Out of the 21 risk factors, only five risk factors were significantly associated with WRQoL, namely age, smoking status, excessive noise, confined space entry and sickness absence policy at workplace. For workers with Long COVID symptoms, each additional year of age is associated with a 2% increase in the odds of experiencing poor WRQOL (aOR = 1.02, $p < 0.001$). Workers with Long COVID symptoms who smoke have 1.38 higher odds of suffering from poor WRQOL (aOR = 1.38, $p < 0.001$), compared to those who were not smoking. Workers with Long COVID symptoms who were exposed to excessive noise had 1.55 times higher odds of suffering from poor WRQOL (aOR = 1.55, p = <0.001), compared to those who were unexposed. Workers with Long COVID symptoms subjected to confined space entry had 1.60 times higher odds of suffering from poor WRQOL (aOR = 1.60, $p = 0.013$), compared to those who were unexposed. Workers who have experienced a no sickness absence policy at the workplace had 2.77 times higher odds of suffering from poor WRQOL (aOR = 2.77, $p < 0.001$), compared to those who experienced sickness absence policy.

To investigate the association with sickness absenteeism, a total of 10 risk factors (i.e., socio-clinical, Long COVID, work, organisational, and personal factors with $p<0.25$ in the simple logistic regression) were modelled using a multiple logistic regression (MLR) with forward method (Appendix 2). All assumptions of MLR (i.e., linearity, interaction, multicollinearity, and outliers) were met. Out of the 10 risk factors, only manual handling was significantly associated with sickness absenteeism. Workers with Long COVID symptoms who are exposed to manual handling had 1.33 times higher odds of having sickness absenteeism (aOR = 1.33, p = 0.037), compared to those who were unexposed.

To investigate the association with sickness presenteeism, a total of 17 risk factors (i.e., socio-clinical, Long COVID, work, organisational, and personal factors with $p<0.25$ in the simple logistic regression) were modelled using a multiple logistic regression (MLR) with forward method (Appendix 3). All assumptions of MLR (i.e., linearity, interaction, multicollinearity, and outliers) were met. Out of the 17 risk factors, only three risk factors were significantly associated with sickness presenteeism, namely work schedule, number of Long COVID symptoms, and informing employer regarding the Long COVID symptoms. Workers with Long COVID symptoms who engaged in shift work had 1.25 higher odds of having sickness presenteeism (aOR = 1.25, p = 0.027), compared to those who engaged in normal working hours. The odds of having sickness presenteeism increase by 1.15 for every increase in the number of Long COVID symptoms. Informing the employer regarding the Long COVID symptoms has been found to be a protective factor against sickness presenteeism where they had 42% lower odds of having sickness presenteeism (aOR = 0.58, p = 0.019), compared to those who did not inform.

To investigate the association with safety issues at work, a total of 26 risk factors (i.e., socio-clinical, Long COVID, work, organisational, and personal factors with $p<0.25$ in the simple logistic regression) were modelled using a multiple logistic regression (MLR) with forward method (Appendix 4). All assumptions of MLR (i.e., linearity, interaction, multicollinearity, and outliers) were met. Out of the 26 risk factors, only two risk factors were significantly associated with safety at work, namely handling of chemicals hazardous to health and informed employer regarding Long COVID symptoms. Workers with Long COVID symptoms who handled chemical hazardous to health had 2.51 times higher odds of having safety issues at work (aOR = 2.51, $p < 0.001$), compared to those who did not. Informing the employer regarding their Long COVID symptoms has been found to be a protective factor against safety issues at work where they had 68% lower odds of having safety issues at work (aOR = 0.32, $p = 0.007$), compared to those who did not inform.

According to the multiple logistic regression, it was found that none of the organisational factors and personal factors had moderating effect on the relationship between Long COVID and adverse work outcomes (Appendix 5).

4. Discussion

Our finding posited that a relatively low proportion (almost only a quarter) of workers with Long COVID had poor work-related quality of life. In fact, a recent study also found that there were no significant differences in the quality of life between those with and without Long COVID (14). It

can be concluded that although Long COVID symptoms could affect multiple body systems, these symptoms are manageable and have little impact on work-related quality of life.

It is noteworthy that although only 12.8% of the workers with Long COVID absent from their work due to the symptoms, a relatively large proportions of workers (42.4%) continue to work despite having Long COVID symptoms. This may lead to reduced productivity, prolonged recovery times, and potential worsening of symptoms due to the lack of rest and proper medical care (15). This highlights the importance of workplace accommodations, such as flexible work hours, remote work options, or lighter workloads, to support employees dealing with persistent symptoms and help them achieve a full recovery while maintaining their employment.

Workers with Long COVID who are older, smokers, exposed to excessive noise, working in confined spaces, and without a sickness absence policy face higher odd of experiencing poor work-related quality of life due to several factors. Older workers often have weakened immune systems and slower recovery times (16), which can exacerbate fatigue and mobility issues, leading to reduced work capacity. Smoking worsens respiratory symptoms, reduces lung function, and slows the healing process (17), making recovery from Long COVID more challenging. Exposure to excessive noise can contribute to fatigue, stress, headaches, and sleep disturbances (18), all of which may amplify Long COVID symptoms. Additionally, working in confined spaces with poor ventilation can worsen Long COVID symptom especially within the respiratory system (19). Lastly, the absence of a sickness absence policy adds to the problem by preventing proper rest and recovery (20), as workers may feel pressured to continue working despite their symptoms, leading to stress and anxiety. All these factors can create a cycle of deteriorating health and diminished

work-related quality of life. As such, there is an urgent need for supportive workplace policies and accommodations to help mitigate risks and support recovery.

Workers with Long COVID symptoms who are exposed to manual handling have higher odds of sickness absenteeism due to the physical and physiological demands of their work. Chronic fatigue and muscle weakness, common in Long COVID, can make the physical exertion required for tasks like lifting, pushing, or pulling heavy objects difficult to sustain. Persistent joint and muscle pain can be worsened by manual handling (21), leading to the need for time off to recover. Respiratory symptoms, such as dyspnoea, further reduces stamina and make manual tasks particularly challenging (22). Additionally, post-exertional malaise, where symptoms worsen after physical activity (23), can prolong recovery and contribute to more frequent sickness absences. These factors highlight the need for workplace accommodations, such as task modifications or lighter duties, to help workers manage their symptoms and maintain their employment.

Workers with Long COVID symptoms who engage in shift work and experience a high number of symptoms are more likely to face sickness presenteeism. Shift work disrupts normal sleep patterns and can lead to chronic fatigue (24), which compounds Long COVID symptoms such as exhaustion, brain fog, and muscle pain. The irregular hours can also interfere with the workers' ability to recover, leading to persistent or worsened symptoms. However, many workers may continue to attend work despite their symptoms due to fear of job loss, financial insecurity, or a workplace culture that discourages absences. In addition, the high number of Long COVID symptoms further exacerbates sickness presenteeism, as these workers may be physically and/or mentally unfit to perform their duties effectively but continue working out of necessity or

obligation (25). On the other hand, informing the employer regarding the Long COVID symptoms has been found to be a protective factor against sickness presenteeism. When employers are aware of an employee's health condition, they are more likely to implement supportive measures such as flexible work arrangements, reduced workload, or modified duties tailored to the employee's physical and mental limitations (26). These accommodations can help reduce the pressure to attend work while unwell and allow for proper symptom management and recovery. Additionally, open communication can reduce fear of job loss or stigma associated with taking sick leave, creating a more supportive work environment.

Workers with Long COVID symptoms who handle hazardous chemicals may face higher odds of safety issues at work because fatigue and malaise can impair their ability to respond quickly to chemical hazards or emergencies (27). Additionally, cognitive impairments such as brain fog, poor memory, and difficulty concentrating increase the likelihood of errors in handling hazardous chemicals (28). On the other hand, workers with Long COVID who inform their employers about their symptoms may experience less safety issues. This is because informing employers allows workers to receive reasonable accommodations (29), such as adjusted workloads, flexible schedules, or reduced exposure to hazardous chemicals, which can help mitigate safety risks.

4.1 Strengths and Limitations of the Study

This study has several key strengths. Firstly, its nationwide coverage, which includes manufacturing workers from all states in Malaysia, enhances the generalisability of its findings. Secondly, to the researchers' best knowledge, this is the first study to determine the prevalence

and risk factors of adverse work outcomes among manufacturing workers, offering valuable insights for occupational health policies and interventions.

Although the study has notable strengths, it also has some limitations. A major limitation is its cross-sectional design, which prevents the establishment of causal relationships between risk factors and adverse work outcomes. Furthermore, the study relies on workers' recollection of COVID symptoms, which may introduce recall bias, as they might not accurately remember or report their symptoms and work-related experiences. While the study provides comprehensive insights into the manufacturing sector, its findings may not be applicable to other industries with distinct occupational environments and risk factors.

4.2 Recommendations for Future Studies

Future research should consider using a longitudinal study design to determine causal relationships between risk factors and adverse work outcomes. Additionally, integrating objective clinical assessments alongside self-reported data could help reduce recall bias and improve the accuracy of symptom reporting. This may include medical evaluations, workplace health monitoring, or biomarkers for Long COVID. To improve generalisability, future studies should expand beyond the manufacturing sector to include workers from various industries with different occupational exposures. This would provide a broader understanding of Long COVID's impact across work environments and support the development of industry-specific recommendations.

4.3 Study Implications

The findings of this study have important implications for workplace interventions and occupational health policies. Firstly, identifying key risk factors for adverse work outcomes underscores the need for targeted workplace interventions. Employers should implement preventive measures such as enhancing workplace ventilation, minimising exposure to hazardous chemicals, and addressing ergonomic risks associated with prolonged VDU use. Additionally, the study highlights the importance of early detection and management of Long COVID symptoms among workers, which could be facilitated through regular health screenings and medical support programs. Moreover, the findings suggest that the lack of a structured sickness absence policy may contribute to negative work outcomes. Employers should establish clear guidelines on sickness absence and return-to-work strategies to support employees recovering from Long COVID. Furthermore, the study indicates that disclosing Long COVID symptoms to employers may have a protective effect, emphasising the importance of fostering a supportive and open work environment. This calls for awareness campaigns and training programs to educate both employers and employees on the impact of Long COVID and the benefits of workplace accommodations.

The study also has wider implications for organisational health policies and regulations. Policymakers should incorporate Long COVID management into existing occupational health and safety frameworks to ensure adequate support for affected workers. Furthermore, based on the study's identification of specific risk factors, future workplace guidelines could include sector-specific recommendations to help reduce the impact of Long COVID.

5. Conclusions

Three-quarter of the manufacturing workers with Long COVID symptoms demonstrated high WRQOL despite their illness. About 4 out of 10 workers with Long COVID symptoms had issues of sickness presenteeism while about 1 in 10 workers of the Long COVID workers had issue of sickness absenteeism. A very small number of workers with Long COVID symptoms had safety issues at work. Individual factor (i.e., increased in age, female gender, and smoking), Long COVID symptoms (i.e., increased number of Long COVID symptoms), work factor (i.e., engaged in shift work, handling of chemical hazardous to health, exposure to confined space entry, and exposure to excessive noise), organisational factor (i.e., absence of sickness absence policy) were significant risk factors of adverse work outcomes among workers with Long COVID symptoms. Meanwhile, personal factor such as informing employers about the Long COVID symptom had protective effect on adverse work outcomes among the workers with Long COVID symptoms.

Declarations

Ethics approval and consent to participate: Ethical approval for this study was obtained from the Research Ethics Committee of the National University of Malaysia (JEP-2023-607) and the Medical Research and Ethics Committee (MREC) of the Ministry of Health Malaysia (NMRR ID-23-03310-H3E). All participants provided written consents prior to their participations. Participants recruitment began at 15th January 2024 and ended on 16th December 2024

Consent for publication: Not applicable.

Availability of data and materials: Data cannot be shared publicly because it consists of current and past medical history of the workers, which may affect their employment. Data are available from the Research Ethics Committee of the National University of Malaysia (contact via +603-9145 5046 / +603-9145 5048) for researchers who meet the criteria for access to confidential data.

Competing interests: The authors declare that they have no competing interests.

Funding: This work was supported by the National Institute of Occupational Safety and Health (NIOSH) Malaysia [grant number: UKMP-S230424] to [HMY].

Authors' contributions: HMY, SQY, MAMN, AAR, and RD conceptualised and designed the study. Data collection was conducted by MHMH, SR, ZM, RAR, MS, AAB, IRI, FM, AS, and KA. SQY, AMN, NBAK, RI, and NMT carried out the statistical analysis. SQY prepared the initial draft of the manuscript. All authors participated in interpreting the results, provided critical revisions for intellectual significance, and approved the final version for publication.

Acknowledgements: We would like to thank the Director of National Institute of Occupational Safety and Health (NIOSH) Malaysia, for their grant and permission to publish this article. As specified in the terms of reference, the study will focus exclusively on manufacturing workers.

References:

1. Dosm. Gross Domestic Product Malaysia. 2025.

2. Dosm. Department of Statistics Malaysia. 2023.

3. Alaaraj S, Mohamed ZA, Ahmad Bustamam U. Do Knowledge Management Capabilities Reduce the Negative effect of Environment Uncertainties on Organizational Performance? A Study of Public Listed Companies in Malaysia. International Journal of Economic Research. 2017;14:443-56.

4. Who. A clinical case definition of post COVID-19 condition by a Delphi consensus, 6 October 2021. 2021.

5. Soriano JB, Murthy S, Marshall JC, Relan P, Diaz JV, Condition WHOCCDWGoP-C-. A clinical case definition of post-COVID-19 condition by a Delphi consensus. The Lancet Infectious Diseases. 2022;22(4):e102-e7.

6. Gualano MR, Rossi MF, Borrelli I, Santoro PE, Amantea C, Daniele A, et al. Returning to work and the impact of post COVID-19 condition: A systematic review. Work (Reading, Mass). 2022;73(2):405-13.

7. Nehme M, Vieux L, Kaiser L, Chappuis F, Chenaud C, HealthCo Study T, et al. The longitudinal study of subjective wellbeing and absenteeism of healthcare workers considering post-COVID condition and the COVID-19 pandemic toll. Scientific Reports. 2023;13(1):10759.

8. Silva Andrade B, Siqueira S, de Assis Soares WR, de Souza Rangel F, Santos NO, dos Santos Freitas A, et al. Long-COVID and Post-COVID Health Complications: An Up-to-Date Review on Clinical Conditions and Their Possible Molecular Mechanisms. Viruses. 2021;13(4):700.

9. Aiyegbusi OL, Hughes SE, Turner G, Rivera SC, McMullan C, Chandan JS, et al. Symptoms, complications and management of long COVID: a review. J R Soc Med. 2021;114(9):428-42.

10. Kish L. Survey Sampling: Wiley; 1995 1995.

11. Chen W-S, Haniff J, Siau C-S, Seet W, Loh S-F, Abd Jamil MH, et al. Psychometric Properties of the Malay Work-Related Quality of Life (WRQoL) Scale in Malaysia. World Journal of Social Science Research. 2014;1(1):57.

12. Reilly MC, Zbrozek AS, Dukes EM. The validity and reproducibility of a work productivity and activity impairment instrument. PharmacoEconomics. 1993;4(5):353-65.

13. Mohd Yusoff H, Yew SQ, Mohammed Nawi A, Mohd Tohit N, Abd Rahman A, Dapari R, et al. Development and Validation of the Long COVID Questionnaire to Determine the Prevalence and Risk Factors of Long COVID among Manufacturing Workers in Malaysia – A Mixed-Methods Study. Journal of Occupational Safety and Health. In press.

14. Sun C, Liu Z, Li S, Wang Y, Liu G. Impact of Long COVID on Health-Related Quality of Life Among Patients After Acute COVID-19 Infection: A Cross-Sectional Study. Inquiry: A Journal of Medical Care Organization, Provision and Financing. 2024;61:469580241246461.

15. Brouwer W, Verbooy K, Hoefman R, van Exel J. Production Losses due to Absenteeism and Presenteeism: The Influence of Compensation Mechanisms and Multiplier Effects. PharmacoEconomics. 2023;41(9):1103-15.

16. Weyand CM, Goronzy JJ. Aging of the Immune System. Mechanisms and Therapeutic Targets. Annals of the American Thoracic Society. 2016;13 Suppl 5(Suppl 5):S422-S8.

17. Arcavi L, Benowitz NL. Cigarette smoking and infection. Archives of Internal Medicine. 2004;164(20):2206-16.

18. Kim J, Lee W, Won J-U, Yoon J-H, Seok H, Kim Y-K, et al. The relationship between occupational noise and vibration exposure and headache/eyestrain, based on the fourth Korean Working Condition Survey (KWCS). PloS One. 2017;12(5):e0177846.

19. Moon J, Ryu B-H. Transmission risks of respiratory infectious diseases in various confined spaces: A meta-analysis for future pandemics. Environmental Research. 2021;202:111679.

20. Keus van de Poll M, Nybergh L, Lornudd C, Hagberg J, Bodin L, Kwak L, et al. Preventing sickness absence among employees with common mental disorders or stress-related symptoms at work: a cluster randomised controlled trial of a problem-solving-based intervention conducted by the Occupational Health Services. Occupational and Environmental Medicine. 2020;77(7):454-61.

21. Mueller C, Sauter M, Barthelme J, Liebers F. The association between manual handling operations and pain in the hands and arms in the context of the 2018 BIBB/BAuA Employment Survey. BMC musculoskeletal disorders. 2021;22(1):644.

22. Boadu EF, Okeke SR, Boadi C, Osei Bonsu E, Addo IY. Work-related respiratory health conditions among construction workers: a systematic narrative review. BMJ open respiratory research. 2023;10(1):e001736.

23. Appelman B, Charlton BT, Goulding RP, Kerkhoff TJ, Breedveld EA, Noort W, et al. Muscle abnormalities worsen after post-exertional malaise in long COVID. Nature Communications. 2024;15(1):17.

24. Åkerstedt T, Wright KP. Sleep Loss and Fatigue in Shift Work and Shift Work Disorder. Sleep medicine clinics. 2009;4(2):257-71.

25. Mendola M, Leoni M, Cozzi Y, Manzari A, Tonelli F, Metruccio F, et al. Long-term COVID symptoms, work ability and fitness to work in healthcare workers hospitalized for Sars-CoV-2 infection. La Medicina Del Lavoro. 2022;113(5):e2022040.

26. Bosma AR, Boot CRL, Snippen NC, Schaafsma FG, Anema JR. Supporting employees with chronic conditions to stay at work: perspectives of occupational health professionals and organizational representatives. BMC Public Health. 2021;21(1):592.

27. Cunningham TR, Guerin RJ, Ferguson J, Cavallari J. Work-related fatigue: A hazard for workers experiencing disproportionate occupational risks. American Journal of Industrial Medicine. 2022;65(11):913-25.

28. Jung S, Pak S, Lee K, Kang C. Classification of Human Failure in Chemical Plants: Case Study of Various Types of Chemical Accidents in South Korea from 2010 to 2017. International Journal of Environmental Research and Public Health. 2021;18(21):11216.

29. Jansen J, Snippen N, Koning P, Boot C, van Ooijen R, Brouwer S. Discrepancies between workers with disabilities and their supervisors in reported work accommodations and associations with return to work. BMC Public Health. 2023;23(1):525.

Click here to access/download
Supporting Information
Appendix 1.docx

Click here to access/download
Supporting Information
Appendix 2.docx

Click here to access/download
Supporting Information
Appendix 3.docx

Click here to access/download
Supporting Information
Appendix 4.docx

Click here to access/download
Supporting Information
Appendix 5.docx
