# Supplementary material for: Prevalence and risk factors of adverse work outcomes among manufacturing workers with Long COVID in Malaysia – A nationwide study
Source: PLoS One. 2026 Sep 11;21(9):e0357125. doi: 10.1371/journal.pone.0357125 (PMC13567834; doi:10.1371/journal.pone.0357125)
Supplement: Supplementary file 3 [file pone.0357125.s009.pdf]

Manuscript title : Prevalence and risk factors of adverse work outcomes among manufacturing workers with Long COVID in Malaysia – a nationwide study

Comments/suggestions to authors for further improvement:

<table>
<tr><th>No.</th><th>Section in manuscript</th><th>Comments/suggestions</th></tr>
<tr><td>1.</td><td>Abstract</td><td>Introduction:
▪ The amendment has been acknowledged and significantly improved.
▪ I would, however, recommend using the phrase ‘increasing prevalence’ instead of ‘high prevalence’, suggesting that the actual prevalence remains uncertain.

Results
▪ The amendment has been acknowledged and significantly improved.
▪ I do, however, have a minor suggestion for you to consider using the phrase ‘in contrast’ or ‘conversely’ instead of ‘meanwhile’. This would create a distinct shift in tone in the results, specifically from risk factors to protective factors.</td></tr>
<tr><td>3.</td><td>Introduction</td><td>I only have one minor suggestion for further improvement of this section:
▪ Para 1, Line 81-83: To cite the reference used.</td></tr>
<tr><td>4.</td><td>Methods</td><td>All amendments have been acknowledged and significantly improved. A few minor technical suggestions to further improve the manuscript.
▪ Line 165: To prevent referencing tables or figures with the term ‘below’, since the figure might appear on the following page, it is adequate to refer to the tables or figures by stating, for example, ‘as illustrated in Figure 1’</td></tr>
<tr><td>5.</td><td>Results</td><td>Please kindly refer to my previous suggestions of the original manuscript which were not included in the ‘Response to Reviewers’ section. The suggestions are related to results on background data, prevalence of adverse work outcomes and risk factors identified.</td></tr>
</table>

Review completed on 6 May 2026
