# Supplementary material for: Prevalence and risk factors of adverse work outcomes among manufacturing workers with Long COVID in Malaysia – A nationwide study
Source: PLoS One. 2026 Sep 11;21(9):e0357125. doi: 10.1371/journal.pone.0357125 (PMC13567834; doi:10.1371/journal.pone.0357125)
Supplement: Supplementary file 4 [file pone.0357125.s010.pdf]

**Responses to Reviewers**

We would like to thank the reviewer for further evaluation of our manuscript and for the constructive and insightful comments provided. We have carefully considered all suggestions and have revised the manuscript accordingly to improve its clarity, methodological transparency, and overall robustness, as follows.

**Reviewer 1**

1. Abstract: The amendment has been acknowledged and significantly improved. I would, however, recommend using the phrase ‘increasing prevalence’ instead of ‘high prevalence’, suggesting that the actual prevalence remains uncertain.

   **RESPONSE:** We totally agree that the terminology of “increasing prevalence” is more appropriate that “high prevalence”. As such, the terminology has been revised accordingly.

2. Abstract: The amendment has been acknowledged and significantly improved. I do, however, have a minor suggestion for you to consider using the phrase ‘in contrast’ or ‘conversely’ instead of ‘meanwhile’. This would create a distinct shift in tone in the results, specifically from risk factors to protective factors.

   **RESPONSE:** Indeed, “conversely” provide more linguistic clarity than “meanwhile”. This terminology has been revised as suggested.

3. Introduction: Para 1, Line 81-83: To cite the reference used.

   **RESPONSE:** The statement in Line 81-83 has been added with a proper reference from the OECD, as follows: “Gonzalez AE, Suzuki E. OECD Health Working Paper No. 167: The impacts of long COVID across OECD countries. United Kingdom: Organisation for Economic Co-operation and Development (OECD); 2024”.

4. Methods: Line 165: To prevent referencing tables or figures with the term ‘below’, since the figure might appear on the following page, it is adequate to refer to the tables or figures by stating, for example, “as illustrated in Figure 1”.

   **RESPONSE:** Thank you for the reminder. The use of terminology such as “below” has been removed.

5. Background data: Consider presenting the geographic locations of the companies involved in this study. This would help readers better assess the representativeness of the sample and the generalisability of the findings to Malaysian manufacturing companies.

   **RESPONSE:** The geographical location of the included companies has been presented in Line 126-127: “This resulted in three, four, three, six, and two companies participated across East Coast, Northern, Southern, Central, and East Malaysia”.

6. Although it is included in Table 2 (currently referred to as Table 1), I suggest that the socio-clinical data of the participants be presented separately.

**RESPONSE:** The socio-clinical data of the participants has been presented separately in Table 2.

7. Prevalence of adverse work outcomes: The prevalence is not explicitly mentioned. It remains uncertain whether the WRQoL results reflect the prevalence of negative work outcomes. In the subsequent sections (Discussion and Conclusion), different narratives were provided i.e., poor vs high WRQoL. Consider rephrasing.

   **RESPONSE:** Apology for the confusion and inconsistency. Indeed, the prevalence of poor WRQoL was one of the adverse work outcomes, along with sickness absenteeism, sickness presenteeism, and safety issues at work (Table 1). To ensure better clarity, the sentence has been rephrased as such: "In terms of the prevalence of adverse work outcomes, almost a quarter of the workers with Long COVID (24.7%) exhibited poor WRQoL. Sickness absenteeism and sickness presenteeism were reported by 12.8% and 42.4% of workers with Long COVID, respectively. Only a small number of workers (6.3%) reported experiencing safety issues at work".

8. Risk factors: In my opinion, Table 3-7 solely present the data in percentage rather than factors that are determined by statistical analysis. I would strongly suggest renaming the table titles to prevent any potential misunderstanding among readers. Consider indicating the type of statistical analysis used, as footnote, in all tables provided as Appendix 1-4.

   **RESPONSE:** Thank you for the suggestion. To prevention misunderstanding to the tables, the terminology of "description of" was added to the title of the relevant tables, to illustrate that the tables are purely descriptive in nature, without inferential analysis. Meanwhile, all appendices were revised with the statistical analyses (i.e., simple and multiple logistic regressions) being stated as footnotes.

**Reviewer 2**

1. Limitations due to recruitment, selection bias (convenience sampling); in addition to the risk of recall bias, potential misclassification bias should be discussed (if the questionnaire-based algorithm lack clinical validation). Revise wording that may imply overinterpretation or strong claims.

   **RESPONSE:** Indeed, we agree that these limitations are very relevant. A detailed discussion on these limitations has been added, as follows: "the use of convenience sampling may have introduced selection bias, as participants who were more willing or available to participate could differ systematically from those who did not participate. Consequently, the findings may not be fully generalisable to all Malaysian manufacturing workers with Long COVID. Thirdly, the study relied on self-reported information regarding previous COVID-19 infection, Long COVID symptoms, workplace exposures, and adverse work outcomes, which may be subject to recall bias. Participants may have underreported or overreported their symptoms and occupational experiences, particularly for events occurring several months prior to the survey. In addition, there is a possibility of misclassification bias, particularly in the identification of Long COVID status and workplace exposures, as no objective clinical verification or workplace exposure measurements were performed. Some participants may have

inaccurately classified their symptoms or exposures, potentially affecting the observed associations. Nevertheless, the study provides important preliminary evidence on the occupational impacts of Long COVID among manufacturing workers and highlights the need for further prospective studies using probability sampling methods and objective assessments”.

2. Proofreading to improve language and grammar throughout, ensure consistency of terminology and review clarity of table titles, labels/captions.

   **RESPONSE:** The revised manuscript has been subjected to proofreading to improve on language and grammar use, consistency of terminology and clarity of table titles, labels/captions.
