# Supplementary material for: Prevalence and risk factors of adverse work outcomes among manufacturing workers with Long COVID in Malaysia – A nationwide study
Source: PLoS One. 2026 Sep 11;21(9):e0357125. doi: 10.1371/journal.pone.0357125 (PMC13567834; doi:10.1371/journal.pone.0357125)
Supplement: Supplementary file 5 [file pone.0357125.s011.pdf]

PLOS One

Prevalence and risk factors of adverse work outcomes among manufacturing workers with Long COVID in Malaysia – a nationwide study

--Manuscript Draft--

**Manuscript Number:**	PONE-D-25-68765R2
**Article Type:**	Research Article
**Full Title:**	Prevalence and risk factors of adverse work outcomes among manufacturing workers with Long COVID in Malaysia – a nationwide study
**Short Title:**	Adverse work outcomes among manufacturing workers with Long COVID
**Corresponding Author:**	Sheng Qian Yew<br>National University of Malaysia: Universiti Kebangsaan Malaysia<br>Serdang, Selangor MALAYSIA
**Keywords:**	Work impact; post covid; manufacturer; employer; employee
**Abstract:**	Introduction: The high prevalence and multiorgan involvement of Long COVID among the general population has raised concerns about the possible long-term effect of this condition among industrial workers. Therefore, this study seeks to determine the prevalence and risk factors of adverse work outcomes among Malaysian manufacturing workers affected by Long COVID.<br><br>Materials and Methods: A cross-sectional survey was conducted using the Long COVID Questionnaire, Work-Related Quality of Life Scale, and the Work Productivity and Activity Impairment Questionnaire, which were physically distributed to previously-COVID-19 infected participants via convenient sampling. Simple logistic regression and multiple logistic regression were used to determine the association between the various risk factors and adverse work outcomes.<br><br>Results: Among the 797 participants, a majority (75.3%) of the workers with Long COVID demonstrates high WRQoL despite their illness. A total of 12.8% and 42.4% of workers with Long COVID had issues of sickness absenteeism and sickness presenteeism, respectively. Only 6.3% of the Long COVID workers had safety issues at work. Socio-clinical factors [i.e., increased in age (aOR=1.02) and smoking (aOR=1.38)], Long COVID factors [i.e., increased number of Long COVID symptoms (aOR=1.15)], work factors [i.e., engaged in shift work (aOR=1.25), handling of chemical hazardous to health (aOR=2.51), exposure to confined space entry (aOR=1.60), exposure to excessive noise (aOR=1.55), and manual handling (aOR=1.33)], organisational factors [i.e., absence of sickness absence policy (aOR=2.77)] were significant risk factors of adverse work outcomes among workers with Long COVID. Meanwhile, personal factor [i.e., informing employers about the Long COVID symptom (aOR=0.32-0.58)] had protective effect on adverse work outcomes.<br><br>Conclusions: The impact of Long COVID on WRQoL, sickness absenteeism, sickness presenteeism, safety issues at work, as well as the multifactorial nature of adverse work outcomes highlights the need for targeted workplace interventions, supportive policies, early detection, and inclusive occupational health frameworks at the workplace.
**Order of Authors:**	Hanizah Mohd Yusoff
	Sheng Qian Yew
	Azmawati Mohd Nawi
	Noorlaili Mohd Tohit
	Nor Ba'yah Abdul Kadir
	Anita Abd Rahman
	Rahmat Dapari
	Ohnmar Htwe

Powered by Editorial Manager® and ProduXion Manager® from Aries Systems Corporation

	Muhamad Hazizi Muhamad Hasani
	Sivarajan Ramasamy
	Zuraida Mohamad
	Radzi Abdul Rashid
	Mazrura Sahani
	Azuraliza Abu Bakar
	Ida Rosnita Ismail
	Muhamad Ariff Muhamad Noordin
	Fazlin Mohd
	Amalia Sakina
	Kamilah Amnani
**Opposed Reviewers:**	
**Response to Reviewers:**	We would like to thank the reviewer for the careful evaluation of our manuscript and for the constructive and insightful comments provided. We have carefully considered all suggestions and have revised the manuscript accordingly to improve its clarity, methodological transparency, and overall robustness, as follows.<br><br>RESPONSES TO EDITOR<br><br>1.Please ensure that your manuscript meets PLOS ONE's style requirements, including those for file naming.<br><br>RESPONSE: The manuscript has been revised accordingly based on PLOS ONE's style requirement.<br><br>2.Thank you for stating the following financial disclosure: "This work was supported by the National Institute of Occupational Safety and Health (NIOSH) Malaysia [grant number: UKMP-S230424] to [HMY]. Please state what role the funders took in the study. If the funders had no role, please state: "The funders had no role in study design, data collection and analysis, decision to publish, or preparation of the manuscript". If this statement is not correct you must amend it as needed. Please include this amended Role of Funder statement in your cover letter; we will change the online submission form on your behalf.<br><br>RESPONSE: Thank you for the reminded. Indeed, the funder (NIOSH Malaysia) had no role in study design, data collection and analysis, decision to publish, or preparation of the manuscript. This statement has been added to the manuscript and the cover letter.<br><br>3.We note that you have indicated that there are restrictions to data sharing for this study. For studies involving human research participant data or other sensitive data, we encourage authors to share de-identified or anonymized data. However, when data cannot be publicly shared for ethical reasons, we allow authors to make their data sets available upon request. For information on unacceptable data access restrictions, please see http://journals.plos.org/plosone/s/data-availability#loc-unacceptable-data-access-restrictions. Before we proceed with your manuscript, please address the following prompts:<br><br>•If there are ethical or legal restrictions on sharing a de-identified data set, please explain them in detail (e.g., data contain potentially identifying or sensitive patient information, data are owned by a third-party organization, etc.) and who has imposed them (e.g., a Research Ethics Committee or Institutional Review Board, etc.). Please also provide contact information for a data access committee, ethics committee, or other institutional body to which data requests may be sent.<br>•If there are no restrictions, please upload the minimal anonymized data set necessary to replicate your study findings to a stable, public repository and provide us with the relevant URLs, DOIs, or accession numbers. Please see

http://www.bmj.com/content/340/bmj.c181.long for guidelines on how to de-identify and prepare clinical data for publication. For a list of recommended repositories, please see https://journals.plos.org/plosone/s/recommended-repositories. You also have the option of uploading the data as Supporting Information files, but we would recommend depositing data directly to a data repository if possible.

RESPONSE: Regrettably, the Long COVID dataset cannot be shared publicly primarily due to concerns around patient privacy, legal regulations, and ethical approvals. Even when personal identifiers are removed, individuals may still be re-identified from combined data (e.g., working department, position at company, work schedule, etc), especially in workplace with small number of workers. Additionally, the Personal Data Protection Act 2010 restricts the sharing of sensitive health information. Furthermore, the ethics committee limits data use to research purposes, and public sharing may violate the conditions under which participants originally consented. This explanation is provided at the availability of data and materials section.

4.Please include captions for your Supporting Information files at the end of your manuscript, and update any in-text citations to match accordingly. Please see our Supporting Information guidelines for more information: http://journals.plos.org/plosone/s/supporting-information.

RESPONSE: The captions of all supporting information files has been added to the end of the manuscript, and the in-text citations have been revised accordingly.

RESPONSES TO REVIEWER 1

1.Introduction (abstract): The two sentences seem to contradict each other. The first statement mentions a high prevalence of Long COVID, while the second indicates that the study's goal was to determine the prevalence. Consider rephrasing. Consider highlighting the knowledge gap and problem statement in this section.

RESPONSE: Thank you for highlighting the blind spot. To better illustrate the research gap and the justifications of the present study, the introduction of the abstract has been revised to: "The high prevalence and multiorgan involvement of Long COVID among the general population has raised concerns about the possible long-term effect of this condition among industrial workers". It was the observation of the high prevalence of Long COVID among the general population that inspired the authors to conduct a study specifically among the manufacturing workers.

2.Materials and methods (abstract): The instruments used are mentioned but the methods used to distribute the instruments are not. Please state accordingly. Consider moving the total number of respondents (i.e., 797) to the results section. Consider adding the inclusion and exclusion criteria of participants. Line 52 – to correct the spelling of the word 'utilised'.

RESPONSE: The methods of distributing the questionnaires was added to the materials and methods section of the abstract. Due to the word limit of the abstract, we could only add a brief description of the inclusion criteria of the participants. The total number of participants has been moved to the result section as advised and spelling error has been corrected.

3.Results (abstract): For better clarity, consider restructuring the first part of the results section. I would suggest starting with the number of respondents, followed by prevalence and so on. Line 59-60: There is a repetition of information i.e., 42.4%. Consider rephrasing. Regarding the reported risk factors, consider reporting key effect sizes (e.g., adjusted odds ratios) with 95% confidence intervals to allow readers to interpret the magnitude of the associations.

RESPONSE: The number of participants has been added. The repetition of 42.4% was removed. The aOR for all associated risk factors were included.

4.Conclusion (abstract): The conclusion mostly repeats the results and is quite general. Consider making it stronger by emphasizing the main implications of the findings for occupational health practices or workplace policies that support workers dealing with

	Long COVID. This would give readers a clearer idea of the importance of the study.<br><br>RESPONSE: Due to the word limits in abstract, we are unable to extensively elaborate on the impact of the study. However, we concisely emphasised the study implications as follows: “The impact of Long COVID on WRQoL, sickness presenteeism and sickness absenteeism, as well as the multifactorial nature of adverse work outcomes highlights the need for targeted workplace interventions, supportive policies, early detection, and inclusive occupational health frameworks at the workplace”.<br><br>5.Study design: I would recommend using the phrase ‘cross-sectional survey’ instead.<br><br>RESPONSE: The authors agree that the phrase “cross-sectional survey” is a better description than “cross-sectional study”. Hence, it has been revised accordingly.<br><br>6.Data collection protocol: The protocol was described but could be further elaborated. Consider including this information for better clarity:<br>•Any method used to select the manufacturing companies<br>•The individual that the researcher reached out to provide the list.<br>•Were similar data collection protocols used at all companies visited.<br>•The number of researchers present at the site (if relevant).<br>•The kind of assistance provided during the data collection e.g., explanation on medical terms such as tachycardia, tinnitus etc.<br><br>Consider incorporating the specific types of demographic data gathered in this section. Furthermore, it would be beneficial to indicate the methods used for data collection, such as whether body weight and height were self-reported or measured.<br><br>RESPONSE: Initially, manufacturing companies listed in the Federation of Malaysian Manufacturers (FMM) directory that met the eligibility criteria were selected using a multistage sampling approach. However, due to a very low response rate (e.g., companies declining participation), the researchers subsequently adopted a convenience sampling method. Under this approach, any company that provided consent to participate from each zone in Malaysia was included. After obtaining informed consent from the company, the human resource personnel were contacted to provide a list of workers (i.e., the sampling frame). A standardised data collection protocol was applied across all participating companies. During data collection, a team of 2–5 researchers/enumerators (previously trained using the standardised protocol) was present at each site to conduct researcher-assisted interviews while administering the questionnaire, including clarifying items and explaining complex medical terminology. Sociodemographic information, including age, gender, BMI (from self-reported weight and height), and smoking status, was collected.<br><br>7.Ethical approval: It is unclear why the study requires MREC approval. To clarify. Consider indicating if any authorisation was provided from the manufacturing companies to visit and conduct data collection.<br><br>RESPONSE: The study applied for MREC ethics approval as some of the participants were healthcare professionals working at the Ministry of Health, Malaysia. Written/electronic consent was also obtained from the respective manufacturing companies prior to study collection. Such clarification has been included to the materials and methods section.<br><br>RESPONSES TO REVIEWER 2<br><br>1.Scope of study: The study implies nationwide coverage (e.g. “entire nation”, “across all states”) but there’s lack in clarity regarding geographical precision; the distribution and number of participating manufacturing companies are not explicitly reported. The sampling strategy and data collection procedures are not sufficiently detailed giving an impression of vague representation.<br><br>RESPONSE: A cross-sectional survey was conducted across Malaysia’s five geographical zones (i.e., East Coast, Northern, Southern, Central, and East Malaysia). Initially, a multistage sampling strategy was planned for the selection of manufacturing companies, involving cluster sampling within each geographical zone based on the

total number of companies, followed by proportionate stratified sampling to select workers from the states within each zone. However, due to a very low response rate (e.g., companies declining participation), the researchers opted to use a convenience sampling method instead. This resulted in three, four, three, six, and two companies participated across East Coast, Northern, Southern, Central, and East Malaysia, respectively. Under this approach, any company that provided consent to participate from each zone in Malaysia was included. This was followed by a universal sampling to select workers from each factory.

2.Enrolment criteria: The inclusion and exclusion criteria set may not be sufficiently comprehensive and leaving gaps in ruling out confounding symptoms or pre-existing conditions that could overlap with or mimic Long COVID. More explicit criteria are necessary to strengthen internal validity and reduce misclassification bias.

RESPONSE: We acknowledge that the initial inclusion and exclusion criteria may not have fully accounted for potential confounding symptoms or pre-existing conditions that could overlap with or mimic Long COVID, such as bronchial asthma and chronic obstructive pulmonary disease. To address this, several measures were incorporated into the study design and data collection process. Firstly, information on pre-existing comorbidities (e.g., respiratory, cardiovascular, and metabolic conditions) was collected and participants included in the multivariate analysis to allow adjustment for potential confounders (as reported in appendix). Secondly, participants were required to have a confirmed diagnosis of COVID-19 within a defined time frame (i.e., fully fulling the definition of WHO), to ensure temporal relevance to Long COVID assessment. Thirdly, symptoms were assessed in relation to their onset following COVID-19 infection, which helped differentiate new or persistent symptoms from pre-existing conditions. Such discussion has been added to the discussion section.

3.Survey instruments: The use of established, validated instruments to assess outcomes is a strength and contributes to methodological rigour. However, the development and application of the Long COVID instrument are not sufficiently explained. Reporting of “prevalence” of symptoms and adverse outcomes only accounted for workers alleged LC symptoms. Reporting of subjective parameters remains vague, raising analytical and interpretative concerns regarding measurement validity and comparability.

RESPONSE: Our apology for the blind spot. The development and validation process of the Long COVID questionnaire has been reported in our previously published study protocol, available at: Yusoff HM, Mohamed NC, Yew SQ, Nawi AM, Rashidah Ismail OH, Tohit NM, et al. Prevalence and risk factors of long covid and its associated adverse work outcomes among workers in the manufacturing sector in Malaysia – a mixed-methods study protocol. J Health Popul Nutr. 2024 Nov 25;43(1):194. doi:10.1186/s41043-024-00696-z. Meanwhile, the psychometric properties of the Long COVID questionnaire is still in press. This clarification has been added to the materials and method section.

4.Study design limitations and interpretation of findings: The limitations inherent to the cross-sectional design are not adequately addressed. Greater transparency is needed regarding the structure and content of the survey tools, particularly in relation to several paradoxical or counterintuitive findings (e.g. high WRQoL despite symptom burden), which warrant further methodological and analytical clarification.

RESPONSE: To enhance transparency regarding the structure and content of the survey tools, the development and validation process of the Long COVID questionnaire is described in the study protocol (reference added to the manuscript). However, the questionnaire cannot be made publicly available as it is copyrighted by the sponsor (NIOSH Malaysia). With regard to the seemingly paradoxical finding of relatively high WRQoL despite the presence of Long COVID symptoms, several explanations are possible. Firstly, sufficient coping strategies among workers may mitigate the perceived impact of persistent symptoms on WRQoL. For instance, work-focused rehabilitation, multidisciplinary inpatient and outpatient rehabilitation, psychoeducation, pacing, and breathing strategies, have shown to shift focus from symptom monitoring to optimising functional outcomes. These strategies allowed workers affected by Long COVID to maintain good WRQoL. Secondly, many participants may have experienced mild or

Powered by Editorial Manager® and ProduXion Manager® from Aries Systems Corporation

	manageable symptoms (as most participants only experience one symptom) that did not substantially impair their work functioning, as observed in the low proportion of workers with sickness absenteeism, sickness presenteeism, and safety issues at work. These points have now been discussed in the revised discussion section to provide clearer interpretation of the findings.<br><br>5.Conceptual framework and conclusions: The scope of the study and its underlying conceptual framework are not clearly articulated. Several claims and conclusions may require revision or qualification in view of the study design and sampling approach.<br><br>RESPONSE: The study is based on the premise that Long COVID workers may experience adverse work outcomes, which is associated with socio-clinical factors, work factors, Long COVID factors, organisational factors, and personal factors. A conceptual framework (Figure 1) has been added to the materials and methods section explicitly to improve clarity. We also agree that several conclusions required qualification in light of the cross-sectional design and the use of convenience sampling. Accordingly, the conclusions have been revised to avoid causal language and to emphasise associations rather than causal relationships.<br><br>6.Language and presentation: Refinement of language and expression is recommended for reporting and scientific writing. Improvements in clarity, coherence, and grammatical precision would enhance the overall readability of the manuscript.<br><br>RESPONSE: Thank you for the recommendation. Further attention has been given to ensure the clarity, coherence, and grammatical precision of the manuscript.
**Additional Information:**	
**Question**	**Response**
**Financial Disclosure**<br><br>Enter a financial disclosure statement that describes the sources of funding for the work included in this submission. Review the submission guidelines for detailed requirements. View published research articles from *PLOS ONE* for specific examples.<br><br>This statement is required for submission and **will appear in the published article** if the submission is accepted. Please make sure it is accurate.<br><br>**Funded studies**<br>Enter a statement with the following details:<br>• Initials of the authors who received each award<br>• Grant numbers awarded to each author<br>• The full name of each funder<br>• URL of each funder website<br>• Did the sponsors or funders play any role in the study design, data collection and analysis, decision to publish, or preparation of the manuscript?	Yes

Did you receive funding for this work?	
Please add funding details.<br>as follow-up to "**Financial Disclosure**<br><br>Enter a financial disclosure statement that describes the sources of funding for the work included in this submission. Review the submission guidelines for detailed requirements. View published research articles from *PLOS ONE* for specific examples.<br><br>This statement is required for submission and **will appear in the published article** if the submission is accepted. Please make sure it is accurate.<br><br>**Funded studies**<br>Enter a statement with the following details:<br>• Initials of the authors who received each award<br>• Grant numbers awarded to each author<br>• The full name of each funder<br>• URL of each funder website<br>• Did the sponsors or funders play any role in the study design, data collection and analysis, decision to publish, or preparation of the manuscript?<br><br>Did you receive funding for this work?"	This work was supported by the National Institute of Occupational Safety and Health (NIOSH) Malaysia [grant number: UKMP-S230424] to [HMY].<br>The funder (NIOSH Malaysia) had no role in study design, data collection and analysis, decision to publish, or preparation of the manuscript.
Please select the country of your main research funder (please select carefully as in some cases this is used in fee calculation).<br>as follow-up to "**Financial Disclosure**<br><br>Enter a financial disclosure statement that describes the sources of funding for the work included in this submission. Review the submission guidelines for detailed requirements. View published research articles from *PLOS ONE* for specific examples.<br><br>This statement is required for submission and **will appear in the published article** if	MALAYSIA - MY

the submission is accepted. Please make sure it is accurate.<br><br>**Funded studies**<br>Enter a statement with the following details:<br>• Initials of the authors who received each award<br>• Grant numbers awarded to each author<br>• The full name of each funder<br>• URL of each funder website<br>• Did the sponsors or funders play any role in the study design, data collection and analysis, decision to publish, or preparation of the manuscript?<br><br>Did you receive funding for this work?"	
**Competing Interests**<br><br>Use the instructions below to enter a competing interest statement for this submission. On behalf of all authors, disclose any competing interests that could be perceived to bias this work—acknowledging all financial support and any other relevant financial or non-financial competing interests.<br><br>This statement is required for submission and **will appear in the published article** if the submission is accepted. Please make sure it is accurate and that any funding sources listed in your Funding Information later in the submission form are also declared in your Financial Disclosure statement.<br><br>View published research articles from *PLOS ONE* for specific examples.	The authors declare that they have no competing interests.

**NO authors have competing interests**<br><br>Enter: *The authors have declared that no competing interests exist.*<br><br>**Authors with competing interests**<br><br>Enter competing interest details beginning with this statement:<br><br>*I have read the journal's policy and the authors of this manuscript have the following competing interests: [insert competing interests here]*<br><br>* typeset	
**Data Availability**<br><br>Authors are required to make all data underlying the findings described fully available, without restriction, and from the time of publication. PLOS allows rare exceptions to address legal and ethical concerns. See the PLOS Data Policy and FAQ for detailed information.<br><br>A Data Availability Statement describing where the data can be found is required at submission. Your answers to this question constitute the Data Availability Statement and **will be published in the article**, if accepted.<br><br>**Important:** Stating 'data available on request from the author' is not sufficient. If your data are only available upon request, select 'No' for the first question and explain your exceptional situation in the text box.<br><br>Do the authors confirm that all data underlying the findings described in their manuscript are fully available without restriction?	No - some restrictions will apply
Additional data availability information:	
**Ethics Statement**<br><br>Enter an ethics statement for this submission. This statement is required if	Ethical approval for this study was obtained from the Research Ethics Committee of the National University of Malaysia (JEP-2023-607) and the Medical Research and Ethics Committee (MREC) of the Ministry of Health Malaysia (NMRR ID-23-03310-H3E). All participants provided written consents prior to their participations.

the study involved:<br><br>• Human participants<br>• Human specimens or tissue<br>• Vertebrate animals or cephalopods<br>• Vertebrate embryos or tissues<br>• Field research<br><br>Write "N/A" if the submission does not require an ethics statement.<br><br>General guidance is provided below. Consult the submission guidelines for detailed instructions. **Make sure that all information entered here is included in the Methods section of the manuscript.**	

**Format for specific study types**<br><br>**Human Subject Research (involving human participants and/or tissue)**<br>• Give the name of the institutional review board or ethics committee that approved the study<br>• Include the approval number and/or a statement indicating approval of this research<br>• Indicate the form of consent obtained (written/oral) or the reason that consent was not obtained (e.g. the data were analyzed anonymously)<br><br>**Animal Research (involving vertebrate animals, embryos or tissues)**<br>• Provide the name of the Institutional Animal Care and Use Committee (IACUC) or other relevant ethics board that reviewed the study protocol, and indicate whether they approved this research or granted a formal waiver of ethical approval<br>• Include an approval number if one was obtained<br>• If the study involved *non-human primates*, add *additional details* about animal welfare and steps taken to ameliorate suffering<br>• If anesthesia, euthanasia, or any kind of animal sacrifice is part of the study, include briefly which substances and/or methods were applied<br><br>**Field Research**<br><br>Include the following details if this study involves the collection of plant, animal, or other materials from a natural setting:<br>• Field permit number<br>• Name of the institution or relevant body that granted permission	
**Data Availability**<br><br>Before publication, Authors are required to make fully available and without restriction all data underlying their findings. Sharing the data underlying your findings increases the visibility, credibility, and impact of your work, while supporting transparency and future scientific discovery. Please see our PLOS Data	Data cannot be shared publicly because it consists of current and past medical history of the workers, which may affect their employment. Data are available from the Research Ethics Committee of the National University of Malaysia (contact via +603-9145 5046 / +603-9145 5048) for researchers who meet the criteria for access to confidential data.

Powered by Editorial Manager® and ProduXion Manager® from Aries Systems Corporation

Policy page for detailed information on this policy.<br><br>A **Data Availability Statement**, detailing where the data can be accessed, is required at first submission. Insert your Data Availability Statement in the box below.<br><br>Please see the data reporting section of our submission guidelines for instructions on what you need to include in your Data Availability Statement. Examples of acceptable Data Availability Statements are included here for reference:<br>1. The minimal data set [and accompanying code, where generated] is available at [repository name] via [DOI hyperlink]<br>2. No data was generated by this study. The following existing data sources were used: [dataset name] from [dataset location] available via [web address or DOI hyperlink].<br>3. For Study Protocols: No datasets were generated or analysed during the current study. All relevant data from this study will be made available upon study completion.<br><br>*PLOS allows rare exemptions to address legal and ethical concerns. If you have legal or ethical restrictions, please use the box below to detail these in full sentences for the Journal team to consider.*<br><br>This statement will be typeset if the manuscript is accepted for publication.	

Dear Editor,

I am pleased to submit our revised manuscript entitled "Prevalence and Risk Factors of Adverse Work Outcomes among Manufacturing Workers with Long COVID in Malaysia – A Nationwide Study" for your kind consideration for publication in PLOS ONE.

This study addresses a critical and timely occupational quality of life issue, i.e., the impact of Long COVID on industrial workforce functioning and wellbeing. In particular, we examine how lingering Long COVID symptoms among manufacturing workers in Malaysia compromise work performance and safety, potentially affecting both individual health and broader organisational risk management.

Through a nationwide cross-sectional survey involving 797 manufacturing workers, we utilised validated instruments including the Long COVID Questionnaire, the Work-Related Quality of Life (WRQoL) Scale, and the Work Productivity and Activity Impairment (WPAI) Questionnaire. Our findings demonstrate that while many affected workers strive to maintain productivity, a significant proportion continue to work while unwell (sickness presenteeism), or miss work altogether (absenteeism), both of which carry implications for workplace safety, operational continuity, and employee wellbeing.

Importantly, our multivariate analysis highlights several socio-clinical, occupational, and organisational risk factors associated with adverse work outcomes. A key finding is that workers who disclosed their Long COVID symptoms to their employers were less likely to experience negative work impacts. This emphasise the need for psychologically safe environments, proactive disclosure policies, and inclusive return-to-work practices that prioritise occupational safety and support.

We believe that the insights presented in this study contribute meaningfully to the literature on workplace safety and health in the post-pandemic era. The findings have practical relevance for safety officers, human resource managers, and policymakers seeking to create safer and more resilient work environments in industrial settings.

All authors have approved the manuscript and report no conflicts of interest. This work is original, has not been published elsewhere, and is not under consideration by any other publication. We also like to declare that the funder (NIOSH Malaysia) had no role in study design, data collection and analysis, decision to publish, or preparation of the manuscript

Thank you for your time and consideration. We hope this manuscript aligns with the aims of your journal and look forward to the opportunity to contribute to PLOS ONE.

Warm regards,
Sheng Qian Yew

Manuscript Click here to access/download;Manuscript;Manuscript (Clean).docx

Full Title: Prevalence and risk factors of adverse work outcomes among manufacturing workers with Long COVID in Malaysia – a nationwide study

Short Title: Adverse work outcomes among manufacturing workers with Long COVID

Hanizah Mohd Yusoff [a], Yew Sheng Qian [a]*, Azmawati Mohd Nawi [a], Noorlaili Mohd Tohit [b], Nor Ba'yah Abdul Kadir [c], Anita Abd Rahman [d], Rahmat Dapari [d], Ohnmar Htwe [e], Muhamad Hazizi Muhamad Hasani [f], Sivarajan Ramasamy [f], Zuraida Mohamad [f], Radzi Abdul Rashid [g], Mazrura Sahani [h], Azuraliza Abu Bakar [i], Ida Rosnita Ismail [j], Muhamad Ariff Muhamad Noordin [k], Fazlin Mohd [l], Amalia Sakina [l], Kamilah Amnani [l]

[a] Department of Public Health Medicine, Faculty of Medicine, Universiti Kebangsaan Malaysia, Bandar Tun Razak, Cheras, Kuala Lumpur, Malaysia.

[b] Department of Family Medicine, Faculty of Medicine, Universiti Kebangsaan Malaysia, Bandar Tun Razak, Cheras, Kuala Lumpur, Malaysia.

[c] Faculty of Social Sciences and Humanitarian, Universiti Kebangsaan Malaysia, Bangi, Selangor, Malaysia.

[d] Faculty of Medicine and Health Science, Universiti Putra Malaysia, UPM Serdang, Selangor, Malaysia.

[e] Faculty of Medicine, Universiti Sultan Zainal Abidin Medical, Kuala Terengganu, Terengganu, Malaysia.

[f] Negeri Sembilan State Health Department, Seremban, Negeri Sembilan, Malaysia.

[g] OSH ProCare Consultant and Services, G&C Township, Seremban, Negeri Sembilan, Malaysia.

[h] Centre of Health and Applied Sciences, Faculty of Health Sciences, Universiti Kebangsaan Malaysia, Kuala Lumpur, Malaysia.

[i] Faculty of Information Science and Technology, Universiti Kebangsaan Malaysia, UKM, Bangi, Selangor, Malaysia.

[j] Graduate School of Business, Universiti Kebangsaan Malaysia, Bangi, Selangor, Malaysia.

[k] Research Management Center, National Institute of Occupational Safety and Health, Bandar Baru Bangi, Selangor, Malaysia.

[l] UKM Pakarunding Sdn Bhd, Level 3, Universiti Kebangsaan Malaysia, Bangi, Selangor, Malaysia.

Correspondence:

Sheng Qian Yew (SQY)

Email: shengqian@ukm.edu.my

Tel: 03-91458792

Abstract

Introduction: The increasing prevalence and multiorgan involvement of Long COVID among the general population has raised concerns about the possible long-term effect of this condition among industrial workers. Therefore, this study seeks to determine the prevalence and risk factors of adverse work outcomes among Malaysian manufacturing workers affected by Long COVID.

Materials and Methods: A cross-sectional survey was conducted using the Long COVID Questionnaire, Work-Related Quality of Life (WRQoL) Scale, and the Work Productivity and

Activity Impairment (WPAI) Questionnaire, which were physically distributed to previously-COVID-19 infected participants via convenient sampling. Simple logistic regression and multiple logistic regression were used to determine the association between the various risk factors and adverse work outcomes.

Results: Among the 797 participants, a quarter (24.7%) of the workers with Long COVID demonstrated poor WRQoL. A total of 12.8% and 42.4% of workers with Long COVID had issues of sickness absenteeism and sickness presenteeism, respectively. Only 6.3% of the Long COVID workers had safety issues at work. Socio-clinical factors [i.e., older age (aOR=1.02) and smoking (aOR=1.38)], Long COVID factors [i.e., increased number of Long COVID symptoms (aOR=1.15)], work factors [i.e., engaged in shift work (aOR=1.25), handling of chemicals hazardous to health (aOR=2.51), exposure to confined space entry (aOR=1.60), exposure to excessive noise (aOR=1.55), and manual handling (aOR=1.33)], organisational factors [i.e., absence of sickness absence policy (aOR=2.77)] were significant risk factors of adverse work outcomes among workers with Long COVID. Conversely, personal factors [i.e., informing employers about the Long COVID symptom (aOR=0.32-0.58)] had a protective effect on adverse work outcomes.

Conclusions: The impact of Long COVID on WRQoL, sickness absenteeism, sickness presenteeism, safety issues at work, as well as the multifactorial nature of adverse work outcomes highlights the need for targeted workplace interventions, supportive policies, early detection, and inclusive occupational health frameworks at the workplace.

Keywords: Work impact, post-COVID, manufacturer, employer, employee

1. Introduction

The manufacturing sector is a key contributor to Malaysia's economy, accounting for the second-largest share of the country's gross domestic product (GDP) and an annual contribution exceeding RM300 billion (1). Furthermore, it provides employment for approximately 2.33 million individuals as of November 2022 (2). The industry's reliance on skilled labour is crucial for its efficient operation, making the retention of talented, skilled, and experienced workers essential for enhancing overall performance, quality, efficiency, effectiveness, and productivity within the sector (3). Consequently, maintaining a healthy and productive workforce is vital. Unfortunately, the high number of COVID-19 clusters among manufacturing workers during the pandemic, along with the subsequent high prevalence of Long COVID, has raised concerns regarding the long-term sustainability of this sector (4).

The World Health Organization (WHO) defines Long COVID as a condition that develops in individuals with a prior SARS-CoV-2 infection, typically occurring three months after the initial onset of COVID-19 (5). Additionally, the symptoms must persist for at least two months and cannot be attributed to any other underlying condition. Generally, Long COVID presents with a wide range of symptoms, which are thought to result from a pathology similar to that of acute COVID-19. Some of the most commonly reported symptoms include fatigue, shortness of breath, and cognitive impairment (6).

Given its reported impacts on multiple organ systems among the general population, Long COVID could possibly affect daily functioning, including workplace activities. For instance, workers experiencing Long COVID may face various negative work-related consequences, such as decreased productivity and performance, absenteeism, presenteeism, a higher risk of occupational injuries, and a reduced work-related quality of life (7, 8). Furthermore, workers requiring prolonged medical leave for recovery, ongoing treatment, or those unable to return to work may contribute to increased operational expenses for employers. It is also worth mentioning that the extent of these adverse work outcomes may vary depending on several factors, including the availability of workplace adjustment, company policies on sick leave, and return-to-work strategies implemented within the organisation.

While previous research has documented various negative effects of Long COVID, most studies have primarily focused on the general population (9, 10). Consequently, there is limited knowledge regarding the specific impact of Long COVID on work-related outcomes among manufacturing workers. An updated study on the burden of Long COVID within this sector would help workers, employers, policymakers, and other stakeholders better understand the scale of the issue affecting the manufacturing industry. On the basis of these research gaps and this rationale, this study aims to assess the prevalence and risk factors associated with adverse work outcomes among manufacturing workers in Malaysia who are affected by Long COVID.

2. Materials and Methods

A cross-sectional survey was conducted from April 2024 to October 2024 across Malaysia's five geographical zones (i.e., East Coast, Northern, Southern, Central, and East Malaysia). The target population consisted of workers from manufacturing companies listed in the Federation of Malaysian Manufacturers (FMM) directory, with eligibility criteria aligned with those specified in Table 1. Initially, a multistage sampling strategy was planned for the selection of manufacturing companies, involving cluster sampling within each geographical zone based on the total number of companies, followed by proportionate stratified sampling to select workers from the states within each zone. However, due to a very low response rate (e.g., companies declining participation), the researchers opted to use a convenience sampling method instead. Under this approach, any company that provided consent to participate from each zone in Malaysia was included. This resulted in three, four, three, six, and two companies ~~participating in~~ East Coast, Northern, Southern, Central, and East Malaysia, respectively. This was followed by a universal sampling to select workers from each of these companies. Given a precision level of 0.05, a confidence level of 95%, a prevalence of adverse work outcomes of 50.0% (in the absence of data on prevalence of adverse work outcome among workers with Long COVID), and a non-response rate of 50%, the total sample size required nationwide was 768. This sample size estimation was calculated using Kish's formula (11).

Table 1: Inclusion and exclusion criteria for manufacturing workers

Inclusion criteria	Exclusion criteria
1. Workers of the manufacturing company (including electrical, electronics, petroleum,	1. Workers who had resigned or terminated.

chemical, rubber, plastic, non-metallic mineral products, basic metal, fabricated metal, food and beverages, tobacco, wood, furniture, paper products, printing, textile, wearing apparel, leather, footwear, and transport equipment sectors).<br>2. Workers diagnosed with COVID-19 from 1st January 2022 until 30th April 2024.<br>3. Malaysian and legal foreign workers.<br>4. Worked for at least six months prior to the acute phase of COVID-19.<br>5. Able to communicate in English and/or Malay.	2. Temporary workers.<br>3. Pregnant women during data collection or during acute phase of COVID-19.<br>4. Workers who did not consent for participation.

Three previously validated questionnaires, namely the Long COVID Questionnaire, Work-Related Quality of Life (WRQoL) Scale (12), and the Work Productivity and Activity Impairment (WPAI) Questionnaire (13), were utilised to assess the prevalence and risk factors of adverse work outcomes. Permission to use these questionnaires was obtained from their respective developers.

The 67-item Long COVID Questionnaire was used to evaluate both the presence of Long COVID symptoms and the associated adverse work outcomes among manufacturing workers (14). The questionnaire is divided into three sections. Section A comprises seven items designed to collect socio-clinical data, including age, gender, BMI (from self-reported weight and height),

comorbidities, and smoking status. Section B contains 57 items assessing the presence of Long COVID, where a worker is classified as having Long COVID if they respond with "Yes," "Yes," and "No" for parts a, b, and c of each symptom listed, respectively. Lastly, Section C consists of three items aimed at identifying workplace safety concerns. A worker is considered to have safety issues at work if they select "unsure," "affected," or "very much affected" for at least one item in this section.

The 24-item WRQoL Scale was used to assess the perceived quality of life of the manufacturing workers with Long COVID (12). WRQoL was measured through six psychosocial subscales (i.e., general well-being, home-work interface, job-career satisfaction, control at work, working conditions, and stress at work). For score interpretation, items 7, 9, and 19 are reverse-coded. In this study, the total WRQoL score was derived by summing all 24 item scores, with a score of 83 or higher indicating a high quality of work life, while a score below 83 is classified as low WRQoL.

The 6-item, unidimensional WPAI Questionnaire was employed to evaluate sickness absenteeism and presenteeism among manufacturing workers affected by Long COVID (13). A worker is classified as having sickness absenteeism if they missed at least 1% of their assigned work time in the past seven days. Likewise, sickness presenteeism is identified when a worker reports at least a 1% reduction in work performance due to health issues within the same period. The conceptual framework of the study is shown in Figure 1.

Figure 1: The conceptual framework of the study

Data collection involved on-site visits by 2–5 researchers and/or enumerators at each selected manufacturing company. In total, there were 32 researchers and/or enumerators, who were trained using the standardised data collection protocol. This allowed the study to be conducted nationwide over the same period using a standardised protocol. After obtaining informed consent from the company, the human resource personnel were contacted to provide a list of workers (i.e., the sampling frame) who had been diagnosed with and documented as having acute COVID-19. With the cooperation of the management, the selected workers were assembled in a designated room within the management office. Each worker was then screened for eligibility based on the criteria outlined in Table 1. Eligible workers were provided with a briefing on the study objectives, instructions for questionnaire administration, and their rights before providing informed consent to participate. Those who were deemed ineligible or who declined participation were thanked and permitted to return to their work. Prior to commencing the survey, informed consent was obtained from all eligible workers. They were encouraged to complete the questionnaire with the assistance of the researchers and/or enumerators, and sufficient time was allocated to ensure high-quality data collection. The role of the researcher and/or enumerators was to clarify items and explain complex medical terminology in the questionnaire. Workers' anonymity and confidentiality were upheld throughout the study. To minimise disruptions to workplace operations, data collection was conducted in a staggered manner, with only one to two workers completing the questionnaire at a time.

Descriptive analysis was conducted to present the prevalence of adverse work outcomes and risk factors of adverse work outcomes. Simple logistic regression was performed to examine the association between the various risk factors (including socio-clinical, Long COVID, work,

organisational, and personal factors) and adverse work outcomes. Risk factors with a p-value < 0.25 were included in a multiple logistic regression model. Additionally, the moderating effects of organisational and personal factors on Long COVID symptoms were assessed using multiple logistic regression. All statistical analyses were performed using SPSS version 28.

Ethical approval for this study was obtained from the Research Ethics Committee of the National University of Malaysia (JEP-2023-607) and the Medical Research and Ethics Committee (MREC) of the Ministry of Health Malaysia (NMRR ID-23-03310-H3E). Ethical approval was obtained from the Medical Research and Ethics Committee (MREC) as the study included healthcare professionals from the Ministry of Health Malaysia. Participants recruitment began on 15th January 2024 and ended on 16th December 2024. All participants provided written informed consent prior to their participation. In addition, written or electronic consent was secured from the participating manufacturing companies prior to data collection.

3. Results

In the present study, adverse work outcomes included poor WRQoL, work productivity (sickness absenteeism and sickness presenteeism), and safety issues at work (Table 1). In terms of the prevalence of adverse work outcomes, almost a quarter of the workers with Long COVID (24.7%) exhibited poor WRQoL. Sickness absenteeism and sickness presenteeism were reported by 12.8% and 42.4% of workers with Long COVID, respectively. Only a small number of workers (6.3%) reported experiencing safety issues at work.

Table 1: Prevalence of adverse work outcomes among the manufacturing workers with Long COVID

Adverse work outcomes	Workers with LC, n (%) N = 797
**Work-related quality of life (WRQoL)**	
Poor	197 (24.7)
Good	600 (75.3)
**Work productivity (sickness absenteeism)**	
Yes	102 (12.8)
No	695 (87.2)
**Work productivity (sickness presenteeism)**	
Yes	338 (42.4)
No	459 (57.6)
**Safety issues at work**	
Yes	50 (6.3)
No	747 (93.7)

The risk factors of adverse work outcomes investigated in the present study include socio-clinical, Long COVID, work, organisational, and personal factors. The socio-clinical factors of the manufacturing workers with Long COVID were presented in Table 2. The mean age of the workers was 37.4 ± 9.5 years. Most workers were female (54.5%), overweight (37.6%) and did not have comorbidities (64.4%). Among those with co-morbidities, hypertension (13.2%) was the most

common, followed by asthma (7.8%) and diabetes (5.4%). Additionally, the majority of workers were non-smokers (78.2%).

Table 2: Socio-clinical factors of the manufacturing workers with Long COVID

**Individual factors among manufacturing workers**	**Mean (SD)**	**n (%) N = 797**
**Age (years)**	37.4 (9.5)	-
**Gender**		
Male	-	363 (45.5)
Female	-	434 (54.5)
**BMI category (kg/m^2)**		
< 18.5 (underweight)	-	38 (4.8)
18.5 – 24.9 (normal)	-	276 (34.6)
25.0 – 29.9 (overweight)	-	300 (37.6)
> 30 (obese)	-	183 (23.0)
**Comorbidities**		
Total comorbidities (may overlap)	-	284 (35.6)
Hypertension	-	105 (13.2)
Asthma	-	62 (7.8)
Diabetes	-	43 (5.4)
Anxiety/Panic attack	-	16 (2.0)
Heart disease	-	11 (1.4)

Autoimmune disease	-	10 (1.3)
Chronic kidney disease	-	4 (0.5)
Chronic liver disease	-	1 (0.1)
Cancer	-	0 (0.0)
HIV Infection	-	0 (0.0)
Others†	-	94 (11.8)
**Smoking status**		
Non-smokers	-	623 (78.2)
Smokers	-	174 (21.8)

**Range of age = 22 – 63 years old*

†*Others include gastritis, dyslipidaemia, gout, and psoriasis.*

As presented in Table 3, among the 797 manufacturing workers with Long COVID, the most common symptoms for Long COVID were post-exertional malaise (50.9%), followed by fatigue (37.4%), and joint pain (25.6%). Of note, the workers experienced a wide range of symptoms, ranging from one symptom to 19 symptoms, with most experiencing only one symptom (35.3%).

Table 3: Long COVID factors of the manufacturing workers

**Long COVID symptoms**	**n (%)**<br>**N = 797**
Post-exertional malaise	406 (50.9)
Fatigue	298 (37.4)

Joint pain	204 (25.6)
Muscle pain	171 (21.5)
Cough	164 (20.6)
Headache	142 (17.8)
Tingling	140 (17.6)
Cognitive dysfunction	136 (17.1)
Dyspnoea	133 (16.7)
Tachycardia	133 (16.7)
Chest Pain	120 (15.1)
Sleeping disturbance	105 (13.2)
Intermittent fever	93 (11.7)
Dizziness	91 (11.4)
Tinnitus	77 (9.7)
Diarrhoea	55 (6.9)
Altered sensation	39 (4.9)
Abdominal pain	27 (3.4)
Depression	26 (3.3)
**No. of Long COVID Symptom(s) Experienced**	
1	281 (35.3)
2	158 (19.8)
3	109 (13.7)
4	76 (9.5)
5	42 (5.3)

6	38 (4.8)
7	22 (2.8)
8	18 (2.3)
9	17 (2.1)
10	13 (1.6)
11	6 (0.8)
12	5 (0.6)
13	7 (0.9)
14	1 (0.1)
15	2 (0.2)
16	1 (0.1)
17	0 (0.0)
18	1 (0.1)
19	0 (0.0)

Table 4 presents the work factors of the manufacturing workers with Long COVID. Most of the workers interviewed were working in the electrical and electronics subsectors (52.3%) and most of them were working in the operation/supply chain department (84.8%)". Additionally, most of the workers were operators (40.8%) and primarily worked normal office hours (51.9%). In terms of types of hazards at work, the most commonly identified workplace hazards included visual display units (VDU) (61.9%), manual handling (40.8%), and excessive noise (29.5%).

Table 4: Description of work factors of the manufacturing workers with Long COVID

Work factors of the manufacturing workers	n (%) N = 797
**Industrial subsectors**	
Electrical and electronics	417 (52.3)
Transport, vehicle and equipment	163 (20.5)
Rubber	119 (14.9)
Textile, wearing apparel and leather	74 (9.3)
Chemical (including petroleum)	11 (1.4)
Paper, printing, and publishing	10 (1.3)
Food, beverage, and tobacco	3 (0.4)
**Working departments**	
Operations/supply chain	676 (84.8)
Research and development (R&D)	29 (3.6)
Human resource	23 (2.9)
Finance (including accounts)	20 (2.5)
Facilities	17 (2.1)
Sales	8 (1.0)
Procurement	8 (1.0)
Marketing	5 (0.6)
Corporate	3 (0.4)
Others	8 (1.0)
**Positions at company**	

Operator	325 (40.8)
Technician, specialist or permit holder	199 (25.0)
Executive	77 (9.7)
Supervisor	74 (9.3)
Officer	47 (5.9)
Manager	46 (5.8)
Clerk	15 (1.9)
General worker	5 (0.6)
Support services	4 (0.5)
Director	1 (0.1)
Others	4 (0.5)
**Work schedule**	
Normal/office hours	414 (51.9)
Shift including staggered shift	382 (47.9)
Flexible	1 (0.1)
Work from home	0 (0.0)
**Types of hazards at work**	
VDU (visual display unit)/video (computer)	493 (61.9)
Manual handling	325 (40.8)
Excessive noise	235 (29.5)
Handling of hazardous chemicals	206 (25.8)
Electrical work	158 (19.8)
Working in extreme temperatures (hot or cold)	99 (12.4)

Confined space entry	91 (11.4)
Working at heights	60 (7.5)
Lone/isolated work	45 (5.6)
Driving vehicle	35 (4.4)
Working on roofs	33 (4.1)
Construction work	30 (3.8)
Driving lift truck	28 (3.5)
†Others	40 (5.0)

†*Example: Working with sharp objects and risk of falling objects*

Analysis of the organisational factors found that 53.3% of the workers with Long COVID perceived that work adjustment was feasible at their workplaces (Table 5). Furthermore, almost half (49.4%) of the workers with Long COVID perceived that a sickness absence policy was present at their workplace. Only 32.9% of the workers believed that return-to-work (RTW) practices were present at their workplace.

Table 5: Description of organisational factors of manufacturing workers with Long COVID symptoms

Organisational factors of manufacturing workers with Long COVID symptoms	n (%) N = 797	
	**Yes (%)**	**No (%)**
Presence of work adjustment feasibility	425 (53.3)	372 (46.7)

Presence of sickness absence policy at workplace	394 (49.4)	403 (50.6)
Presence of RTW practices at workplace	262 (32.9)	535 (67.1)

In terms of personal factors, most of the workers with Long COVID had a good work attitude (93.9%), despite their condition (Table 6). Among the workers with Long COVID symptoms, most did not inform their employers (89.1%).

Table 6: Description of personal factors of manufacturing workers with Long COVID symptoms

Personal factors of manufacturing workers with Long COVID symptoms	Workers with LC, n (%) N = 797
**Work attitude**	
Good	748 (93.9)
Poor	49 (6.1)
**Informed employer on Long COVID**	
Yes	87 (10.9)
No	710 (89.1)

To investigate the association with WRQoL, a total of 21 risk factors (i.e., socio-clinical, Long COVID, work, organisational, and personal factors with $p < 0.25$ in the simple logistic regression) were modelled using multiple logistic regression (MLR) with a forward method (S1 Table). All assumptions of MLR (i.e., linearity, interaction, multicollinearity, and outliers) were met. Out of

the 21 risk factors, only five risk factors were significantly associated with WRQoL, namely age, smoking status, excessive noise, confined space entry and sickness absence policy at workplace. For workers with Long COVID symptoms, each additional year of age is associated with a 2% increase in the odds of experiencing poor WRQOL (aOR = 1.02, $p < 0.001$). Workers with Long COVID symptoms who smoke have 1.38 times higher odds of suffering from poor WRQOL (aOR = 1.38, $p < 0.001$), compared with those who did not smoke. Workers with Long COVID symptoms who were exposed to excessive noise had 1.55 times higher odds of suffering from poor WRQOL (aOR = 1.55, $p < 0.001$), compared with those who were not exposed. Workers with Long COVID symptoms subjected to confined space entry had 1.60 times higher odds of suffering from poor WRQOL (aOR = 1.60, $p = 0.013$), compared with those who were not exposed. Workers who have experienced the absence of a sickness absence policy at the workplace had 2.77 times higher odds of suffering from poor WRQOL (aOR = 2.77, $p < 0.001$), compared with those who had a sickness absence policy.

To investigate the association with sickness absenteeism, a total of 10 risk factors (i.e., socio-clinical, Long COVID, work, organisational, and personal factors with $p < 0.25$ in the simple logistic regression) were modelled using MLR with a forward method (S2 Table). All assumptions of MLR (i.e., linearity, interaction, multicollinearity, and outliers) were met. Out of the 10 risk factors, only manual handling was significantly associated with sickness absenteeism. Workers with Long COVID symptoms who are exposed to manual handling had 1.33 times higher odds of having sickness absenteeism (aOR = 1.33, $p = 0.037$), compared with those who were not exposed.

To investigate the association with sickness presenteeism, a total of 17 risk factors (i.e., socio-clinical, Long COVID, work, organisational, and personal factors with $p < 0.25$ in the simple logistic regression) were modelled using MLR with a forward method (S3 Table). All assumptions of MLR (i.e., linearity, interaction, multicollinearity, and outliers) were met. Out of the 17 risk factors, only three risk factors were significantly associated with sickness presenteeism, namely work schedule, number of Long COVID symptoms, and informing employers regarding the Long COVID symptoms. Workers with Long COVID symptoms who engaged in shift work had 1.25 times higher odds of having sickness presenteeism (aOR = 1.25, $p = 0.027$), compared with those who engaged in normal working hours. The odds of having sickness presenteeism increased by 1.15 times for every increase in the number of Long COVID symptoms (aOR = 1.15, $p < 0.001$). Informing the employer regarding the Long COVID symptoms has been found to be a protective factor against sickness presenteeism where they had 42% lower odds of having sickness presenteeism (aOR = 0.58, $p = 0.019$), compared with those who did not inform.

To investigate the association with safety issues at work, a total of 26 risk factors (i.e., socio-clinical, Long COVID, work, organisational, and personal factors with $p < 0.25$ in the simple logistic regression) were modelled using MLR with a forward method (S4 Table). All assumptions of MLR (i.e., linearity, interaction, multicollinearity, and outliers) were met. Out of the 26 risk factors, only two risk factors were significantly associated with safety at work, namely handling of chemicals hazardous to health and informing employers regarding Long COVID symptoms. Workers with Long COVID symptoms who handled chemicals hazardous to health had 2.51 times higher odds of having safety issues at work (aOR = 2.51, $p < 0.001$), compared with those who did not handle. Informing the employer regarding their Long COVID symptoms has been found to be

a protective factor against safety issues at work where they had 68% lower odds of having safety issues at work (aOR = 0.32, p = 0.007), compared with those who did not inform.

According to the multiple logistic regression, it was found that none of the organisational factors and personal factors had a moderating effect on the relationship between Long COVID and adverse work outcomes (S5 Table).

4. Discussion

Our findings suggest that a relatively low prevalence (almost a quarter) of workers with Long COVID had poor WRQoL. In fact, a recent study also found that there were no significant differences in the QoL between those with and without Long COVID (15). With regard to the seemingly paradoxical finding of relatively high WRQoL despite the presence of Long COVID symptoms, several explanations are possible. Firstly, sufficient coping strategies among workers may mitigate the perceived impact of persistent symptoms on WRQoL. For instance, work-focused rehabilitation, multidisciplinary inpatient and outpatient rehabilitation, psychoeducation, pacing, and breathing strategies, have been shown to shift focus from symptom monitoring to optimising functional outcomes. These strategies allowed workers affected by Long COVID to maintain good WRQoL. Secondly, many participants may have experienced mild or manageable symptoms (as most participants experienced only one symptom) that did not substantially impair their work functioning, as observed in the low proportion of workers with sickness absenteeism, sickness presenteeism, and safety issues at work. Hence, it can be concluded that although Long COVID

symptoms could affect multiple body systems, these symptoms are manageable and have little impact on WRQoL.

It is noteworthy that although only 12.8% of the workers with Long COVID absent from their work due to the symptoms, a relatively large proportion of workers (42.4%) continued to work despite having Long COVID symptoms. This may lead to reduced productivity, prolonged recovery times, and potential worsening of symptoms due to the lack of rest and proper medical care (16). This highlights the importance of workplace accommodations, such as flexible work hours, remote work options, or lighter workloads, to support employees dealing with persistent symptoms and help them achieve a full recovery while maintaining their employment.

Workers with Long COVID who are older, smokers, exposed to excessive noise, working in confined spaces, and without a sickness absence policy face higher odds of experiencing poor WRQoL due to several factors. Older workers often have weakened immune systems and slower recovery times (17), which can exacerbate fatigue and mobility issues, leading to reduced work capacity. Smoking worsens respiratory symptoms, reduces lung function, and slows the healing process (18), making recovery from Long COVID more challenging. Exposure to excessive noise can contribute to fatigue, stress, headaches, and sleep disturbances (19), all of which may amplify Long COVID symptoms. Additionally, working in confined spaces with poor ventilation can worsen Long COVID symptoms, especially within the respiratory system (20). Lastly, the absence of a sickness absence policy adds to the problem by preventing proper rest and recovery (21), as workers may feel pressured to continue working despite their symptoms, leading to stress and anxiety. All these factors can create a cycle of deteriorating health and diminished WRQoL. As

such, there is an urgent need for supportive workplace policies and accommodations to help mitigate risks and support recovery.

Workers with Long COVID symptoms who are exposed to manual handling have higher odds of sickness absenteeism due to the physical and physiological demands of their work. Chronic fatigue and muscle weakness, common in Long COVID, can make the physical exertion required for tasks like lifting, pushing, or pulling heavy objects difficult to sustain. Persistent joint and muscle pain can be worsened by manual handling (22), leading to the need for time off to recover. Respiratory symptoms, such as dyspnoea, further reduces stamina and makes manual tasks particularly challenging (23). Additionally, post-exertional malaise, where symptoms worsen after physical activity (24), can prolong recovery and contribute to more frequent sickness absences. These factors highlight the need for workplace accommodations, such as task modifications or lighter duties, to help workers manage their symptoms and maintain their employment.

Workers with Long COVID symptoms who engage in shift work and experience a high number of symptoms are more likely to face sickness presenteeism. Shift work disrupts normal sleep patterns and can lead to chronic fatigue (25), which compounds Long COVID symptoms such as exhaustion, brain fog, and muscle pain. The irregular hours can also interfere with the workers' ability to recover, leading to persistent or worsened symptoms. However, many workers may continue to attend work despite their symptoms due to fear of job loss, financial insecurity, or a workplace culture that discourages absences. In addition, the high number of Long COVID symptoms further exacerbates sickness presenteeism, as these workers may be physically and/or mentally unfit to perform their duties effectively but continue working out of necessity or

obligation (26). On the other hand, informing the employer regarding the Long COVID symptoms was a protective factor against sickness presenteeism. When employers are aware of an employee's health condition, they are more likely to implement supportive measures such as flexible work arrangements, reduced workload, or modified duties tailored to the employee's physical and mental limitations (27). These accommodations can help reduce the pressure to attend work while unwell and allow for proper symptom management and recovery. Additionally, open communication can reduce fear of job loss or stigma associated with taking sick leave, creating a more supportive work environment.

Workers with Long COVID symptoms who handle hazardous chemicals may face higher odds of safety issues at work because fatigue and malaise can impair their ability to respond quickly to chemical hazards or emergencies (28). Additionally, cognitive impairments such as brain fog, poor memory, and difficulty concentrating increase the likelihood of errors in handling hazardous chemicals (29). On the other hand, workers with Long COVID who inform their employers about their symptoms may experience fewer safety issues. This is because informing employers allows workers to receive reasonable accommodations (30), such as adjusted workloads, flexible schedules, or reduced exposure to hazardous chemicals, which can help mitigate safety risks.

4.1 Strengths and Limitations of the Study

This study has several key strengths. Firstly, its nationwide coverage, which includes manufacturing workers from all states in Malaysia, enhances the generalisability of its findings. Secondly, to the best of the researchers' knowledge, this is the first study to determine the

prevalence and risk factors of adverse work outcomes among manufacturing workers, offering valuable insights for occupational health policies and interventions. To fully account for potential confounding symptoms or pre-existing conditions that could overlap with or mimic Long COVID, such as bronchial asthma and chronic obstructive pulmonary disease, several measures were incorporated into the study design and data collection process. Firstly, information on pre-existing comorbidities (e.g., respiratory, cardiovascular, and metabolic conditions) was collected and participants were included in the multivariate analysis to allow adjustment for potential confounders. Secondly, participants were required to have a confirmed diagnosis of COVID-19 within a defined time frame (i.e., fully fulfilling the WHO definition), to ensure temporal relevance to Long COVID assessment. Thirdly, symptoms were assessed in relation to their onset following COVID-19 infection, which helped differentiate new or persistent symptoms from pre-existing conditions.

Although the study has notable strengths, it also has some limitations. A major limitation is its cross-sectional design, which prevents the establishment of causal relationships between risk factors and adverse work outcomes. Secondly, the use of convenience sampling may have introduced selection bias, as participants who were more willing or available to participate could differ systematically from those who did not participate. Consequently, the findings may not be fully generalisable to all Malaysian manufacturing workers with Long COVID. Thirdly, the study relied on self-reported information regarding previous COVID-19 infection, Long COVID symptoms, workplace exposures, and adverse work outcomes, which may be subject to recall bias. Participants may have underreported or overreported their symptoms and occupational experiences, particularly for events occurring several months prior to the survey. In addition, there

is a possibility of misclassification bias, particularly in the identification of Long COVID status and workplace exposures, as no objective clinical verification or workplace exposure measurements were performed. Some participants may have inaccurately classified their symptoms or exposures, potentially affecting the observed associations. Nevertheless, the study provides important preliminary evidence on the occupational impacts of Long COVID among manufacturing workers and highlights the need for further prospective studies using probability sampling methods and objective assessments.

4.2 Recommendations for Future Studies

Future research should consider using a longitudinal study design to determine causal relationships between risk factors and adverse work outcomes. Additionally, integrating objective clinical assessments alongside self-reported data could help reduce recall bias and improve the accuracy of symptom reporting. This may include medical evaluations, workplace health monitoring, or biomarkers for Long COVID. To improve generalisability, future studies should expand beyond the manufacturing sector to include workers from various industries with different occupational exposures. This would provide a broader understanding of Long COVID's impact across work environments and support the development of industry-specific recommendations.

4.3 Study Implications

The findings of this study have important implications for workplace interventions and occupational health policies. Firstly, identifying key risk factors for adverse work outcomes

underscores the need for targeted workplace interventions. Employers should implement preventive measures such as enhancing workplace ventilation, minimising exposure to hazardous chemicals, and addressing ergonomic risks associated with prolonged VDU use. Additionally, the study highlights the importance of early detection and management of Long COVID symptoms among workers, which could be facilitated through regular health screenings and medical support programmes. Moreover, the findings suggest that the lack of a structured sickness absence policy may contribute to negative work outcomes. Employers should establish clear guidelines on sickness absence and return-to-work strategies to support employees recovering from Long COVID. Furthermore, the study indicates that disclosing Long COVID symptoms to employers may have a protective effect, emphasising the importance of fostering a supportive and open work environment. This calls for awareness campaigns and training programmes to educate both employers and employees on the impact of Long COVID and the benefits of workplace adjustments.

The study also has wider implications for organisational health policies and regulations. Policymakers should incorporate Long COVID management into existing occupational health and safety frameworks to ensure adequate support for affected workers. Furthermore, based on the study's identification of specific risk factors, future workplace guidelines could include sector-specific recommendations to help reduce the impact of Long COVID.

5. Conclusions

Three-quarters of the manufacturing workers with Long COVID symptoms demonstrated high WRQoL despite their illness. Approximately four in 10 workers with Long COVID symptoms had issues of sickness presenteeism while about 1 in 10 workers with Long COVID workers had issues of sickness absenteeism. A very small number of workers with Long COVID symptoms had safety issues at work. Individual factors (i.e., older age, female gender, and smoking), Long COVID symptoms (i.e., increased number of Long COVID symptoms), work factor (i.e., engaged in shift work, handling hazardous chemicals, exposure to confined space entry, and exposure to excessive noise), organisational factor (i.e., absence of sickness absence policy) were associated with adverse work outcomes among workers with Long COVID symptoms. Meanwhile, personal factors such as informing employers about Long COVID symptoms seem to have a protective effect on adverse work outcomes among the workers with Long COVID symptoms.

Declarations

Consent for publication: Not applicable.

Availability of data and materials: Data cannot be shared publicly. This is because even when personal identifiers are removed, individuals may still be re-identified from combined data (e.g., department, position in the company, work schedule, etc.), especially in workplaces with small numbers of workers. Additionally, the Personal Data Protection Act 2010 restricts the sharing of sensitive health information. Furthermore, the ethics committee limits data use to research purposes, and public sharing may violate the conditions under which participants originally consented. However, data are available from the Research Ethics Committee of the National

University of Malaysia (contact via +603-9145 5046 / +603-9145 5048) for researchers who meet the criteria for access to confidential data.

Competing interests: The authors declare that they have no competing interests.

Funding: This work was supported by the National Institute of Occupational Safety and Health (NIOSH) Malaysia [grant number: UKMP-S230424] to [HMY]. However, the funder had no role in study design, data collection and analysis, decision to publish, or preparation of the manuscript.

Authors' contributions: HMY, SQY, MAMN, AAR, and RD conceptualised and designed the study. Data collection was conducted by MHMH, SR, ZM, RAR, MS, AAB, IRI, FM, AS, and KA. SQY, AMN, NBAK, RI, and NMT carried out the statistical analysis. SQY prepared the initial draft of the manuscript. All authors participated in interpreting the results, provided critical revisions for intellectual significance, and approved the final version for publication.

Acknowledgements: We would like to thank the Director of the National Institute of Occupational Safety and Health (NIOSH) Malaysia, for its grant and permission to publish this article. As specified in the terms of reference, the study will focus exclusively on manufacturing workers.

S1 Table: Association between various risk factors and poor work-related quality of life among manufacturing workers with Long COVID

S2 Table: Association between various risk factors and sickness absenteeism among manufacturing workers with Long COVID

S3 Table: Association between various risk factors and sickness presenteeism among manufacturing workers with Long COVID

S4 Table: Association between various risk factors and safety issues at work among manufacturing workers with Long COVID

S5 Table: Moderating effects in the association between Long COVID and adverse work outcomes

References:

1. DOSM. Gross Domestic Product Malaysia. 2025.
2. DOSM. Department of Statistics Malaysia. 2023.
3. Alaaraj S, Mohamed ZA, Ahmad Bustamam U. Do Knowledge Management Capabilities Reduce the Negative effect of Environment Uncertainties on Organizational Performance? A Study of Public Listed Companies in Malaysia. International Journal of Economic Research. 2017;14:443-56.
4. Gonzalez AE, Suzuki E. OECD Health Working Paper No. 167: The impacts of Long COVID across OECD countries. United Kingdom: Organisation for Economic Co-operation and Development (OECD); 2024.
5. WHO. A clinical case definition of post COVID-19 condition by a Delphi consensus, 6 October 2021. 2021.
6. Soriano JB, Murthy S, Marshall JC, Relan P, Diaz JV, Condition WHOCCDWGoP-C-. A clinical case definition of post COVID-19 condition by a Delphi consensus. Lancet Infect Dis. 2022;22(4):e102-e7.
7. Gualano MR, Rossi MF, Borrelli I, Santoro PE, Amantea C, Daniele A, et al. Returning to work and the impact of post COVID-19 condition: A systematic review. Work. 2022;73(2):405-13.
8. Nehme M, Vieux L, Kaiser L, Chappuis F, Chenaud C, HealthCo Study T, et al. The longitudinal study of subjective wellbeing and absenteeism of healthcare workers considering post-COVID condition and the COVID-19 pandemic toll. Sci Rep. 2023;13(1):10759.
9. Silva Andrade B, Siqueira S, de Assis Soares WR, de Souza Rangel F, Santos NO, dos Santos Freitas A, et al. Long-COVID and Post-COVID Health Complications: An Up-to-Date Review on Clinical Conditions and Their Possible Molecular Mechanisms. Viruses. 2021;13(4):700.
10. Aiyegbusi OL, Hughes SE, Turner G, Rivera SC, McMullan C, Chandan JS, et al. Symptoms, complications and management of Long COVID: a review. J R Soc Med. 2021;114(9):428-42.
11. Kish L. Survey Sampling: Wiley; 1995 1995.

12. Chen W-S, Haniff J, Siau C-S, Seet W, Loh S-F, Abd Jamil MH, et al. Psychometric Properties of the Malay Work-Related Quality of Life (WRQoL) Scale in Malaysia. WJSSR. 2014;1(1):57.
13. Reilly MC, Zbrozek AS, Dukes EM. The validity and reproducibility of a work productivity and activity impairment instrument. Pharmacoeconomics. 1993;4(5):353-65.
14. Yusoff HM, Mohamed NC, Yew SQ, Nawi AM, Rashidah Ismail OH, Tohit NM, et al. Prevalence and risk factors of Long COVID and its associated adverse work outcomes among workers in the manufacturing sector in Malaysia – a mixed-methods study protocol. J Health Popul Nutr. 2024;43(1):194.
15. Sun C, Liu Z, Li S, Wang Y, Liu G. Impact of Long COVID on Health-Related Quality of Life Among Patients After Acute COVID-19 Infection: A Cross-Sectional Study. Inquiry. 2024;61:469580241246461.
16. Brouwer W, Verbooy K, Hoefman R, van Exel J. Production Losses due to Absenteeism and Presenteeism: The Influence of Compensation Mechanisms and Multiplier Effects. Pharmacoeconomics. 2023;41(9):1103-15.
17. Weyand CM, Goronzy JJ. Aging of the Immune System. Mechanisms and Therapeutic Targets. Ann Am Thorac Soc. 2016;13 Suppl 5(Suppl 5):S422-S8.
18. Arcavi L, Benowitz NL. Cigarette smoking and infection. Arch Intern Med. 2004;164(20):2206-16.
19. Kim J, Lee W, Won J-U, Yoon J-H, Seok H, Kim Y-K, et al. The relationship between occupational noise and vibration exposure and headache/eyestrain, based on the fourth Korean Working Condition Survey (KWCS). PLoS One. 2017;12(5):e0177846.
20. Moon J, Ryu B-H. Transmission risks of respiratory infectious diseases in various confined spaces: A meta-analysis for future pandemics. Environ Res. 2021;202:111679.
21. Keus van de Poll M, Nybergh L, Lornudd C, Hagberg J, Bodin L, Kwak L, et al. Preventing sickness absence among employees with common mental disorders or stress-related symptoms at work: a cluster randomised controlled trial of a problem-solving-based intervention conducted by the Occupational Health Services. Occup Environ Med. 2020;77(7):454-61.
22. Mueller C, Sauter M, Barthelme J, Liebers F. The association between manual handling operations and pain in the hands and arms in the context of the 2018 BIBB/BAuA Employment Survey. BMC Musculoskelet Disord. 2021;22(1):644.
23. Boadu EF, Okeke SR, Boadi C, Osei Bonsu E, Addo IY. Work-related respiratory health conditions among construction workers: a systematic narrative review. BMJ Open Respir Res. 2023;10(1):e001736.
24. Appelman B, Charlton BT, Goulding RP, Kerkhoff TJ, Breedveld EA, Noort W, et al. Muscle abnormalities worsen after post-exertional malaise in Long COVID. Nat Commun. 2024;15(1):17.
25. Åkerstedt T, Wright KP. Sleep Loss and Fatigue in Shift Work and Shift Work Disorder. Sleep Med Clin. 2009;4(2):257-71.
26. Mendola M, Leoni M, Cozzi Y, Manzari A, Tonelli F, Metruccio F, et al. Long-term COVID symptoms, work ability and fitness to work in healthcare workers hospitalized for SARS-CoV-2 infection. Med Lav. 2022;113(5):e2022040.
27. Bosma AR, Boot CRL, Snippen NC, Schaafsma FG, Anema JR. Supporting employees with chronic conditions to stay at work: perspectives of occupational health professionals and organizational representatives. BMC Public Health. 2021;21(1):592.

28. Cunningham TR, Guerin RJ, Ferguson J, Cavallari J. Work-related fatigue: A hazard for workers experiencing disproportionate occupational risks. Am J Ind Med. 2022;65(11):913-25.
29. Jung S, Pak S, Lee K, Kang C. Classification of Human Failure in Chemical Plants: Case Study of Various Types of Chemical Accidents in South Korea from 2010 to 2017. Int J Environ Res Public Health. 2021;18(21):11216.
30. Jansen J, Snippen N, Koning P, Boot C, van Ooijen R, Brouwer S. Discrepancies between workers with disabilities and their supervisors in reported work accommodations and associations with return to work. BMC Public Health. 2023;23(1):525.

The conceptual framework of the study

Click here to access/download;Figure;Figure 1.JPG

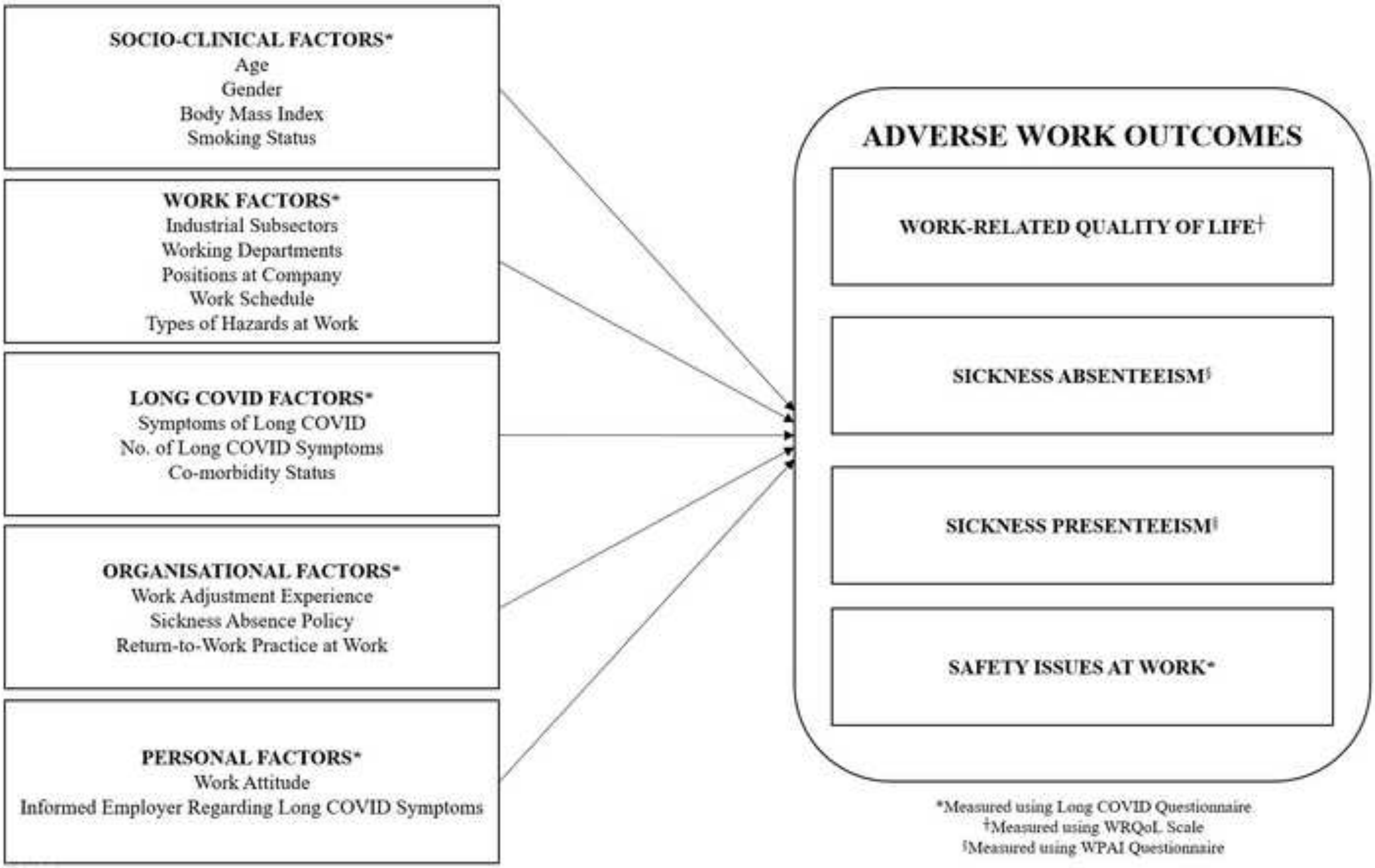

Click here to access/download
Supporting Information
S1_Table.docx

Click here to access/download
Supporting Information
S2_Table.docx

Click here to access/download
Supporting Information
S3_Table.docx

Click here to access/download
Supporting Information
S4_Table.docx

Full Title: Prevalence and risk factors of adverse work outcomes among manufacturing workers with Long COVID in Malaysia – a nationwide study

Short Title: Adverse work outcomes among manufacturing workers with Long COVID

Hanizah Mohd Yusoff [a], Yew Sheng Qian [a]*, Azmawati Mohd Nawi [a], Noorlaili Mohd Tohit [b], Nor Ba'yah Abdul Kadir [c], Anita Abd Rahman [d], Rahmat Dapari [d], Ohnmar Htwe [e], Muhamad Hazizi Muhamad Hasani [f], Sivarajan Ramasamy [f], Zuraida Mohamad [f], Radzi Abdul Rashid [g], Mazrura Sahani [h], Azuraliza Abu Bakar [i], Ida Rosnita Ismail [j], Muhamad Ariff Muhamad Noordin [k], Fazlin Mohd [l], Amalia Sakina [l], Kamilah Amnani [l]

[a] Department of Public Health Medicine, Faculty of Medicine, Universiti Kebangsaan Malaysia, Bandar Tun Razak, Cheras, Kuala Lumpur, Malaysia.

[b] Department of Family Medicine, Faculty of Medicine, Universiti Kebangsaan Malaysia, Bandar Tun Razak, Cheras, Kuala Lumpur, Malaysia.

[c] Faculty of Social Sciences and Humanitarian, Universiti Kebangsaan Malaysia, Bangi, Selangor, Malaysia.

[d] Faculty of Medicine and Health Science, Universiti Putra Malaysia, UPM Serdang, Selangor, Malaysia.

[e] Faculty of Medicine, Universiti Sultan Zainal Abidin Medical, Kuala Terengganu, Terengganu, Malaysia.

[f] Negeri Sembilan State Health Department, Seremban, Negeri Sembilan, Malaysia.

[g] OSH ProCare Consultant and Services, G&C Township, Seremban, Negeri Sembilan, Malaysia.

[h] Centre of Health and Applied Sciences, Faculty of Health Sciences, Universiti Kebangsaan Malaysia, Kuala Lumpur, Malaysia.

[i] Faculty of Information Science and Technology, Universiti Kebangsaan Malaysia, UKM, Bangi, Selangor, Malaysia.

[j] Graduate School of Business, Universiti Kebangsaan Malaysia, Bangi, Selangor, Malaysia.

[k] Research Management Center, National Institute of Occupational Safety and Health, Bandar Baru Bangi, Selangor, Malaysia.

[l] UKM Pakarunding Sdn Bhd, Level 3, Universiti Kebangsaan Malaysia, Bangi, Selangor, Malaysia.

Correspondence:

Sheng Qian Yew (SQY)

Email: shengqian@ukm.edu.my

Tel: 03-91458792

Abstract

Introduction: The increasing prevalence and multiorgan involvement of Long COVID among the general population has raised concerns about the possible long-term effect of this condition among industrial workers. Therefore, this study seeks to determine the prevalence and risk factors of adverse work outcomes among Malaysian manufacturing workers affected by Long COVID.

Materials and Methods: A cross-sectional survey was conducted using the Long COVID Questionnaire, Work-Related Quality of Life (WRQoL) Scale, and the Work Productivity and

Activity Impairment (WPAI) Questionnaire, which were physically distributed to previously-COVID-19 infected participants via convenient sampling. Simple logistic regression and multiple logistic regression were used to determine the association between the various risk factors and adverse work outcomes.

Results: Among the 797 participants, a quarter (24.7%) of the workers with Long COVID demonstrated poor WRQoL. A total of 12.8% and 42.4% of workers with Long COVID had issues of sickness absenteeism and sickness presenteeism, respectively. Only 6.3% of the Long COVID workers had safety issues at work. Socio-clinical factors [i.e., older age (aOR=1.02) and smoking (aOR=1.38)], Long COVID factors [i.e., increased number of Long COVID symptoms (aOR=1.15)], work factors [i.e., engaged in shift work (aOR=1.25), handling of chemicals hazardous to health (aOR=2.51), exposure to confined space entry (aOR=1.60), exposure to excessive noise (aOR=1.55), and manual handling (aOR=1.33)], organisational factors [i.e., absence of sickness absence policy (aOR=2.77)] were significant risk factors of adverse work outcomes among workers with Long COVID. Conversely, personal factors [i.e., informing employers about the Long COVID symptom (aOR=0.32-0.58)] had a protective effect on adverse work outcomes.

Conclusions: The impact of Long COVID on WRQoL, sickness absenteeism, sickness presenteeism, safety issues at work, as well as the multifactorial nature of adverse work outcomes highlights the need for targeted workplace interventions, supportive policies, early detection, and inclusive occupational health frameworks at the workplace.

Keywords: Work impact, post-COVID, manufacturer, employer, employee

1. Introduction

The manufacturing sector is a key contributor to Malaysia's economy, accounting for the second-largest share of the country's gross domestic product (GDP) and an annual contribution exceeding RM300 billion (1). Furthermore, it provides employment for approximately 2.33 million individuals as of November 2022 (2). The industry's reliance on skilled labour is crucial for its efficient operation, making the retention of talented, skilled, and experienced workers essential for enhancing overall performance, quality, efficiency, effectiveness, and productivity within the sector (3). Consequently, maintaining a healthy and productive workforce is vital. Unfortunately, the high number of COVID-19 clusters among manufacturing workers during the pandemic, along with the subsequent high prevalence of Long COVID, has raised concerns regarding the long-term sustainability of this sector (4).

The World Health Organization (WHO) defines Long COVID as a condition that develops in individuals with a prior SARS-CoV-2 infection, typically occurring three months after the initial onset of COVID-19 (5). Additionally, the symptoms must persist for at least two months and cannot be attributed to any other underlying condition. Generally, Long COVID presents with a wide range of symptoms, which are thought to result from a pathology similar to that of acute COVID-19. Some of the most commonly reported symptoms include fatigue, shortness of breath, and cognitive impairment (6).

Given its reported impacts on multiple organ systems among the general population, Long COVID could possibly affect daily functioning, including workplace activities. For instance, workers experiencing Long COVID may face various negative work-related consequences, such as decreased productivity and performance, absenteeism, presenteeism, a higher risk of occupational injuries, and a reduced work-related quality of life (7, 8). Furthermore, workers requiring prolonged medical leave for recovery, ongoing treatment, or those unable to return to work may contribute to increased operational expenses for employers. It is also worth mentioning that the extent of these adverse work outcomes may vary depending on several factors, including the availability of workplace adjustment, company policies on sick leave, and return-to-work strategies implemented within the organisation.

While previous research has documented various negative effects of Long COVID, most studies have primarily focused on the general population (9, 10). Consequently, there is limited knowledge regarding the specific impact of Long COVID on work-related outcomes among manufacturing workers. An updated study on the burden of Long COVID within this sector would help workers, employers, policymakers, and other stakeholders better understand the scale of the issue affecting the manufacturing industry. On the basis of these research gaps and this rationale, this study aims to assess the prevalence and risk factors associated with adverse work outcomes among manufacturing workers in Malaysia who are affected by Long COVID.

2. Materials and Methods

A cross-sectional survey was conducted from April 2024 to October 2024 across Malaysia's five geographical zones (i.e., East Coast, Northern, Southern, Central, and East Malaysia). The target population consisted of workers from manufacturing companies listed in the Federation of Malaysian Manufacturers (FMM) directory, with eligibility criteria aligned with those specified in Table 1. Initially, a multistage sampling strategy was planned for the selection of manufacturing companies, involving cluster sampling within each geographical zone based on the total number of companies, followed by proportionate stratified sampling to select workers from the states within each zone. However, due to a very low response rate (e.g., companies declining participation), the researchers opted to use a convenience sampling method instead. Under this approach, any company that provided consent to participate from each zone in Malaysia was included. This resulted in three, four, three, six, and two companies participating in East Coast, Northern, Southern, Central, and East Malaysia, respectively. This was followed by a universal sampling to select workers from each of these companies. Given a precision level of 0.05, a confidence level of 95%, a prevalence of adverse work outcomes of 50.0% (in the absence of data on prevalence of adverse work outcome among workers with Long COVID), and a non-response rate of 50%, the total sample size required nationwide was 768. This sample size estimation was calculated using Kish's formula (11).

Table 1: Inclusion and exclusion criteria for manufacturing workers

Inclusion criteria	Exclusion criteria
1. Workers of the manufacturing company (including electrical, electronics, petroleum,	1. Workers who had resigned or terminated.

chemical, rubber, plastic, non-metallic mineral products, basic metal, fabricated metal, food and beverages, tobacco, wood, furniture, paper products, printing, textile, wearing apparel, leather, footwear, and transport equipment sectors).<br>2. Workers diagnosed with COVID-19 from 1st January 2022 until 30th April 2024.<br>3. Malaysian and legal foreign workers.<br>4. Worked for at least six months prior to the acute phase of COVID-19.<br>5. Able to communicate in English and/or Malay.	2. Temporary workers.<br>3. Pregnant women during data collection or during acute phase of COVID-19.<br>4. Workers who did not consent for participation.

Three previously validated questionnaires, namely the Long COVID Questionnaire, Work-Related Quality of Life (WRQoL) Scale (12), and the Work Productivity and Activity Impairment (WPAI) Questionnaire (13), were utilised to assess the prevalence and risk factors of adverse work outcomes. Permission to use these questionnaires was obtained from their respective developers.

The 67-item Long COVID Questionnaire was used to evaluate both the presence of Long COVID symptoms and the associated adverse work outcomes among manufacturing workers (14). The questionnaire is divided into three sections. Section A comprises seven items designed to collect socio-clinical data, including age, gender, BMI (from self-reported weight and height),

comorbidities, and smoking status. Section B contains 57 items assessing the presence of Long COVID, where a worker is classified as having Long COVID if they respond with “Yes,” “Yes,” and “No” for parts a, b, and c of each symptom listed, respectively. Lastly, Section C consists of three items aimed at identifying workplace safety concerns. A worker is considered to have safety issues at work if they select “unsure,” “affected,” or “very much affected” for at least one item in this section.

The 24-item WRQoL Scale was used to assess the perceived quality of life of the manufacturing workers with Long COVID (12). WRQoL was measured through six psychosocial subscales (i.e., general well-being, home-work interface, job-career satisfaction, control at work, working conditions, and stress at work). For score interpretation, items 7, 9, and 19 are reverse-coded. In this study, the total WRQoL score was derived by summing all 24 item scores, with a score of 83 or higher indicating a high quality of work life, while a score below 83 is classified as low WRQoL.

The 6-item, unidimensional WPAI Questionnaire was employed to evaluate sickness absenteeism and presenteeism among manufacturing workers affected by Long COVID (13). A worker is classified as having sickness absenteeism if they missed at least 1% of their assigned work time in the past seven days. Likewise, sickness presenteeism is identified when a worker reports at least a 1% reduction in work performance due to health issues within the same period. The conceptual framework of the study is shown in Figure 1.

Figure 1: The conceptual framework of the study

Data collection involved on-site visits by 2–5 researchers and/or enumerators at each selected manufacturing company. In total, there were 32 researchers and/or enumerators, who were trained using the standardised data collection protocol. This allowed the study to be conducted nationwide over the same period using a standardised protocol. After obtaining informed consent from the company, the human resource personnel were contacted to provide a list of workers (i.e., the sampling frame) who had been diagnosed with and documented as having acute COVID-19. With the cooperation of the management, the selected workers were assembled in a designated room within the management office. Each worker was then screened for eligibility based on the criteria outlined in Table 1. Eligible workers were provided with a briefing on the study objectives, instructions for questionnaire administration, and their rights before providing informed consent to participate. Those who were deemed ineligible or who declined participation were thanked and permitted to return to their work. Prior to commencing the survey, informed consent was obtained from all eligible workers. They were encouraged to complete the questionnaire with the assistance of the researchers and/or enumerators, and sufficient time was allocated to ensure high-quality data collection. The role of the researcher and/or enumerators was to clarify items and explain complex medical terminology in the questionnaire. Workers' anonymity and confidentiality were upheld throughout the study. To minimise disruptions to workplace operations, data collection was conducted in a staggered manner, with only one to two workers completing the questionnaire at a time.

Descriptive analysis was conducted to present the prevalence of adverse work outcomes and risk factors of adverse work outcomes. Simple logistic regression was performed to examine the association between the various risk factors (including socio-clinical, Long COVID, work,

organisational, and personal factors) and adverse work outcomes. Risk factors with a p-value < 0.25 were included in a multiple logistic regression model. Additionally, the moderating effects of organisational and personal factors on Long COVID symptoms were assessed using multiple logistic regression. All statistical analyses were performed using SPSS version 28.

Ethical approval for this study was obtained from the Research Ethics Committee of the National University of Malaysia (JEP-2023-607) and the Medical Research and Ethics Committee (MREC) of the Ministry of Health Malaysia (NMRR ID-23-03310-H3E). Ethical approval was obtained from the Medical Research and Ethics Committee (MREC) as the study included healthcare professionals from the Ministry of Health Malaysia. Participants recruitment began on 15th January 2024 and ended on 16th December 2024. All participants provided written informed consent prior to their participation. In addition, written or electronic consent was secured from the participating manufacturing companies prior to data collection.

3. Results

In the present study, adverse work outcomes included poor WRQoL, work productivity (sickness absenteeism and sickness presenteeism), and safety issues at work (Table 1). In terms of the prevalence of adverse work outcomes, almost a quarter of the workers with Long COVID (24.7%) exhibited poor WRQoL. Sickness absenteeism and sickness presenteeism were reported by 12.8% and 42.4% of workers with Long COVID, respectively. Only a small number of workers (6.3%) reported experiencing safety issues at work.

Table 1: Prevalence of adverse work outcomes among the manufacturing workers with Long COVID

Adverse work outcomes	Workers with LC, n (%) N = 797
**Work-related quality of life (WRQoL)**	
Poor	197 (24.7)
Good	600 (75.3)
**Work productivity (sickness absenteeism)**	
Yes	102 (12.8)
No	695 (87.2)
**Work productivity (sickness presenteeism)**	
Yes	338 (42.4)
No	459 (57.6)
**Safety issues at work**	
Yes	50 (6.3)
No	747 (93.7)

The risk factors of adverse work outcomes investigated in the present study include socio-clinical, Long COVID, work, organisational, and personal factors. The socio-clinical factors of the manufacturing workers with Long COVID were presented in Table 2. The mean age of the workers was 37.4 ± 9.5 years. Most workers were female (54.5%), overweight (37.6%) and did not have comorbidities (64.4%). Among those with co-morbidities, hypertension (13.2%) was the most

common, followed by asthma (7.8%) and diabetes (5.4%). Additionally, the majority of workers were non-smokers (78.2%).

Table 2: Socio-clinical factors of the manufacturing workers with Long COVID

Individual factors among manufacturing workers	Mean (SD)	n (%) N = 797
**Age (years)**	37.4 (9.5)	-
**Gender**		
Male	-	363 (45.5)
Female	-	434 (54.5)
**BMI category (kg/m^2)**		
< 18.5 (underweight)	-	38 (4.8)
18.5 – 24.9 (normal)	-	276 (34.6)
25.0 – 29.9 (overweight)	-	300 (37.6)
> 30 (obese)	-	183 (23.0)
**Comorbidities**		
Total comorbidities (may overlap)	-	284 (35.6)
Hypertension	-	105 (13.2)
Asthma	-	62 (7.8)
Diabetes	-	43 (5.4)
Anxiety/Panic attack	-	16 (2.0)
Heart disease	-	11 (1.4)

Autoimmune disease	-	10 (1.3)
Chronic kidney disease	-	4 (0.5)
Chronic liver disease	-	1 (0.1)
Cancer	-	0 (0.0)
HIV Infection	-	0 (0.0)
Others[†]	-	94 (11.8)
**Smoking status**		
Non-smokers	-	623 (78.2)
Smokers	-	174 (21.8)

**Range of age = 22 – 63 years old*

[†]*Others include gastritis, dyslipidaemia, gout, and psoriasis.*

As presented in Table 3, among the 797 manufacturing workers with Long COVID, the most common symptoms for Long COVID were post-exertional malaise (50.9%), followed by fatigue (37.4%), and joint pain (25.6%). Of note, the workers experienced a wide range of symptoms, ranging from one symptom to 19 symptoms, with most experiencing only one symptom (35.3%).

Table 3: Long COVID factors of the manufacturing workers

Long COVID symptoms	n (%)<br>N = 797
Post-exertional malaise	406 (50.9)
Fatigue	298 (37.4)

Joint pain	204 (25.6)
Muscle pain	171 (21.5)
Cough	164 (20.6)
Headache	142 (17.8)
Tingling	140 (17.6)
Cognitive dysfunction	136 (17.1)
Dyspnoea	133 (16.7)
Tachycardia	133 (16.7)
Chest Pain	120 (15.1)
Sleeping disturbance	105 (13.2)
Intermittent fever	93 (11.7)
Dizziness	91 (11.4)
Tinnitus	77 (9.7)
Diarrhoea	55 (6.9)
Altered sensation	39 (4.9)
Abdominal pain	27 (3.4)
Depression	26 (3.3)
**No. of Long COVID Symptom(s) Experienced**	
1	281 (35.3)
2	158 (19.8)
3	109 (13.7)
4	76 (9.5)
5	42 (5.3)

6	38 (4.8)
7	22 (2.8)
8	18 (2.3)
9	17 (2.1)
10	13 (1.6)
11	6 (0.8)
12	5 (0.6)
13	7 (0.9)
14	1 (0.1)
15	2 (0.2)
16	1 (0.1)
17	0 (0.0)
18	1 (0.1)
19	0 (0.0)

Table 4 presents the work factors of the manufacturing workers with Long COVID. Most of the workers interviewed were working in the electrical and electronics subsectors (52.3%) and most of them were working in the operation/supply chain department (84.8%)". Additionally, most of the workers were operators (40.8%) and primarily worked normal office hours (51.9%). In terms of types of hazards at work, the most commonly identified workplace hazards included visual display units (VDU) (61.9%), manual handling (40.8%), and excessive noise (29.5%).

Table 4: Description of work factors of the manufacturing workers with Long COVID

Work factors of the manufacturing workers	n (%)<br>N = 797
**Industrial subsectors**	
Electrical and electronics	417 (52.3)
Transport, vehicle and equipment	163 (20.5)
Rubber	119 (14.9)
Textile, wearing apparel and leather	74 (9.3)
Chemical (including petroleum)	11 (1.4)
Paper, printing, and publishing	10 (1.3)
Food, beverage, and tobacco	3 (0.4)
**Working departments**	
Operations/supply chain	676 (84.8)
Research and development (R&D)	29 (3.6)
Human resource	23 (2.9)
Finance (including accounts)	20 (2.5)
Facilities	17 (2.1)
Sales	8 (1.0)
Procurement	8 (1.0)
Marketing	5 (0.6)
Corporate	3 (0.4)
Others	8 (1.0)
**Positions at company**	

Operator	325 (40.8)
Technician, specialist or permit holder	199 (25.0)
Executive	77 (9.7)
Supervisor	74 (9.3)
Officer	47 (5.9)
Manager	46 (5.8)
Clerk	15 (1.9)
General worker	5 (0.6)
Support services	4 (0.5)
Director	1 (0.1)
Others	4 (0.5)
**Work schedule**	
Normal/office hours	414 (51.9)
Shift including staggered shift	382 (47.9)
Flexible	1 (0.1)
Work from home	0 (0.0)
**Types of hazards at work**	
VDU (visual display unit)/video (computer)	493 (61.9)
Manual handling	325 (40.8)
Excessive noise	235 (29.5)
Handling of hazardous chemicals	206 (25.8)
Electrical work	158 (19.8)
Working in extreme temperatures (hot or cold)	99 (12.4)

Confined space entry	91 (11.4)
Working at heights	60 (7.5)
Lone/isolated work	45 (5.6)
Driving vehicle	35 (4.4)
Working on roofs	33 (4.1)
Construction work	30 (3.8)
Driving lift truck	28 (3.5)
┼Others	40 (5.0)

┼*Example: Working with sharp objects and risk of falling objects*

Analysis of the organisational factors found that 53.3% of the workers with Long COVID perceived that work adjustment was feasible at their workplaces (Table 5). Furthermore, almost half (49.4%) of the workers with Long COVID perceived that a sickness absence policy was present at their workplace. Only 32.9% of the workers believed that return-to-work (RTW) practices were present at their workplace.

Table 5: Description of organisational factors of manufacturing workers with Long COVID symptoms

Organisational factors of manufacturing workers with Long COVID symptoms	n (%) N = 797	
	Yes (%)	No (%)
Presence of work adjustment feasibility	425 (53.3)	372 (46.7)

Presence of sickness absence policy at workplace	394 (49.4)	403 (50.6)
Presence of RTW practices at workplace	262 (32.9)	535 (67.1)

In terms of personal factors, most of the workers with Long COVID had a good work attitude (93.9%), despite their condition (Table 6). Among the workers with Long COVID symptoms, most did not inform their employers (89.1%).

Table 6: Description of personal factors of manufacturing workers with Long COVID symptoms

Personal factors of manufacturing workers with Long COVID symptoms	Workers with LC, n (%) N = 797
**Work attitude**	
Good	748 (93.9)
Poor	49 (6.1)
**Informed employer on Long COVID**	
Yes	87 (10.9)
No	710 (89.1)

To investigate the association with WRQoL, a total of 21 risk factors (i.e., socio-clinical, Long COVID, work, organisational, and personal factors with $p < 0.25$ in the simple logistic regression) were modelled using multiple logistic regression (MLR) with a forward method (S1 Table). All assumptions of MLR (i.e., linearity, interaction, multicollinearity, and outliers) were met. Out of

the 21 risk factors, only five risk factors were significantly associated with WRQoL, namely age, smoking status, excessive noise, confined space entry and sickness absence policy at workplace. For workers with Long COVID symptoms, each additional year of age is associated with a 2% increase in the odds of experiencing poor WRQOL (aOR = 1.02, $p < 0.001$). Workers with Long COVID symptoms who smoke have 1.38 times higher odds of suffering from poor WRQOL (aOR = 1.38, $p < 0.001$), compared with those who did not smoke. Workers with Long COVID symptoms who were exposed to excessive noise had 1.55 times higher odds of suffering from poor WRQOL (aOR = 1.55, $p < 0.001$), compared with those who were not exposed. Workers with Long COVID symptoms subjected to confined space entry had 1.60 times higher odds of suffering from poor WRQOL (aOR = 1.60, $p = 0.013$), compared with those who were not exposed. Workers who have experienced the absence of a sickness absence policy at the workplace had 2.77 times higher odds of suffering from poor WRQOL (aOR = 2.77, $p < 0.001$), compared with those who had a sickness absence policy.

To investigate the association with sickness absenteeism, a total of 10 risk factors (i.e., socio-clinical, Long COVID, work, organisational, and personal factors with $p < 0.25$ in the simple logistic regression) were modelled using MLR with a forward method (S2 Table). All assumptions of MLR (i.e., linearity, interaction, multicollinearity, and outliers) were met. Out of the 10 risk factors, only manual handling was significantly associated with sickness absenteeism. Workers with Long COVID symptoms who are exposed to manual handling had 1.33 times higher odds of having sickness absenteeism (aOR = 1.33, $p = 0.037$), compared with those who were not exposed.

To investigate the association with sickness presenteeism, a total of 17 risk factors (i.e., socio-clinical, Long COVID, work, organisational, and personal factors with $p < 0.25$ in the simple logistic regression) were modelled using MLR with a forward method (S3 Table). All assumptions of MLR (i.e., linearity, interaction, multicollinearity, and outliers) were met. Out of the 17 risk factors, only three risk factors were significantly associated with sickness presenteeism, namely work schedule, number of Long COVID symptoms, and informing employers regarding the Long COVID symptoms. Workers with Long COVID symptoms who engaged in shift work had 1.25 times higher odds of having sickness presenteeism (aOR = 1.25, $p = 0.027$), compared with those who engaged in normal working hours. The odds of having sickness presenteeism increased by 1.15 times for every increase in the number of Long COVID symptoms (aOR = 1.15, $p < 0.001$). Informing the employer regarding the Long COVID symptoms has been found to be a protective factor against sickness presenteeism where they had 42% lower odds of having sickness presenteeism (aOR = 0.58, $p = 0.019$), compared with those who did not inform.

To investigate the association with safety issues at work, a total of 26 risk factors (i.e., socio-clinical, Long COVID, work, organisational, and personal factors with $p < 0.25$ in the simple logistic regression) were modelled using MLR with a forward method (S4 Table). All assumptions of MLR (i.e., linearity, interaction, multicollinearity, and outliers) were met. Out of the 26 risk factors, only two risk factors were significantly associated with safety at work, namely handling of chemicals hazardous to health and informing employers regarding Long COVID symptoms. Workers with Long COVID symptoms who handled chemicals hazardous to health had 2.51 times higher odds of having safety issues at work (aOR = 2.51, $p < 0.001$), compared with those who did not handle. Informing the employer regarding their Long COVID symptoms has been found to be

a protective factor against safety issues at work where they had 68% lower odds of having safety issues at work (aOR = 0.32, p = 0.007), compared with those who did not inform.

According to the multiple logistic regression, it was found that none of the organisational factors and personal factors had a moderating effect on the relationship between Long COVID and adverse work outcomes (S5 Table).

4. Discussion

Our findings suggest that a relatively low prevalence (almost a quarter) of workers with Long COVID had poor WRQoL. In fact, a recent study also found that there were no significant differences in the QoL between those with and without Long COVID (15). With regard to the seemingly paradoxical finding of relatively high WRQoL despite the presence of Long COVID symptoms, several explanations are possible. Firstly, sufficient coping strategies among workers may mitigate the perceived impact of persistent symptoms on WRQoL. For instance, work-focused rehabilitation, multidisciplinary inpatient and outpatient rehabilitation, psychoeducation, pacing, and breathing strategies, have been shown to shift focus from symptom monitoring to optimising functional outcomes. These strategies allowed workers affected by Long COVID to maintain good WRQoL. Secondly, many participants may have experienced mild or manageable symptoms (as most participants experienced only one symptom) that did not substantially impair their work functioning, as observed in the low proportion of workers with sickness absenteeism, sickness presenteeism, and safety issues at work. Hence, it can be concluded that although Long COVID

symptoms could affect multiple body systems, these symptoms are manageable and have little impact on WRQoL.

It is noteworthy that although only 12.8% of the workers with Long COVID absent from their work due to the symptoms, a relatively large proportion of workers (42.4%) continued to work despite having Long COVID symptoms. This may lead to reduced productivity, prolonged recovery times, and potential worsening of symptoms due to the lack of rest and proper medical care (16). This highlights the importance of workplace accommodations, such as flexible work hours, remote work options, or lighter workloads, to support employees dealing with persistent symptoms and help them achieve a full recovery while maintaining their employment.

Workers with Long COVID who are older, smokers, exposed to excessive noise, working in confined spaces, and without a sickness absence policy face higher odds of experiencing poor WRQoL due to several factors. Older workers often have weakened immune systems and slower recovery times (17), which can exacerbate fatigue and mobility issues, leading to reduced work capacity. Smoking worsens respiratory symptoms, reduces lung function, and slows the healing process (18), making recovery from Long COVID more challenging. Exposure to excessive noise can contribute to fatigue, stress, headaches, and sleep disturbances (19), all of which may amplify Long COVID symptoms. Additionally, working in confined spaces with poor ventilation can worsen Long COVID symptoms, especially within the respiratory system (20). Lastly, the absence of a sickness absence policy adds to the problem by preventing proper rest and recovery (21), as workers may feel pressured to continue working despite their symptoms, leading to stress and anxiety. All these factors can create a cycle of deteriorating health and diminished WRQoL. As

such, there is an urgent need for supportive workplace policies and accommodations to help mitigate risks and support recovery.

Workers with Long COVID symptoms who are exposed to manual handling have higher odds of sickness absenteeism due to the physical and physiological demands of their work. Chronic fatigue and muscle weakness, common in Long COVID, can make the physical exertion required for tasks like lifting, pushing, or pulling heavy objects difficult to sustain. Persistent joint and muscle pain can be worsened by manual handling (22), leading to the need for time off to recover. Respiratory symptoms, such as dyspnoea, further reduces stamina and makes manual tasks particularly challenging (23). Additionally, post-exertional malaise, where symptoms worsen after physical activity (24), can prolong recovery and contribute to more frequent sickness absences. These factors highlight the need for workplace accommodations, such as task modifications or lighter duties, to help workers manage their symptoms and maintain their employment.

Workers with Long COVID symptoms who engage in shift work and experience a high number of symptoms are more likely to face sickness presenteeism. Shift work disrupts normal sleep patterns and can lead to chronic fatigue (25), which compounds Long COVID symptoms such as exhaustion, brain fog, and muscle pain. The irregular hours can also interfere with the workers' ability to recover, leading to persistent or worsened symptoms. However, many workers may continue to attend work despite their symptoms due to fear of job loss, financial insecurity, or a workplace culture that discourages absences. In addition, the high number of Long COVID symptoms further exacerbates sickness presenteeism, as these workers may be physically and/or mentally unfit to perform their duties effectively but continue working out of necessity or

obligation (26). On the other hand, informing the employer regarding the Long COVID symptoms was a protective factor against sickness presenteeism. When employers are aware of an employee's health condition, they are more likely to implement supportive measures such as flexible work arrangements, reduced workload, or modified duties tailored to the employee's physical and mental limitations (27). These accommodations can help reduce the pressure to attend work while unwell and allow for proper symptom management and recovery. Additionally, open communication can reduce fear of job loss or stigma associated with taking sick leave, creating a more supportive work environment.

Workers with Long COVID symptoms who handle hazardous chemicals may face higher odds of safety issues at work because fatigue and malaise can impair their ability to respond quickly to chemical hazards or emergencies (28). Additionally, cognitive impairments such as brain fog, poor memory, and difficulty concentrating increase the likelihood of errors in handling hazardous chemicals (29). On the other hand, workers with Long COVID who inform their employers about their symptoms may experience fewer safety issues. This is because informing employers allows workers to receive reasonable accommodations (30), such as adjusted workloads, flexible schedules, or reduced exposure to hazardous chemicals, which can help mitigate safety risks.

4.1 Strengths and Limitations of the Study

This study has several key strengths. Firstly, its nationwide coverage, which includes manufacturing workers from all states in Malaysia, enhances the generalisability of its findings. Secondly, to the best of the researchers' knowledge, this is the first study to determine the

prevalence and risk factors of adverse work outcomes among manufacturing workers, offering valuable insights for occupational health policies and interventions. To fully account for potential confounding symptoms or pre-existing conditions that could overlap with or mimic Long COVID, such as bronchial asthma and chronic obstructive pulmonary disease, several measures were incorporated into the study design and data collection process. Firstly, information on pre-existing comorbidities (e.g., respiratory, cardiovascular, and metabolic conditions) was collected and participants were included in the multivariate analysis to allow adjustment for potential confounders. Secondly, participants were required to have a confirmed diagnosis of COVID-19 within a defined time frame (i.e., fully fulfilling the WHO definition), to ensure temporal relevance to Long COVID assessment. Thirdly, symptoms were assessed in relation to their onset following COVID-19 infection, which helped differentiate new or persistent symptoms from pre-existing conditions.

Although the study has notable strengths, it also has some limitations. A major limitation is its cross-sectional design, which prevents the establishment of causal relationships between risk factors and adverse work outcomes. Secondly, the use of convenience sampling may have introduced selection bias, as participants who were more willing or available to participate could differ systematically from those who did not participate. Consequently, the findings may not be fully generalisable to all Malaysian manufacturing workers with Long COVID. Thirdly, the study relied on self-reported information regarding previous COVID-19 infection, Long COVID symptoms, workplace exposures, and adverse work outcomes, which may be subject to recall bias. Participants may have underreported or overreported their symptoms and occupational experiences, particularly for events occurring several months prior to the survey. In addition, there

is a possibility of misclassification bias, particularly in the identification of Long COVID status and workplace exposures, as no objective clinical verification or workplace exposure measurements were performed. Some participants may have inaccurately classified their symptoms or exposures, potentially affecting the observed associations. Nevertheless, the study provides important preliminary evidence on the occupational impacts of Long COVID among manufacturing workers and highlights the need for further prospective studies using probability sampling methods and objective assessments.

4.2 Recommendations for Future Studies

Future research should consider using a longitudinal study design to determine causal relationships between risk factors and adverse work outcomes. Additionally, integrating objective clinical assessments alongside self-reported data could help reduce recall bias and improve the accuracy of symptom reporting. This may include medical evaluations, workplace health monitoring, or biomarkers for Long COVID. To improve generalisability, future studies should expand beyond the manufacturing sector to include workers from various industries with different occupational exposures. This would provide a broader understanding of Long COVID's impact across work environments and support the development of industry-specific recommendations.

4.3 Study Implications

The findings of this study have important implications for workplace interventions and occupational health policies. Firstly, identifying key risk factors for adverse work outcomes

underscores the need for targeted workplace interventions. Employers should implement preventive measures such as enhancing workplace ventilation, minimising exposure to hazardous chemicals, and addressing ergonomic risks associated with prolonged VDU use. Additionally, the study highlights the importance of early detection and management of Long COVID symptoms among workers, which could be facilitated through regular health screenings and medical support programmes. Moreover, the findings suggest that the lack of a structured sickness absence policy may contribute to negative work outcomes. Employers should establish clear guidelines on sickness absence and return-to-work strategies to support employees recovering from Long COVID. Furthermore, the study indicates that disclosing Long COVID symptoms to employers may have a protective effect, emphasising the importance of fostering a supportive and open work environment. This calls for awareness campaigns and training programmes to educate both employers and employees on the impact of Long COVID and the benefits of workplace adjustments.

The study also has wider implications for organisational health policies and regulations. Policymakers should incorporate Long COVID management into existing occupational health and safety frameworks to ensure adequate support for affected workers. Furthermore, based on the study's identification of specific risk factors, future workplace guidelines could include sector-specific recommendations to help reduce the impact of Long COVID.

5. Conclusions

Three-quarters of the manufacturing workers with Long COVID symptoms demonstrated high WRQoL despite their illness. Approximately four in 10 workers with Long COVID symptoms had issues of sickness presenteeism while about 1 in 10 workers with Long COVID workers had issues of sickness absenteeism. A very small number of workers with Long COVID symptoms had safety issues at work. Individual factors (i.e., older age, female gender, and smoking), Long COVID symptoms (i.e., increased number of Long COVID symptoms), work factor (i.e., engaged in shift work, handling hazardous chemicals, exposure to confined space entry, and exposure to excessive noise), organisational factor (i.e., absence of sickness absence policy) were associated with adverse work outcomes among workers with Long COVID symptoms. Meanwhile, personal factors such as informing employers about Long COVID symptoms seem to have a protective effect on adverse work outcomes among the workers with Long COVID symptoms.

Declarations

Consent for publication: Not applicable.

Availability of data and materials: Data cannot be shared publicly. This is because even when personal identifiers are removed, individuals may still be re-identified from combined data (e.g., department, position in the company, work schedule, etc.), especially in workplaces with small numbers of workers. Additionally, the Personal Data Protection Act 2010 restricts the sharing of sensitive health information. Furthermore, the ethics committee limits data use to research purposes, and public sharing may violate the conditions under which participants originally consented. However, data are available from the Research Ethics Committee of the National

University of Malaysia (contact via +603-9145 5046 / +603-9145 5048) for researchers who meet the criteria for access to confidential data.

Competing interests: The authors declare that they have no competing interests.

Funding: This work was supported by the National Institute of Occupational Safety and Health (NIOSH) Malaysia [grant number: UKMP-S230424] to [HMY]. However, the funder had no role in study design, data collection and analysis, decision to publish, or preparation of the manuscript.

Authors' contributions: HMY, SQY, MAMN, AAR, and RD conceptualised and designed the study. Data collection was conducted by MHMH, SR, ZM, RAR, MS, AAB, IRI, FM, AS, and KA. SQY, AMN, NBAK, RI, and NMT carried out the statistical analysis. SQY prepared the initial draft of the manuscript. All authors participated in interpreting the results, provided critical revisions for intellectual significance, and approved the final version for publication.

Acknowledgements: We would like to thank the Director of the National Institute of Occupational Safety and Health (NIOSH) Malaysia, for its grant and permission to publish this article. As specified in the terms of reference, the study will focus exclusively on manufacturing workers.

S1 Table: Association between various risk factors and poor work-related quality of life among manufacturing workers with Long COVID

S2 Table: Association between various risk factors and sickness absenteeism among manufacturing workers with Long COVID

S3 Table: Association between various risk factors and sickness presenteeism among manufacturing workers with Long COVID

S4 Table: Association between various risk factors and safety issues at work among manufacturing workers with Long COVID

S5 Table: Moderating effects in the association between Long COVID and adverse work outcomes

References:

1. DOSM. Gross Domestic Product Malaysia. 2025.
2. DOSM. Department of Statistics Malaysia. 2023.
3. Alaaraj S, Mohamed ZA, Ahmad Bustamam U. Do Knowledge Management Capabilities Reduce the Negative effect of Environment Uncertainties on Organizational Performance? A Study of Public Listed Companies in Malaysia. International Journal of Economic Research. 2017;14:443-56.
4. Gonzalez AE, Suzuki E. OECD Health Working Paper No. 167: The impacts of Long COVID across OECD countries. United Kingdom: Organisation for Economic Co-operation and Development (OECD); 2024.
5. WHO. A clinical case definition of post COVID-19 condition by a Delphi consensus, 6 October 2021. 2021.
6. Soriano JB, Murthy S, Marshall JC, Relan P, Diaz JV, Condition WHOCCDWGoP-C-. A clinical case definition of post COVID-19 condition by a Delphi consensus. Lancet Infect Dis. 2022;22(4):e102-e7.
7. Gualano MR, Rossi MF, Borrelli I, Santoro PE, Amantea C, Daniele A, et al. Returning to work and the impact of post COVID-19 condition: A systematic review. Work. 2022;73(2):405-13.
8. Nehme M, Vieux L, Kaiser L, Chappuis F, Chenaud C, HealthCo Study T, et al. The longitudinal study of subjective wellbeing and absenteeism of healthcare workers considering post-COVID condition and the COVID-19 pandemic toll. Sci Rep. 2023;13(1):10759.
9. Silva Andrade B, Siqueira S, de Assis Soares WR, de Souza Rangel F, Santos NO, dos Santos Freitas A, et al. Long-COVID and Post-COVID Health Complications: An Up-to-Date Review on Clinical Conditions and Their Possible Molecular Mechanisms. Viruses. 2021;13(4):700.
10. Aiyegbusi OL, Hughes SE, Turner G, Rivera SC, McMullan C, Chandan JS, et al. Symptoms, complications and management of Long COVID: a review. J R Soc Med. 2021;114(9):428-42.
11. Kish L. Survey Sampling: Wiley; 1995 1995.

12. Chen W-S, Haniff J, Siau C-S, Seet W, Loh S-F, Abd Jamil MH, et al. Psychometric Properties of the Malay Work-Related Quality of Life (WRQoL) Scale in Malaysia. WJSSR. 2014;1(1):57.
13. Reilly MC, Zbrozek AS, Dukes EM. The validity and reproducibility of a work productivity and activity impairment instrument. Pharmacoeconomics. 1993;4(5):353-65.
14. Yusoff HM, Mohamed NC, Yew SQ, Nawi AM, Rashidah Ismail OH, Tohit NM, et al. Prevalence and risk factors of Long COVID and its associated adverse work outcomes among workers in the manufacturing sector in Malaysia – a mixed-methods study protocol. J Health Popul Nutr. 2024;43(1):194.
15. Sun C, Liu Z, Li S, Wang Y, Liu G. Impact of Long COVID on Health-Related Quality of Life Among Patients After Acute COVID-19 Infection: A Cross-Sectional Study. Inquiry. 2024;61:469580241246461.
16. Brouwer W, Verbooy K, Hoefman R, van Exel J. Production Losses due to Absenteeism and Presenteeism: The Influence of Compensation Mechanisms and Multiplier Effects. Pharmacoeconomics. 2023;41(9):1103-15.
17. Weyand CM, Goronzy JJ. Aging of the Immune System. Mechanisms and Therapeutic Targets. Ann Am Thorac Soc. 2016;13 Suppl 5(Suppl 5):S422-S8.
18. Arcavi L, Benowitz NL. Cigarette smoking and infection. Arch Intern Med. 2004;164(20):2206-16.
19. Kim J, Lee W, Won J-U, Yoon J-H, Seok H, Kim Y-K, et al. The relationship between occupational noise and vibration exposure and headache/eyestrain, based on the fourth Korean Working Condition Survey (KWCS). PLoS One. 2017;12(5):e0177846.
20. Moon J, Ryu B-H. Transmission risks of respiratory infectious diseases in various confined spaces: A meta-analysis for future pandemics. Environ Res. 2021;202:111679.
21. Keus van de Poll M, Nybergh L, Lornudd C, Hagberg J, Bodin L, Kwak L, et al. Preventing sickness absence among employees with common mental disorders or stress-related symptoms at work: a cluster randomised controlled trial of a problem-solving-based intervention conducted by the Occupational Health Services. Occup Environ Med. 2020;77(7):454-61.
22. Mueller C, Sauter M, Barthelme J, Liebers F. The association between manual handling operations and pain in the hands and arms in the context of the 2018 BIBB/BAuA Employment Survey. BMC Musculoskelet Disord. 2021;22(1):644.
23. Boadu EF, Okeke SR, Boadi C, Osei Bonsu E, Addo IY. Work-related respiratory health conditions among construction workers: a systematic narrative review. BMJ Open Respir Res. 2023;10(1):e001736.
24. Appelman B, Charlton BT, Goulding RP, Kerkhoff TJ, Breedveld EA, Noort W, et al. Muscle abnormalities worsen after post-exertional malaise in Long COVID. Nat Commun. 2024;15(1):17.
25. Åkerstedt T, Wright KP. Sleep Loss and Fatigue in Shift Work and Shift Work Disorder. Sleep Med Clin. 2009;4(2):257-71.
26. Mendola M, Leoni M, Cozzi Y, Manzari A, Tonelli F, Metruccio F, et al. Long-term COVID symptoms, work ability and fitness to work in healthcare workers hospitalized for SARS-CoV-2 infection. Med Lav. 2022;113(5):e2022040.
27. Bosma AR, Boot CRL, Snippen NC, Schaafsma FG, Anema JR. Supporting employees with chronic conditions to stay at work: perspectives of occupational health professionals and organizational representatives. BMC Public Health. 2021;21(1):592.

28. Cunningham TR, Guerin RJ, Ferguson J, Cavallari J. Work-related fatigue: A hazard for workers experiencing disproportionate occupational risks. Am J Ind Med. 2022;65(11):913-25.
29. Jung S, Pak S, Lee K, Kang C. Classification of Human Failure in Chemical Plants: Case Study of Various Types of Chemical Accidents in South Korea from 2010 to 2017. Int J Environ Res Public Health. 2021;18(21):11216.
30. Jansen J, Snippen N, Koning P, Boot C, van Ooijen R, Brouwer S. Discrepancies between workers with disabilities and their supervisors in reported work accommodations and associations with return to work. BMC Public Health. 2023;23(1):525.

Responses to Reviewers

We would like to thank the reviewer for further evaluation of our manuscript and for the constructive and insightful comments provided. We have carefully considered all suggestions and have revised the manuscript accordingly to improve its clarity, methodological transparency, and overall robustness, as follows.

Reviewer 1

1. Abstract: The amendment has been acknowledged and significantly improved. I would, however, recommend using the phrase ‘increasing prevalence’ instead of ‘high prevalence’, suggesting that the actual prevalence remains uncertain.

   RESPONSE: We totally agree that the terminology of “increasing prevalence” is more appropriate that “high prevalence”. As such, the terminology has been revised accordingly.

2. Abstract: The amendment has been acknowledged and significantly improved. I do, however, have a minor suggestion for you to consider using the phrase ‘in contrast’ or ‘conversely’ instead of ‘meanwhile’. This would create a distinct shift in tone in the results, specifically from risk factors to protective factors.

   RESPONSE: Indeed, “conversely” provide more linguistic clarity than “meanwhile”. This terminology has been revised as suggested.

3. Introduction: Para 1, Line 81-83: To cite the reference used.

   RESPONSE: The statement in Line 81-83 has been added with a proper reference from the OECD, as follows: “Gonzalez AE, Suzuki E. OECD Health Working Paper No. 167: The impacts of long COVID across OECD countries. United Kingdom: Organisation for Economic Co-operation and Development (OECD); 2024”.

4. Methods: Line 165: To prevent referencing tables or figures with the term ‘below’, since the figure might appear on the following page, it is adequate to refer to the tables or figures by stating, for example, “as illustrated in Figure 1”.

   RESPONSE: Thank you for the reminder. The use of terminology such as “below” has been removed.

5. Background data: Consider presenting the geographic locations of the companies involved in this study. This would help readers better assess the representativeness of the sample and the generalisability of the findings to Malaysian manufacturing companies.

   RESPONSE: The geographical location of the included companies has been presented in Line 126-127: “This resulted in three, four, three, six, and two companies participated across East Coast, Northern, Southern, Central, and East Malaysia”.

6. Although it is included in Table 2 (currently referred to as Table 1), I suggest that the socio-clinical data of the participants be presented separately.

RESPONSE: The socio-clinical data of the participants has been presented separately in Table 2.

7. Prevalence of adverse work outcomes: The prevalence is not explicitly mentioned. It remains uncertain whether the WRQoL results reflect the prevalence of negative work outcomes. In the subsequent sections (Discussion and Conclusion), different narratives were provided i.e., poor vs high WRQoL. Consider rephrasing.

   RESPONSE: Apology for the confusion and inconsistency. Indeed, the prevalence of poor WRQoL was one of the adverse work outcomes, along with sickness absenteeism, sickness presenteeism, and safety issues at work (Table 1). To ensure better clarity, the sentence has been rephrased as such: "In terms of the prevalence of adverse work outcomes, almost a quarter of the workers with Long COVID (24.7%) exhibited poor WRQoL. Sickness absenteeism and sickness presenteeism were reported by 12.8% and 42.4% of workers with Long COVID, respectively. Only a small number of workers (6.3%) reported experiencing safety issues at work".

8. Risk factors: In my opinion, Table 3-7 solely present the data in percentage rather than factors that are determined by statistical analysis. I would strongly suggest renaming the table titles to prevent any potential misunderstanding among readers. Consider indicating the type of statistical analysis used, as footnote, in all tables provided as Appendix 1-4.

   RESPONSE: Thank you for the suggestion. To prevention misunderstanding to the tables, the terminology of "description of" was added to the title of the relevant tables, to illustrate that the tables are purely descriptive in nature, without inferential analysis. Meanwhile, all appendices were revised with the statistical analyses (i.e., simple and multiple logistic regressions) being stated as footnotes.

Reviewer 2

1. Limitations due to recruitment, selection bias (convenience sampling); in addition to the risk of recall bias, potential misclassification bias should be discussed (if the questionnaire-based algorithm lack clinical validation). Revise wording that may imply overinterpretation or strong claims.

   RESPONSE: Indeed, we agree that these limitations are very relevant. A detailed discussion on these limitations has been added, as follows: "the use of convenience sampling may have introduced selection bias, as participants who were more willing or available to participate could differ systematically from those who did not participate. Consequently, the findings may not be fully generalisable to all Malaysian manufacturing workers with Long COVID. Thirdly, the study relied on self-reported information regarding previous COVID-19 infection, Long COVID symptoms, workplace exposures, and adverse work outcomes, which may be subject to recall bias. Participants may have underreported or overreported their symptoms and occupational experiences, particularly for events occurring several months prior to the survey. In addition, there is a possibility of misclassification bias, particularly in the identification of Long COVID status and workplace exposures, as no objective clinical verification or workplace exposure measurements were performed. Some participants may have

inaccurately classified their symptoms or exposures, potentially affecting the observed associations. Nevertheless, the study provides important preliminary evidence on the occupational impacts of Long COVID among manufacturing workers and highlights the need for further prospective studies using probability sampling methods and objective assessments".

2. Proofreading to improve language and grammar throughout, ensure consistency of terminology and review clarity of table titles, labels/captions.

   RESPONSE: The revised manuscript has been subjected to proofreading to improve on language and grammar use, consistency of terminology and clarity of table titles, labels/captions.
