# Supplementary material for: Prevalence and risk factors of adverse work outcomes among manufacturing workers with Long COVID in Malaysia – A nationwide study
Source: PLoS One. 2026 Sep 11;21(9):e0357125. doi: 10.1371/journal.pone.0357125 (PMC13567834; doi:10.1371/journal.pone.0357125)
Supplement: Supplementary file 6 [file pone.0357125.s001.docx]

**S1 Table: Association between various risk factors and poor work-related quality of life among manufacturing workers with Long COVID**

| **Risk Factors** | **Crude OR***  **(95% CI)** | **P-Value** | **Adjusted OR^┼^**  **(95% CI)** | **P-Value** |
| --- | --- | --- | --- | --- |
| **SOCIO-CLINICAL FACTORS** | | | | |
| **Age (years old)** | 1.03 (1.01-1.05) | 0.002* | 1.02 (1.01-1.04) | <0.001* |
| **Gender** | | | | |
| Male | Ref | - |  |  |
| Female | 0.83 (0.60-1.14) | 0.260 |  |  |
| **BMI Category (kg/m^2^)** | | | | |
| < 18.5 (underweight) | Ref | - |  |  |
| 18.5 – 24.9 (normal) | 0.91 (0.56-1.48) | 0.710 |  |  |
| 25.0 – 29.9 (overweight) | 1.11 (0.68-1.81) | 0.686 |  |  |
| > 30 (obese) | 0.92 (0.55-1.53) | 0.741 |  |  |
| **Smoking Status** | | | | |
| Non-smoker | Ref | - | Ref | - |
| Smoker | 1.24 (0.94-1.63) | 0.129 | 1.38 (1.03-1.85) | 0.034* |
| **WORK FACTORS** | | | | |
| **Industrial Subsectors** | | | | |
| Machinery and Equipment | Ref | - |  |  |
| Chemical (including Petroleum) | 0.44 (0.03-7.67) | 0.577 |  |  |
| Electrical and Electronics | 1.08 (0.23-5.10) | 0.923 |  |  |
| Paper, Printing and Publishing | 0.89 (0.10-7.86) | 0.916 |  |  |
| Transport, Vehicle and Equipment | 0.50 (0.11-2.41) | 0.389 |  |  |
| Rubber | 0.31 (0.06-1.48) | 0.341 |  |  |
| Textile, Wearing Apparel and Leather | 0.56 (0.11-2.82) | 0.482 |  |  |
| **LONG COVID SYMPTOMS** | | | | |
| **Symptoms of Long Covid** | | | | |
| Post-exertional malaise | 0.77 (0.56-1.07) | 0.118 |  |  |
| Fatigue | 0.59 (0.43-0.82) | **0.002*** |  |  |
| Joint Pain | 1.06 (0.35-1.66) | 1.000 |  |  |
| Muscle pain | 1.06 (0.44-1.69) | 1.030 |  |  |
| Cough | 1.02 (0.88-1.87) | 0.998 |  |  |
| Headache | 6.62 (0.60-73.54) | 0.724 |  |  |
| Tingling | 3.31 (0.21-53.22) | 0.398 |  |  |
| Cognitive Dysfunction | 0.65 (0.43-0.97) | **0.037*** |  |  |
| Dyspnoea | 0.96 (0.55-1.69) | 1.000 |  |  |
| Tachycardia | 0.52 (0.35-0.78) | **0.001*** |  |  |
| Chest Pain | 0.46 (0.30-0.69) | **<0.001*** |  |  |
| Sleeping Disturbance | 0.55 (0.36-0.86) | **0.008*** |  |  |
| Intermittent Fever | 0.68 (0.42-1.09) | 0.611 |  |  |
| Dizziness | 0.55 (0.35-0.88) | **0.013*** |  |  |
| Tinnitus | 0.42 (0.26-0.68) | **<0.001*** |  |  |
| Diarrhoea | 0.46 (0.26-0.82) | **0.008*** |  |  |
| Altered Sensation | 0.70 (0.35-1.14) | 0.320 |  |  |
| Abdominal Pain | 0.55 (0.25-1.21) | 0.638 |  |  |
| Depression | 0.61 (0.27-1.39) | 0.280 |  |  |
| **Number of Long Covid Symptoms** | 0.86 (0.82-0.91) | **<0.001*** |  |  |
| **Co-morbidity Status** | | | | |
| No | Ref | - |  |  |
| Yes | 0.97 (0.69-1.36) | 0.838 |  |  |
| **ORGANISATIONAL FACTORS** | | | | |
| **Experienced Work Adjustment** | | | | |
| Yes | Ref | - |  |  |
| No | 0.75 (0.44-1.28) | 0.288 |  |  |
| **Sickness Absence Policy at Workplace** | | | | |
| Yes | Ref | - | Ref | - |
| No | 2.55 (1.82-3.58) | **<0.001*** | 2.77 (2.18-3.52) | **<0.001*** |
| **Experienced RTW Practice at Workplace** | | | | |
| Yes | Ref | - |  |  |
| No | 1.39 (0.81-2.36) | **0.230** |  |  |
| **PERSONAL FACTORS** | | | | |
| **Work Attitude** | | | | |
| Good | Ref | - |  |  |
| Poor | 9.03 (4.74-17.19) | **<0.001*** |  |  |
| **Employer Informed Regarding Long Covid Symptom** | | | | |
| Yes | Ref | - |  |  |
| No | 1.17 (0.71-1.92) | 0.550 |  |  |

*Determined via simple logistric regression

**^┼^** Determined via multiple logistic regression
