# Supplementary material for: Prevalence and risk factors of adverse work outcomes among manufacturing workers with Long COVID in Malaysia – A nationwide study
Source: PLoS One. 2026 Sep 11;21(9):e0357125. doi: 10.1371/journal.pone.0357125 (PMC13567834; doi:10.1371/journal.pone.0357125)
Supplement: Supplementary file 7 [file pone.0357125.s002.docx]

**S2 Table: Association between various risk factors and sickness absenteeism among manufacturing workers with Long COVID**

| **Risk Factors** | **Crude OR***  **(95% CI)** | **P-Value** | **Adjusted OR^┼^**  **(95% CI)** | **P-Value** |
| --- | --- | --- | --- | --- |
| **SOCIO-CLINICAL FACTORS** | | | | |
| **Age (years old)** | 1.00 (0.98-1.01) | 0.807 |  |  |
| **Gender** | | | | |
| Male | Ref | - |  |  |
| Female | 1.41 (0.92-2.16) | 0.114 |  |  |
| **BMI Category (kg/m^2^)** | | | | |
| < 18.5 (underweight) | Ref | - |  |  |
| 18.5 – 24.9 (normal) | 0.56 (0.21-1.45) | 0.279 |  |  |
| 25.0 – 29.9 (overweight) | 0.82 (0.32-2.09) | 0.678 |  |  |
| > 30 (obese) | 1.05 (0.40-2.72) | 0.927 |  |  |
| **Smoking Status** | | | | |
| Non-smoker | Ref | - |  |  |
| Smoker | 0.92 (0.55-1.53) | 0.745 |  |  |
| **WORK FACTORS** | | | | |
| **Industrial Subsectors** | | | | |
| Machinery and Equipment | Ref | - |  |  |
| Chemical (including Petroleum) | 0.99 (0.89-1.69) | 0.998 |  |  |
| Electrical and Electronics | 0.10 (0.05-1.31) | 0.891 |  |  |
| Paper, Printing and Publishing | 1.25 (0.57-2.75) | 0.574 |  |  |
| Transport, Vehicle and Equipment | 2.06 (0.37-11.45) | 0.408 |  |  |
| Rubber | 1.50 (0.64-3.49) | 0.353 |  |  |
| Textile, Wearing Apparel and Leather | 0.93 (0.36-2.38) | 0.872 |  |  |
| **Working Departments** | | | | |
| Departments Requiring Lower Physical Activity | Ref | - |  |  |
| Departments Requiring Higher Physical Activity | 1.15 (0.62-2.14) | 0.657 |  |  |
| **Positions at Company** | | | | |
| Job Requiring Lower Physical Demand | Ref | - |  |  |
| Job Requiring High Physical Demand | 1.24 (0.82-1.88) | 0.310 |  |  |
| **Work Schedule** | | | | |
| Normal/Office hours | Ref | - |  |  |
| Shift including Staggered Shift | 0.91 (0.60-1.38) | 0.664 |  |  |
| **Types of Hazards at Work** | | | | |
| VDU (Visual Display Unit) / Video (Computer) | 1.28 0.76-2.15 | 0.349 |  |  |
| Manual Handling | 1.58 0.98-2.56 | 0.062 | 1.33 (1.02-1.74) | **0.037*** |
| Excessive Noise | 0.60 0.33-1.09 | 0.095 |  |  |
| Handling of Chemical Hazardous to Health | 0.92 0.51-1.67 | 0.793 |  |  |
| Electrical Work | 1.19 0.66-2.14 | 0.561 |  |  |
| Working in extreme temperatures (hot or cold) | 2.17 1.09-4.32 | **0.028*** |  |  |
| Confined Space Entry | 0.81 0.33-1.95 | 0.633 |  |  |
| Working at Heights | 1.16 0.35-3.78 | 0.811 |  |  |
| Driving Vehicle | 2.33 0.78-6.93 | 0.129 |  |  |
| Lone / Isolated Work | 0.38 0.08-1.76 | 0.214 |  |  |
| Driving Lift Truck | 0.23 0.03-2.14 | 0.199 |  |  |
| Construction Work | 0.99 0.69-1.22 | 0.998 |  |  |
| Working on Roofs | 0.78 0.13-4.53 | 0.781 |  |  |
| Others | 0.66 0.19-2.29 | 0.516 |  |  |
| **LONG COVID SYMPTOMS** | | | | |
| **Symptoms of Long Covid** | | | | |
| Post-exertional malaise | 0.92 (0.60-1.39) | 0.678 |  |  |
| Fatigue | 0.86 (0.56-1.34) | 0.509 |  |  |
| Joint Pain | 1.10 (0.69-1.67) | 0.934 |  |  |
| Muscle pain | 0.69 (0.33-1.57) | 0.960 |  |  |
| Cough | 1.04 (0.58-1.67) | 1.000 |  |  |
| Headache | 0.30 (0.03-3.30) | 0.322 |  |  |
| Tingling | 0.99 (0.24-1.96) | 1.000 |  |  |
| Cognitive Dysfunction | 0.90 (0.51-1.59) | 0.718 |  |  |
| Dyspnoea | 1.23 (0.66-1.67) | 1.000 |  |  |
| Tachycardia | 1.18 (0.69-2.02) | 0.548 |  |  |
| Chest Pain | 0.98 (0.55-1.76) | 0.946 |  |  |
| Sleeping Disturbance | 0.86 (0.46-1.64) | 0.652 |  |  |
| Intermittent Fever | 1.12 (0.60-2.10) | 0.717 |  |  |
| Dizziness | 0.94 (0.48-1.84) | 0.862 |  |  |
| Tinnitus | 1.29 (0.67-2.49) | 0.442 |  |  |
| Diarrhoea | 0.82 (0.34-1.98) | 0.664 |  |  |
| Altered Sensation | 1.82 (0.81-4.08) | 0.145 |  |  |
| Abdominal Pain | 0.54 (0.13-2.30) | 0.401 |  |  |
| Depression | 1.25 (0.42-3.70) | 0.689 |  |  |
| **Number of Long Covid Symptoms** | 1.00 (0.93-1.08) | 0.960 |  |  |
| **Co-morbidity Status** | | | | |
| No | Ref | - |  |  |
| Yes | 1.24 (0.81-1.91) | 0.320 |  |  |
| **ORGANISATIONAL FACTORS** | | | | |
| **Experienced Work Adjustment** | | | | |
| Yes | Ref | - |  |  |
| No | 1.46 (0.76-2.83) | 0.257 |  |  |
| **Sickness Absence Policy at Workplace** | | | | |
| Yes | Ref | - |  |  |
| No | 0.93 (0.62-1.41) | 0.743 |  |  |
| **Experienced RTW Practice at Workplace** | | | | |
| Yes | Ref | - |  |  |
| No | 0.61 (0.33-1.12) | 0.113 |  |  |
| **PERSONAL FACTORS** | | | | |
| **Work Attitude** | | | | |
| Good | Ref | - |  |  |
| Poor | 0.95 (0.39-2.29) | 0.917 |  |  |
| **Employer Informed Regarding Long Covid Symptom** | | | | |
| No | Ref | - |  |  |
| Yes | 0.61 (0.34-1.11) | 0.104 |  |  |

*Determined via simple logistric regression

**^┼^** Determined via multiple logistic regression
