# Supplementary material for: Prevalence and risk factors of adverse work outcomes among manufacturing workers with Long COVID in Malaysia – A nationwide study
Source: PLoS One. 2026 Sep 11;21(9):e0357125. doi: 10.1371/journal.pone.0357125 (PMC13567834; doi:10.1371/journal.pone.0357125)
Supplement: Supplementary file 8 [file pone.0357125.s003.docx]

**S3 Table: Association between various risk factors and sickness presenteeism among manufacturing workers with Long COVID**

| **Risk Factors** | **Crude OR*** (**95% CI)** | **P-Value** | **Adjusted OR^┼^** (**95% CI)** | **P-Value** |
| --- | --- | --- | --- | --- |
| **SOCIO-CLINICAL FACTORS** | | | | |
| **Age (years old)** | 0.99 (0.98-1.01) | 0.462 |  |  |
| **Gender** | | | | |
| Male | Ref | - |  |  |
| Female | 1.16 (0.87-1.53) | 0.318 |  |  |
| **BMI Category (kg/m^2^)** | | | | |
| < 18.5 (underweight) | Ref | - |  |  |
| 18.5 – 24.9 (normal) | 0.94 (0.47-1.87) | 0.858 |  |  |
| 25.0 – 29.9 (overweight) | 1.02 (0.52-2.03) | 0.947 |  |  |
| > 30 (obese) | 1.12 (0.55-2.26) | 0.760 |  |  |
| **Smoking Status** | | | | |
| Non-smoker | Ref | - |  |  |
| Smoker | 0.95 (0.67-1.33) | 0.756 |  |  |
| **WORK FACTORS** | | | | |
| **Industrial Subsectors** | | | | |
| Machinery and Equipment | Ref | - |  |  |
| Chemical (including Petroleum) | 3.50 (0.24-51.89) | 0.363 |  |  |
| Electrical and Electronics | 1.04 (0.30-3.59) | 0.956 |  |  |
| Paper, Printing and Publishing | 2.63 (0.45-15.31) | 0.283 |  |  |
| Transport, Vehicle and Equipment | 2.21 (0.62-7.85) | 0.279 |  |  |
| Rubber | 1.41 (0.39-5.06) | 0.603 |  |  |
| Textile, Wearing Apparel and Leather | 1.01 (0.27-3.75) | 0.994 |  |  |
| **Working Departments** | | | | |
| Departments Requiring Lower Physical Activity | Ref | - |  |  |
| Departments Requiring Higher Physical Activity | 0.92 (0.61-1.37) | 0.669 |  |  |
| **Positions at Company** | | | | |
| Job Requiring Lower Physical Demand | Ref | - |  |  |
| Job Requiring High Physical Demand | 1.08 (0.81-1.44) | 0.596 |  |  |
| **Work Schedule** | | | | |
| Normal/Office hours | Ref | - | Ref | - |
| Shift including Staggered Shift | 1.36 (1.02-1.80) | **0.041*** | 1.25 (1.03-1.52) | **0.027*** |
| **Types of Hazards at Work** | | | | |
| VDU (Visual Display Unit) / Video (Computer) | 0.93 (0.66-1.30) | 0.646 |  |  |
| Manual Handling | 0.99 (0.72-1.37) | 0.949 |  |  |
| Excessive Noise | 0.85 (0.59-1.22) | 0.848 |  |  |
| Handling of Chemical Hazardous to Health | 1.00 (0.68-1.46) | 0.988 |  |  |
| Electrical Work | 1.00 (0.66-1.50) | 0.989 |  |  |
| Working in extreme temperatures (hot or cold) | 0.81 (0.48-1.35) | 0.412 |  |  |
| Confined Space Entry | 0.77 (0.45-1.33) | 0.354 |  |  |
| Working at Heights | 0.64 (0.30-1.37) | 0.251 |  |  |
| Driving Vehicle | 0.61 (0.27-1.38) | 0.286 |  |  |
| Lone / Isolated Work | 1.13 (0.55-2.35) | 0.737 |  |  |
| Driving Lift Truck | 1.23 (0.45-3.37) | 0.684 |  |  |
| Construction Work | 0.67 (0.27-1.67) | 0.391 |  |  |
| Working on Roofs | 2.65 (0.94-7.47) | 0.064 |  |  |
| Others | 0.59 (0.30-1.14) | 0.315 |  |  |
| **LONG COVID SYMPTOMS** | | | | |
| **Symptoms of Long Covid** | | | | |
| Post-exertional malaise | 1.28 (0.96-1.69) | 0.090 |  |  |
| Fatigue | 1.52 (1.14-2.03) | **0.005*** |  |  |
| Joint Pain | 0.99 (0.04-1.49) | 1.000 |  |  |
| Muscle pain | 0.98 (0.14-1.69) | 1.000 |  |  |
| Cough | 1.03 (0.52-1.99) | 0.988 |  |  |
| Headache | 1.31 (0.12-14.52) | 0.826 |  |  |
| Tingling | 0.67 (0.04-10.81) | 0.781 |  |  |
| Cognitive Dysfunction | 1.19 (0.82-1.72) | 0.365 |  |  |
| Dyspnoea | 0.98 (0.03-1.33) | 1.000 |  |  |
| Tachycardia | 1.34 (0.92-1.95) | 0.123 |  |  |
| Chest Pain | 2.02 (1.36-2.99) | **<0.001*** |  |  |
| Sleeping Disturbance | 0.59 (1.05-2.40) | **0.027*** |  |  |
| Intermittent Fever | 2.79 (1.78-4.37) | **<0.001*** |  |  |
| Dizziness | 1.02 (0.45-1.88) | 0.891 |  |  |
| Tinnitus | 2.45 (1.51-3.98) | **<0.001*** |  |  |
| Diarrhoea | 1.83 (1.05-3.18) | **0.032*** |  |  |
| Altered Sensation | 2.02 (1.05-3.89) | **0.035*** |  |  |
| Abdominal Pain | 2.38 (1.08-5.26) | **0.033*** |  |  |
| Depression | 1.37 (0.63-3.00) | 0.428 | Ref | - |
| **Number of Long Covid Symptoms** | 1.18 (1.12-1.24) | **<0.001*** | 1.15 (1.09-1.21) | **<0.001*** |
| **Co-morbidity Status** | | | | |
| No | Ref | - |  |  |
| Yes | 1.27 (0.95-1.71) | 0.112 |  |  |
| **ORGANISATIONAL FACTORS** | | | | |
| **Experienced Work Adjustment** | | | | |
| Yes | Ref | - |  |  |
| No | 1.49 (0.91-2.44) | 0.112 |  |  |
| **Sickness Absence Policy at Workplace** | | | | |
| Yes | Ref | - |  |  |
| No | 1.11 (0.84-1.47) | 0.478 |  |  |
| **Experienced RTW Practices at Workplace** | | | | |
| Yes | Ref | - |  |  |
| No | 0.40 (0.25-0.64) | **<0.001*** |  |  |
| **PERSONAL FACTORS** | | | | |
| **Work Attitude** | | | | |
| Good | Ref | - |  |  |
| Poor | 1.33 (0.74-2.37) | 0.349 |  |  |
| **Employer Informed Regarding Long Covid Symptom** | | | | |
| No | Ref | - | Ref | - |
| Yes | 0.59 (0.38-0.92) | **0.021*** | 0.58 (0.39-0.92) | **0.019*** |

*Determined via simple logistric regression

**^┼^** Determined via multiple logistic regression
