# Supplementary material for: Prevalence and risk factors of adverse work outcomes among manufacturing workers with Long COVID in Malaysia – A nationwide study
Source: PLoS One. 2026 Sep 11;21(9):e0357125. doi: 10.1371/journal.pone.0357125 (PMC13567834; doi:10.1371/journal.pone.0357125)
Supplement: Supplementary file 9 [file pone.0357125.s004.docx]

**S4 Table: Association between various risk factors and safety issues at work among manufacturing workers with Long COVID**

| **Risk Factors** | | **Crude OR***  **(95% CI)** | | **P-Value** | **Adjusted OR^┼^**  **(95% CI)** | **P-Value** |
| --- | --- | --- | --- | --- | --- | --- |
| **SOCIO-CLINICAL FACTORS** | | | | | | |
| **Age (years old)** | | 0.99 (0.96-1.02) | | 0.595 |  |  |
| **Gender** | | | | | | |
| Male | | Ref | | - |  |  |
| Female | | 0.70 (0.39-1.24) | | 0.217 |  |  |
| **BMI Category (kg/m^2^)** | | | | | | |
| -< 18.5 (underweight) | | Ref | | - |  |  |
| -18.5 – 24.9 (normal) | | 0.39 (0.12-1.27) | | 0.116 |  |  |
| -25.0 – 29.9 (overweight) | | 0.61 (0.20-1.88) | | 0.387 |  |  |
| > 30 (obese) | | 0.70 (0.22-2.27) | | 0.557 |  |  |
| **Smoking Status** | | | | | | |
| Non-smoker | | Ref | | - |  |  |
| Smoker | | 2.13 (1.17-3.90) | | **0.014*** |  |  |
| **WORK FACTORS** | | | | | | |
| **Industrial Subsectors** | | | | | | |
| Machinery and Equipment | | Ref | | - |  |  |
| Chemical (including Petroleum) | | 0.97 (0.05-1.65) | | 0.999 |  |  |
| Electrical and Electronics | | 0.98 (0.06-1.35) | | 0.889 |  |  |
| Paper, Printing and Publishing | | 2.01 (0.46-8.71) | | 0.353 |  |  |
| Transport, Vehicle and Equipment | | 4.00 (0.33-48.66) | | 0.277 |  |  |
| Rubber | | 3.92 (0.88-17.50) | | 0.274 |  |  |
| Textile, Wearing Apparel and Leather | | 2.95 (0.62-14.03) | | 0.275 |  |  |
| **Working Departments** | | | | | | |
| Departments Requiring Lower Physical Activity | | Ref | | - |  |  |
| Departments Requiring Higher Physical Activity | | 0.86 (0.39-1.88) | | 0.703 |  |  |
| **Positions at Company** | | | | | | |
| Job Requiring Lower Physical Demand | | Ref | | - |  |  |
| Job Requiring High Physical Demand | | 0.62 (0.34-1.15) | | 0.127 |  |  |
| **Work Schedule** | | | | | | |
| Normal/Office hours | | Ref | | - |  |  |
| Shift including Staggered Shift | | 1.30 (0.73-2.31) | | 0.382 |  |  |
| **Types of Hazards at Work*^┼^*** | | | | | | |
| VDU (Visual Display Unit) / Video (Computer) | | 0.99 (0.55-1.79) | | 0.981 |  |  |
| Manual Handling | | 1.13 (0.63-2.04) | | 0.680 |  |  |
| Excessive Noise | | 0.55 (0.31-0.99) | | **0.048*** |  |  |
| Handling of Chemical Hazardous to Health | | 0.46 (0.25-0.82) | | **0.008*** | 2.51 (1.60-3.94) | **<0.001*** |
| Electrical Work | | 0.69 (0.36-1.32) | | 0.260 |  |  |
| Working in extreme temperatures (hot or cold) | | 0.42 (0.21-0.83) | | **0.013*** |  |  |
| Confined Space Entry | | 0.49 (0.23-1.01) | | 0.053 |  |  |
| Working at Heights | | 0.47 (0.20-1.09) | | 0.080 |  |  |
| Driving Vehicle | | 1.11 (0.26-4.76) | | 0.889 |  |  |
| Lone / Isolated Work | | 0.40 (0.16-1.01) | | 0.051 |  |  |
| Driving Lift Truck | | 1.84 (0.25-13.81) | | 0.554 |  |  |
| Construction Work | | 0.31 (0.11-0.85) | | **0.023*** |  |  |
| Working on Roofs | | 0.35 (0.13-0.95) | | **0.040*** |  |  |
| Others | | 0.41 (0.15-1.11) | | 0.081 |  |  |
| **LONG COVID SYMPTOMS** | | | | | | |
| **Symptoms of Long Covid** | | | | | | |
| Post-exertional malaise | 1.36 (0.76-2.42) | | 0.304 | |  |  |
| Fatigue | 1.60 (0.90-2.85) | | 0.108 | |  |  |
| Joint Pain | 0.85 (0.29-4.43) | | 0.977 | |  |  |
| Muscle pain | 0.90 (0.48-2.25) | | 0.899 | |  |  |
| Cough | 0.88 (0.14-1.69) | | 1.000 | |  |  |
| Headache | 1.03 (0.78-7.64) | | 0.999 | |  |  |
| Tingling | 0.82 (0.29-9.94) | | 1.000 | |  |  |
| Cognitive Dysfunction | 1.08 (0.51-2.28) | | 0.836 | |  |  |
| Dyspnoea | 0.98 (0.59-9.96) | | 1.000 | |  |  |
| Tachycardia | 2.31 (1.22-4.36) | | **0.010*** | |  |  |
| Chest Pain | 2.13 (1.09-4.13) | | **0.026*** | |  |  |
| Sleeping Disturbance | 1.72 (0.83-3.55) | | 0.145 | |  |  |
| Intermittent Fever | 2.00 (0.96-4.15) | | 0.063 | |  |  |
| Dizziness | 2.71 (1.36-5.40) | | **0.005*** | |  |  |
| Tinnitus | 1.30 (0.54-3.15) | | 0.564 | |  |  |
| Diarrhoea | 1.94 (0.79-4.78) | | 0.149 | |  |  |
| Altered Sensation | 2.95 (1.17-7.42) | | **0.021*** | |  |  |
| Abdominal Pain | 4.71 (1.81-12.28) | | **0.001*** | |  |  |
| Depression | 2.01 (0.58-6.94) | | 0.270 | |  |  |
| **Number of Long Covid Symptoms (times)** | 1.17 (1.08-1.26) | | **<0.001*** | |  |  |
| **Co-morbidity Status** | | | | | | |
| No | Ref | | - | |  |  |
| Yes | 1.88 (1.06-3.34) | | **0.032*** | |  |  |
| **ORGANISATIONAL FACTORS** | | | | | | |
| **Work Adjustment Experience** | | | | | | |
| Yes | Ref | | - | |  |  |
| No | 1.17 (0.45-3.04) | | 0.754 | |  |  |
| **Sickness Absence Policy** | | | | |  |  |
| Yes | Ref | | - | |  |  |
| No | 1.26 (0.71-2.25) | | 0.432 | |  |  |
| **RTW Practice at Workplace** | | | | |  |  |
| Yes | Ref | | - | |  |  |
| No | 0.54 (0.23-1.28) | | 0.162 | |  |  |
| **PERSONAL FACTORS** | | | | | | |
| **Work Attitude** | | | | | | |
| Good | Ref | | - | |  |  |
| Poor | 1.36 (0.47-3.94) | | 0.589 | |  |  |
| **Employer Informed Regarding Long Covid Symptom** | | | | | | |
| No | Ref | | - | | Ref | - |
| Yes | 0.61 (0.27-1.34) | | 0.218 | | 0.32 (0.14-0.74) | **0.007*** |

*Determined via simple logistric regression

**^┼^** Determined via multiple logistic regression
