# Supplementary material for: Prevalence and risk factors of adverse work outcomes among manufacturing workers with Long COVID in Malaysia – A nationwide study
Source: PLoS One. 2026 Sep 11;21(9):e0357125. doi: 10.1371/journal.pone.0357125 (PMC13567834; doi:10.1371/journal.pone.0357125)
Supplement: Supplementary file 10 [file pone.0357125.s005.docx]

**S5 Table: Moderating Effects in the Association Between Long Covid and Adverse Work Outcomes**

| **Interactions** | **Adjusted OR (95% CI)** | **P-Value** |
| --- | --- | --- |
| **Moderating Effects in the Association Between Long COVID and WRQOL** | | |
| Long COVID x Work Adjustment Experience | 0.75 (0.46-1.24) | 0.264 |
| Long COVID x Sickness Absence Policy at Workplace | 1.03 (0.62-1.70) | 0.910 |
| Long COVID x Return-to-Work Practice at Workplace | 0.30 (0.34-1.18) | 0.629 |
| Long COVID x Work Attitude | 1.02 (0.45-2.32) | 0.967 |
| Long COVID x Informed Employer on Long COVID | 0.92 (0.30-2.70) | 0.854 |
| **Moderating Effects in the Association Between Long COVID and Sickness Absenteeism** | | |
| Long COVID x Work Adjustment Experience | 1.24 (0.72-2.14) | 0.442 |
| Long COVID x Sickness Absence Policy at Workplace | 1.01 (0.59-1.74) | 0.968 |
| Long COVID x Return-to-Work Practice at Workplace | 1.36 (0.78-2.39) | 0.278 |
| Long COVID x Work Attitude | 1.14 (0.38-3.36) | 0.818 |
| Long COVID x Informed Employer on Long COVID | 0.84 (0.33-2.10) | 0.702 |
| **Moderating Effects in the Association Between Long COVID and Sickness Presenteeism** | | |
| Long COVID x Work Adjustment Experience | 1.00 (0.68-1.48) | 0.983 |
| Long COVID x Sickness Absence Policy at Workplace | 1.02 (0.69-1.50) | 0.921 |
| Long COVID x Return-to-Work Practice at Workplace | 1.13 (0.76-1.69) | 0.546 |
| Long COVID x Work Attitude | 0.75 (0.35-1.59) | 0.453 |
| Long COVID x Informed Employer on Long COVID | 0.81 (0.41-1.61) | 0.553 |
| **Moderating Effects in the Association Between Long COVID and Safety Issues at Work** | | |
| Long COVID x Work Adjustment Experience | 0.52 (0.11-2.38) | 0.396 |
| Long COVID x Sickness Absence Policy at Workplace | 0.40 (0.13-1.25) | 0.115 |
| Long COVID x Return-to-Work Practice at Workplace | 1.03 (0.66-1.79) | 0.442 |
| Long COVID x Work Attitude | 0.25 (0.03-2.27) | 0.216 |
| Long COVID x Informed Employer on Long COVID | 0.36 (0.10-1.31) | 0.121 |
