# Supplementary material for: Prevalence and risk factors of adverse work outcomes among manufacturing workers with Long COVID in Malaysia – A nationwide study
Source: PLoS One. 2026 Sep 11;21(9):e0357125. doi: 10.1371/journal.pone.0357125 (PMC13567834; doi:10.1371/journal.pone.0357125)
Supplement: Supplementary file 12 [file pone.0357125.s012.docx]

**Response to Reviewer**

We sincerely thank the reviewer for this insightful comment, which has helped improve the clarity of the manuscript.

1. Not sure why this is here since all subjects have long COVID. Is it the symptoms?

**RESPONSE:** Thank you for highlighting this issue. Indeed, all participants had Long COVID. To avoid confusion, the phrase has been revised to “Long COVID symptoms” to clarify that the risk factors examined included both the number of Long COVID symptoms and the presence of specific symptoms.

**Other grammatical errors and problematic phrases have been revised accordingly.**
