# Supplementary material for: Prevalence and risk factors of adverse work outcomes among manufacturing workers with Long COVID in Malaysia – A nationwide study
Source: PLoS One. 2026 Sep 11;21(9):e0357125. doi: 10.1371/journal.pone.0357125 (PMC13567834; doi:10.1371/journal.pone.0357125)
Supplement: Peer review history

# Peer review: Decision Letter 0

**DOI:** 10.1371/journal.pone.0357125.r001

**Authors:** Shairyzah Ahmad Hisham

5 Apr 2026

Dear Dr. Yew,

Thank you for submitting your manuscript to PLOS ONE. After careful consideration, we feel that it has merit but does not fully meet PLOS ONE’s publication criteria as it currently stands. Therefore, we invite you to submit a revised version of the manuscript that addresses the points raised during the review process.

Please review the comments from the two reviewers below and in the attached files and incorporate the necessary amendments to improve the scientific rigor of the manuscript, particularly the methodology and results session.

Please submit your revised manuscript by May 20 2026 11:59PM. If you will need more time than this to complete your revisions, please reply to this message or contact the journal office at plosone@plos.org. When you're ready to submit your revision, log on to https://www.editorialmanager.com/pone/ and select the 'Submissions Needing Revision' folder to locate your manuscript file.

- A letter that responds to each point raised by the academic editor and reviewer(s). You should upload this letter as a separate file labeled 'Response to Reviewers'.
- A marked-up copy of your manuscript that highlights changes made to the original version. You should upload this as a separate file labeled 'Revised Manuscript with Track Changes'.
- An unmarked version of your revised paper without tracked changes. You should upload this as a separate file labeled 'Manuscript'.

If you would like to make changes to your financial disclosure, please include your updated statement in your cover letter. Guidelines for resubmitting your figure files are available below the reviewer comments at the end of this letter.

If applicable, we recommend that you deposit your laboratory protocols in protocols.io to enhance the reproducibility of your results. Protocols.io assigns your protocol its own identifier (DOI) so that it can be cited independently in the future. For instructions see: https://journals.plos.org/plosone/s/submission-guidelines#loc-laboratory-protocols. Additionally, PLOS ONE offers an option for publishing peer-reviewed Lab Protocol articles, which describe protocols hosted on protocols.io. Read more information on sharing protocols at https://plos.org/protocols?utm_medium=editorial-email&utm_source=authorletters&utm_campaign=protocols.

As the corresponding author, your ORCID iD is verified in the submission system and will appear in the published article. PLOS supports the use of ORCID, and we encourage all coauthors to register for an ORCID iD and use it as well. Please encourage your coauthors to verify their ORCID iD within the submission system before final acceptance, as unverified ORCID iDs will not appear in the published article. *Only*  the individual author can complete the verification step; PLOS staff *cannot*  verify ORCID iDs on behalf of authors.

We look forward to receiving your revised manuscript.

Kind regards,

Shairyzah Ahmad Hisham, PhD.

Academic Editor

PLOS One

Journal Requirements:

When submitting your revision, we need you to address these additional requirements.

1. Please ensure that your manuscript meets PLOS ONE's style requirements, including those for file naming. The PLOS ONE style templates can be found at

https://journals.plos.org/plosone/s/file?id=wjVg/PLOSOne_formatting_sample_main_body.pdf and

https://journals.plos.org/plosone/s/file?id=ba62/PLOSOne_formatting_sample_title_authors_affiliations.pdf

2. Thank you for stating the following financial disclosure:

“This work was supported by the National Institute of Occupational Safety and Health (NIOSH) Malaysia [grant number: UKMP-S230424] to [HMY]. “

Please state what role the funders took in the study. If the funders had no role, please state: "The funders had no role in study design, data collection and analysis, decision to publish, or preparation of the manuscript."

If this statement is not correct you must amend it as needed.

Please include this amended Role of Funder statement in your cover letter; we will change the online submission form on your behalf.

3. We note that you have indicated that there are restrictions to data sharing for this study. For studies involving human research participant data or other sensitive data, we encourage authors to share de-identified or anonymized data. However, when data cannot be publicly shared for ethical reasons, we allow authors to make their data sets available upon request. For information on unacceptable data access restrictions, please see http://journals.plos.org/plosone/s/data-availability#loc-unacceptable-data-access-restrictions.

Before we proceed with your manuscript, please address the following prompts:

a) If there are ethical or legal restrictions on sharing a de-identified data set, please explain them in detail (e.g., data contain potentially identifying or sensitive patient information, data are owned by a third-party organization, etc.) and who has imposed them (e.g., a Research Ethics Committee or Institutional Review Board, etc.). Please also provide contact information for a data access committee, ethics committee, or other institutional body to which data requests may be sent.

b) If there are no restrictions, please upload the minimal anonymized data set necessary to replicate your study findings to a stable, public repository and provide us with the relevant URLs, DOIs, or accession numbers. Please see http://www.bmj.com/content/340/bmj.c181.long for guidelines on how to de-identify and prepare clinical data for publication. For a list of recommended repositories, please see https://journals.plos.org/plosone/s/recommended-repositories. You also have the option of uploading the data as Supporting Information files, but we would recommend depositing data directly to a data repository if possible.

Please update your Data Availability statement in the submission form accordingly.

4. Please include captions for your Supporting Information files at the end of your manuscript, and update any in-text citations to match accordingly. Please see our Supporting Information guidelines for more information: http://journals.plos.org/plosone/s/supporting-information.

5. If the reviewer comments include a recommendation to cite specific previously published works, please review and evaluate these publications to determine whether they are relevant and should be cited. There is no requirement to cite these works unless the editor has indicated otherwise.

[Note: HTML markup is below. Please do not edit.]

Reviewers' comments:

Reviewer's Responses to Questions

**Comments to the Author**

1. Is the manuscript technically sound, and do the data support the conclusions?

Reviewer #1: Partly

Reviewer #2: Partly

**********

2. Has the statistical analysis been performed appropriately and rigorously?

Reviewer #1: Yes

Reviewer #2: I Don't Know

**********

3. Have the authors made all data underlying the findings in their manuscript fully available?

The PLOS Data policy

Reviewer #1: Yes

Reviewer #2: No

**********

4. Is the manuscript presented in an intelligible fashion and written in standard English?

Reviewer #1: Yes

Reviewer #2: No

**********

Reviewer #1: Dear authors,

Overall, the manuscript was written quite well. There are, however, a few sections of the manuscript that, in my opinion, could be further improved to enhance their clarity. Please kindly refer to the attached document on suggestions for you to consider.

Thank you.

Reviewer #2: The manuscript addresses an insightful topic. Nevertheless, several issues require clarification and improvement.

Scope of study: The study implies nationwide coverage (e.g. "entire nation", “across all states”) but there's lack in clarity regarding geographical precision; the distribution and number of participating manufacturing companies are not explicitly reported. The sampling strategy and data collection procedures are not sufficiently detailed giving an impression of vague representation.

Enrolment criteria: The inclusion and exclusion criteria set may not be sufficiently comprehensive and leaving gaps in ruling out confounding symptoms or pre-existing conditions that could overlap with or mimic Long COVID. More explicit criteria are necessary to strengthen internal validity and reduce misclassification bias.

Survey instruments: The use of established, validated instruments to assess outcomes is a strength and contributes to methodological rigour. However, the development and application of the Long COVID instrument are not sufficiently explained. Reporting of "prevalence" of symptoms and adverse outcomes only accounted for workers alleged LC symptoms. Reporting of subjective parameters remains vague, raising analytical and interpretative concerns regarding measurement validity and comparability.

Study design limitations and interpretation of findings: The limitations inherent to the cross-sectional design are not adequately addressed. Greater transparency is needed regarding the structure and content of the survey tools, particularly in relation to several paradoxical or counterintuitive findings (e.g. high WRQoL despite symptom burden), which warrant further methodological and analytical clarification.

Conceptual framework and conclusions: The scope of the study and its underlying conceptual framework are not clearly articulated. Several claims and conclusions may require revision or qualification in view of the study design and sampling approach.

Language and presentation: Refinement of language and expression is recommended for reporting and scientific writing. Improvements in clarity, coherence, and grammatical precision would enhance the overall readability of the manuscript.

**********

what does this mean?). If published, this will include your full peer review and any attached files.

If you choose “no”, your identity will remain anonymous but your review may still be made public.

**Do you want your identity to be public for this peer review?**  For information about this choice, including consent withdrawal, please see our Privacy Policy

Reviewer #1: **Yes:** Zainol Akbar Zainal

Reviewer #2: No

**********

[NOTE: If reviewer comments were submitted as an attachment file, they will be attached to this email and accessible via the submission site. Please log into your account, locate the manuscript record, and check for the action link "View Attachments". If this link does not appear, there are no attachment files.]

To ensure your figures meet our technical requirements, please review our figure guidelines: https://journals.plos.org/plosone/s/figures

You may also use PLOS’s free figure tool, NAAS, to help you prepare publication quality figures: https://journals.plos.org/plosone/s/figures#loc-tools-for-figure-preparation.

NAAS will assess whether your figures meet our technical requirements by comparing each figure against our figure specifications.

Attachment Submitted filename: PONE-D-25-68765 - Reviewer Comments.pdf

Attachment Submitted filename: PONE-D-25-68765 rev1.pdf

# Author comment: Author response to Decision Letter 1

**DOI:** 10.1371/journal.pone.0357125.r002

9 Apr 2026

We would like to thank the reviewer for the careful evaluation of our manuscript and for the constructive and insightful comments provided. We have carefully considered all suggestions and have revised the manuscript accordingly to improve its clarity, methodological transparency, and overall robustness, as follows.

RESPONSES TO EDITOR

1. Please ensure that your manuscript meets PLOS ONE's style requirements, including those for file naming.

RESPONSE: The manuscript has been revised accordingly based on PLOS ONE’s style requirement.

2. Thank you for stating the following financial disclosure: “This work was supported by the National Institute of Occupational Safety and Health (NIOSH) Malaysia [grant number: UKMP-S230424] to [HMY]. Please state what role the funders took in the study. If the funders had no role, please state: “The funders had no role in study design, data collection and analysis, decision to publish, or preparation of the manuscript”. If this statement is not correct you must amend it as needed. Please include this amended Role of Funder statement in your cover letter; we will change the online submission form on your behalf.

RESPONSE: Thank you for the reminded. Indeed, the funder (NIOSH Malaysia) had no role in study design, data collection and analysis, decision to publish, or preparation of the manuscript. This statement has been added to the manuscript and the cover letter.

3. We note that you have indicated that there are restrictions to data sharing for this study. For studies involving human research participant data or other sensitive data, we encourage authors to share de-identified or anonymized data. However, when data cannot be publicly shared for ethical reasons, we allow authors to make their data sets available upon request. For information on unacceptable data access restrictions, please see http://journals.plos.org/plosone/s/data-availability#loc-unacceptable-data-access-restrictions. Before we proceed with your manuscript, please address the following prompts:

• If there are ethical or legal restrictions on sharing a de-identified data set, please explain them in detail (e.g., data contain potentially identifying or sensitive patient information, data are owned by a third-party organization, etc.) and who has imposed them (e.g., a Research Ethics Committee or Institutional Review Board, etc.). Please also provide contact information for a data access committee, ethics committee, or other institutional body to which data requests may be sent.

• If there are no restrictions, please upload the minimal anonymized data set necessary to replicate your study findings to a stable, public repository and provide us with the relevant URLs, DOIs, or accession numbers. Please see http://www.bmj.com/content/340/bmj.c181.long for guidelines on how to de-identify and prepare clinical data for publication. For a list of recommended repositories, please see https://journals.plos.org/plosone/s/recommended-repositories. You also have the option of uploading the data as Supporting Information files, but we would recommend depositing data directly to a data repository if possible.

RESPONSE: Regrettably, the Long COVID dataset cannot be shared publicly primarily due to concerns around patient privacy, legal regulations, and ethical approvals. Even when personal identifiers are removed, individuals may still be re-identified from combined data (e.g., working department, position at company, work schedule, etc), especially in workplace with small number of workers. Additionally, the Personal Data Protection Act 2010 restricts the sharing of sensitive health information. Furthermore, the ethics committee limits data use to research purposes, and public sharing may violate the conditions under which participants originally consented. This explanation is provided at the availability of data and materials section.

4. Please include captions for your Supporting Information files at the end of your manuscript, and update any in-text citations to match accordingly. Please see our Supporting Information guidelines for more information: http://journals.plos.org/plosone/s/supporting-information.

RESPONSE: The captions of all supporting information files has been added to the end of the manuscript, and the in-text citations have been revised accordingly.

RESPONSES TO REVIEWER 1

1. Introduction (abstract): The two sentences seem to contradict each other. The first statement mentions a high prevalence of Long COVID, while the second indicates that the study’s goal was to determine the prevalence. Consider rephrasing. Consider highlighting the knowledge gap and problem statement in this section.

RESPONSE: Thank you for highlighting the blind spot. To better illustrate the research gap and the justifications of the present study, the introduction of the abstract has been revised to: “The high prevalence and multiorgan involvement of Long COVID among the general population has raised concerns about the possible long-term effect of this condition among industrial workers”. It was the observation of the high prevalence of Long COVID among the general population that inspired the authors to conduct a study specifically among the manufacturing workers.

2. Materials and methods (abstract): The instruments used are mentioned but the methods used to distribute the instruments are not. Please state accordingly. Consider moving the total number of respondents (i.e., 797) to the results section. Consider adding the inclusion and exclusion criteria of participants. Line 52 – to correct the spelling of the word ‘utilised’.

RESPONSE: The methods of distributing the questionnaires was added to the materials and methods section of the abstract. Due to the word limit of the abstract, we could only add a brief description of the inclusion criteria of the participants. The total number of participants has been moved to the result section as advised and spelling error has been corrected.

3. Results (abstract): For better clarity, consider restructuring the first part of the results section. I would suggest starting with the number of respondents, followed by prevalence and so on. Line 59-60: There is a repetition of information i.e., 42.4%. Consider rephrasing. Regarding the reported risk factors, consider reporting key effect sizes (e.g., adjusted odds ratios) with 95% confidence intervals to allow readers to interpret the magnitude of the associations.

RESPONSE: The number of participants has been added. The repetition of 42.4% was removed. The aOR for all associated risk factors were included.

4. Conclusion (abstract): The conclusion mostly repeats the results and is quite general. Consider making it stronger by emphasizing the main implications of the findings for occupational health practices or workplace policies that support workers dealing with Long COVID. This would give readers a clearer idea of the importance of the study.

RESPONSE: Due to the word limits in abstract, we are unable to extensively elaborate on the impact of the study. However, we concisely emphasised the study implications as follows: “The impact of Long COVID on WRQoL, sickness presenteeism and sickness absenteeism, as well as the multifactorial nature of adverse work outcomes highlights the need for targeted workplace interventions, supportive policies, early detection, and inclusive occupational health frameworks at the workplace”.

5. Study design: I would recommend using the phrase ‘cross-sectional survey’ instead.

RESPONSE: The authors agree that the phrase “cross-sectional survey” is a better description than “cross-sectional study”. Hence, it has been revised accordingly.

6. Data collection protocol: The protocol was described but could be further elaborated. Consider including this information for better clarity:

• Any method used to select the manufacturing companies

• The individual that the researcher reached out to provide the list.

• Were similar data collection protocols used at all companies visited.

• The number of researchers present at the site (if relevant).

• The kind of assistance provided during the data collection e.g., explanation on medical terms such as tachycardia, tinnitus etc.

Consider incorporating the specific types of demographic data gathered in this section. Furthermore, it would be beneficial to indicate the methods used for data collection, such as whether body weight and height were self-reported or measured.

RESPONSE: Initially, manufacturing companies listed in the Federation of Malaysian Manufacturers (FMM) directory that met the eligibility criteria were selected using a multistage sampling approach. However, due to a very low response rate (e.g., companies declining participation), the researchers subsequently adopted a convenience sampling method. Under this approach, any company that provided consent to participate from each zone in Malaysia was included. After obtaining informed consent from the company, the human resource personnel were contacted to provide a list of workers (i.e., the sampling frame). A standardised data collection protocol was applied across all participating companies. During data collection, a team of 2–5 researchers/enumerators (previously trained using the standardised protocol) was present at each site to conduct researcher-assisted interviews while administering the questionnaire, including clarifying items and explaining complex medical terminology. Sociodemographic information, including age, gender, BMI (from self-reported weight and height), and smoking status, was collected.

7. Ethical approval: It is unclear why the study requires MREC approval. To clarify. Consider indicating if any authorisation was provided from the manufacturing companies to visit and conduct data collection.

RESPONSE: The study applied for MREC ethics approval as some of the participants were healthcare professionals working at the Ministry of Health, Malaysia. Written/electronic consent was also obtained from the respective manufacturing companies prior to study collection. Such clarification has been included to the materials and methods section.

RESPONSES TO REVIEWER 2

1. Scope of study: The study implies nationwide coverage (e.g. “entire nation”, “across all states”) but there’s lack in clarity regarding geographical precision; the distribution and number of participating manufacturing companies are not explicitly reported. The sampling strategy and data collection procedures are not sufficiently detailed giving an impression of vague representation.

RESPONSE: A cross-sectional survey was conducted across Malaysia’s five geographical zones (i.e., East Coast, Northern, Southern, Central, and East Malaysia). Initially, a multistage sampling strategy was planned for the selection of manufacturing companies, involving cluster sampling within each geographical zone based on the total number of companies, followed by proportionate stratified sampling to select workers from the states within each zone. However, due to a very low response rate (e.g., companies declining participation), the researchers opted to use a convenience sampling method instead. This resulted in three, four, three, six, and two companies participated across East Coast, Northern, Southern, Central, and East Malaysia, respectively. Under this approach, any company that provided consent to participate from each zone in Malaysia was included. This was followed by a universal sampling to select workers from each factory.

2. Enrolment criteria: The inclusion and exclusion criteria set may not be sufficiently comprehensive and leaving gaps in ruling out confounding symptoms or pre-existing conditions that could overlap with or mimic Long COVID. More explicit criteria are necessary to strengthen internal validity and reduce misclassification bias.

RESPONSE: We acknowledge that the initial inclusion and exclusion criteria may not have fully accounted for potential confounding symptoms or pre-existing conditions that could overlap with or mimic Long COVID, such as bronchial asthma and chronic obstructive pulmonary disease. To address this, several measures were incorporated into the study design and data collection process. Firstly, information on pre-existing comorbidities (e.g., respiratory, cardiovascular, and metabolic conditions) was collected and participants included in the multivariate analysis to allow adjustment for potential confounders (as reported in appendix). Secondly, participants were required to have a confirmed diagnosis of COVID-19 within a defined time frame (i.e., fully fulling the definition of WHO), to ensure temporal relevance to Long COVID assessment. Thirdly, symptoms were assessed in relation to their onset following COVID-19 infection, which helped differentiate new or persistent symptoms from pre-existing conditions. Such discussion has been added to the discussion section.

3. Survey instruments: The use of established, validated instruments to assess outcomes is a strength and contributes to methodological rigour. However, the development and application of the Long COVID instrument are not sufficiently explained. Reporting of “prevalence” of symptoms and adverse outcomes only accounted for workers alleged LC symptoms. Reporting of subjective parameters remains vague, raising analytical and interpretative concerns regarding measurement validity and comparability.

RESPONSE: Our apology for the blind spot. The development and validation process of the Long COVID questionnaire has been reported in our previously published study protocol, available at: Yusoff HM, Mohamed NC, Yew SQ, Nawi AM, Rashidah Ismail OH, Tohit NM, et al. Prevalence and risk factors of long covid and its associated adverse work outcomes among workers in the manufacturing sector in Malaysia – a mixed-methods study protocol. J Health Popul Nutr. 2024 Nov 25;43(1):194. doi:10.1186/s41043-024-00696-z. Meanwhile, the psychometric properties of the Long COVID questionnaire is still in press. This clarification has been added to the materials and method section.

4. Study design limitations and interpretation of findings: The limitations inherent to the cross-sectional design are not adequately addressed. Greater transparency is needed regarding the structure and content of the survey tools, particularly in relation to several paradoxical or counterintuitive findings (e.g. high WRQoL despite symptom burden), which warrant further methodological and analytical clarification.

RESPONSE: To enhance transparency regarding the structure and content of the survey tools, the development and validation process of the Long COVID questionnaire is described in the study protocol (reference added to the manuscript). However, the questionnaire cannot be made publicly available as it is copyrighted by the sponsor (NIOSH Malaysia). With regard to the seemingly paradoxical finding of relatively high WRQoL despite the presence of Long COVID symptoms, several explanations are possible. Firstly, sufficient coping strategies among workers may mitigate the perceived impact of persistent symptoms on WRQoL. For instance, work-focused rehabilitation, multidisciplinary inpatient and outpatient rehabilitation, psychoeducation, pacing, and breathing strategies, have shown to shift focus from symptom monitoring to optimising functional outcomes. These strategies allowed workers affected by Long COVID to maintain good WRQoL. Secondly, many participants may have experienced mild or manageable symptoms (as most participants only experience one symptom) that did not substantially impair their work functioning, as observed in the low proportion of workers with sickness absenteeism, sickness presenteeism, and safety issues at work. These points have now been discussed in the revised discussion section to provide clearer interpretation of the findings.

5. Conceptual framework and conclusions: The scope of the study and its underlying conceptual framework are not clearly articulated. Several claims and conclusions may require revision or qualification in view of the study design and sampling approach.

RESPONSE: The study is based on the premise that Long COVID workers may experience adverse work outcomes, which is associated with socio

Attachment Submitted filename: Responses to Editor and Reviewers.docx

# Peer review: Decision Letter 1

**DOI:** 10.1371/journal.pone.0357125.r003

**Authors:** Shairyzah Ahmad Hisham

25 May 2026

Dear Dr. Yew,

Thank you for submitting your manuscript to PLOS ONE. After careful consideration, we feel that it has merit but does not fully meet PLOS ONE’s publication criteria as it currently stands. Therefore, we invite you to submit a revised version of the manuscript that addresses the points raised during the review process.

Please refer to the reviewers' comments and make the necessary amendments. It is also strongly advisable for the manuscript to undergo thorough proofreading to eliminate spelling and grammatical errors and to ensure that clear, precise, and academically appropriate language is consistently used throughout, thereby enhancing the overall clarity and quality of the manuscript.

Please submit your revised manuscript by Jul 09 2026 11:59PM. If you will need more time than this to complete your revisions, please reply to this message or contact the journal office at plosone@plos.org. When you're ready to submit your revision, log on to https://www.editorialmanager.com/pone/ and select the 'Submissions Needing Revision' folder to locate your manuscript file.

- A letter that responds to each point raised by the academic editor and reviewer(s). You should upload this letter as a separate file labeled 'Response to Reviewers'.
- A marked-up copy of your manuscript that highlights changes made to the original version. You should upload this as a separate file labeled 'Revised Manuscript with Track Changes'.
- An unmarked version of your revised paper without tracked changes. You should upload this as a separate file labeled 'Manuscript'.

If you would like to make changes to your financial disclosure, please include your updated statement in your cover letter. Guidelines for resubmitting your figure files are available below the reviewer comments at the end of this letter.

If applicable, we recommend that you deposit your laboratory protocols in protocols.io to enhance the reproducibility of your results. Protocols.io assigns your protocol its own identifier (DOI) so that it can be cited independently in the future. For instructions see: https://journals.plos.org/plosone/s/submission-guidelines#loc-laboratory-protocols. Additionally, PLOS ONE offers an option for publishing peer-reviewed Lab Protocol articles, which describe protocols hosted on protocols.io. Read more information on sharing protocols at https://plos.org/protocols?utm_medium=editorial-email&utm_source=authorletters&utm_campaign=protocols.

As the corresponding author, your ORCID iD is verified in the submission system and will appear in the published article. PLOS supports the use of ORCID, and we encourage all coauthors to register for an ORCID iD and use it as well. Please encourage your coauthors to verify their ORCID iD within the submission system before final acceptance, as unverified ORCID iDs will not appear in the published article. *Only*  the individual author can complete the verification step; PLOS staff *cannot*  verify ORCID iDs on behalf of authors.

We look forward to receiving your revised manuscript.

Kind regards,

Shairyzah Ahmad Hisham, PhD.

Academic Editor

PLOS One

Journal Requirements:

If the reviewer comments include a recommendation to cite specific previously published works, please review and evaluate these publications to determine whether they are relevant and should be cited. There is no requirement to cite these works unless the editor has indicated otherwise.

Please review your reference list to ensure that it is complete and correct. If you have cited papers that have been retracted, please include the rationale for doing so in the manuscript text, or remove these references and replace them with relevant current references. Any changes to the reference list should be mentioned in the rebuttal letter that accompanies your revised manuscript. If you need to cite a retracted article, indicate the article’s retracted status in the References list and also include a citation and full reference for the retraction notice.

[Note: HTML markup is below. Please do not edit.]

Reviewers' comments:

Reviewer's Responses to Questions

**Comments to the Author**

Reviewer #1: (No Response)

Reviewer #2: All comments have been addressed

**********

2. Is the manuscript technically sound, and do the data support the conclusions?

Reviewer #1: Yes

Reviewer #2: Yes

**********

3. Has the statistical analysis been performed appropriately and rigorously?

Reviewer #1: Yes

Reviewer #2: I Don't Know

**********

4. Have the authors made all data underlying the findings in their manuscript fully available?

The PLOS Data policy

Reviewer #1: Yes

Reviewer #2: No

**********

5. Is the manuscript presented in an intelligible fashion and written in standard English?

Reviewer #1: Yes

Reviewer #2: Yes

**********

Reviewer #1: Dear authors,

I appreciate your consideration of the majority of my earlier recommendations for enhancing the original manuscript. I would like to emphasize that there were several additional suggestions that were not addressed in the revised manuscript or may have been overlooked, as they were not mentioned in the Response to Reviewers section.

Thank you.

Reviewer #2: Core concerns mostly addressed - with improved clarity, methodological transparency, a clearer conceptual framework and analytical clarity, and coherent interpretation of paradoxical findings.

Several issues require clarification or refinement.

Limitations due to recruitment, selection bias (convenience sampling); in addition to the risk of recall bias, potential misclassification bias should be discussed (if the questionnaire-based algorithm lack clinical validation). Revise wording that may imply overinterpretation or strong claims.

Others: proofreading to improve language and grammar throughout, ensure consistency of terminology and review clarity of table titles, labels/captions.

**********

what does this mean?). If published, this will include your full peer review and any attached files.

If you choose “no”, your identity will remain anonymous but your review may still be made public.

**Do you want your identity to be public for this peer review?**  For information about this choice, including consent withdrawal, please see our Privacy Policy

Reviewer #1: **Yes:** Zainol Akbar Zainal

Reviewer #2: No

**********

[NOTE: If reviewer comments were submitted as an attachment file, they will be attached to this email and accessible via the submission site. Please log into your account, locate the manuscript record, and check for the action link "View Attachments". If this link does not appear, there are no attachment files.]

To ensure your figures meet our technical requirements, please review our figure guidelines: https://journals.plos.org/plosone/s/figures

You may also use PLOS’s free figure tool, NAAS, to help you prepare publication quality figures: https://journals.plos.org/plosone/s/figures#loc-tools-for-figure-preparation.

NAAS will assess whether your figures meet our technical requirements by comparing each figure against our figure specifications.

Attachment Submitted filename: PONE-D-25-68765_R1 - Reviewer Comments.pdf

# Author comment: Author response to Decision Letter 2

**DOI:** 10.1371/journal.pone.0357125.r004

9 Jun 2026

We would like to thank the reviewer for the careful evaluation of our manuscript and for the constructive and insightful comments provided. We have carefully considered all suggestions and have revised the manuscript accordingly to improve its clarity, methodological transparency, and overall robustness, as follows.

RESPONSES TO EDITOR

1. Please ensure that your manuscript meets PLOS ONE's style requirements, including those for file naming.

RESPONSE: The manuscript has been revised accordingly based on PLOS ONE’s style requirement.

2. Thank you for stating the following financial disclosure: “This work was supported by the National Institute of Occupational Safety and Health (NIOSH) Malaysia [grant number: UKMP-S230424] to [HMY]. Please state what role the funders took in the study. If the funders had no role, please state: “The funders had no role in study design, data collection and analysis, decision to publish, or preparation of the manuscript”. If this statement is not correct you must amend it as needed. Please include this amended Role of Funder statement in your cover letter; we will change the online submission form on your behalf.

RESPONSE: Thank you for the reminded. Indeed, the funder (NIOSH Malaysia) had no role in study design, data collection and analysis, decision to publish, or preparation of the manuscript. This statement has been added to the manuscript and the cover letter.

3. We note that you have indicated that there are restrictions to data sharing for this study. For studies involving human research participant data or other sensitive data, we encourage authors to share de-identified or anonymized data. However, when data cannot be publicly shared for ethical reasons, we allow authors to make their data sets available upon request. For information on unacceptable data access restrictions, please see http://journals.plos.org/plosone/s/data-availability#loc-unacceptable-data-access-restrictions. Before we proceed with your manuscript, please address the following prompts:

• If there are ethical or legal restrictions on sharing a de-identified data set, please explain them in detail (e.g., data contain potentially identifying or sensitive patient information, data are owned by a third-party organization, etc.) and who has imposed them (e.g., a Research Ethics Committee or Institutional Review Board, etc.). Please also provide contact information for a data access committee, ethics committee, or other institutional body to which data requests may be sent.

• If there are no restrictions, please upload the minimal anonymized data set necessary to replicate your study findings to a stable, public repository and provide us with the relevant URLs, DOIs, or accession numbers. Please see http://www.bmj.com/content/340/bmj.c181.long for guidelines on how to de-identify and prepare clinical data for publication. For a list of recommended repositories, please see https://journals.plos.org/plosone/s/recommended-repositories. You also have the option of uploading the data as Supporting Information files, but we would recommend depositing data directly to a data repository if possible.

RESPONSE: Regrettably, the Long COVID dataset cannot be shared publicly primarily due to concerns around patient privacy, legal regulations, and ethical approvals. Even when personal identifiers are removed, individuals may still be re-identified from combined data (e.g., working department, position at company, work schedule, etc), especially in workplace with small number of workers. Additionally, the Personal Data Protection Act 2010 restricts the sharing of sensitive health information. Furthermore, the ethics committee limits data use to research purposes, and public sharing may violate the conditions under which participants originally consented. This explanation is provided at the availability of data and materials section.

4. Please include captions for your Supporting Information files at the end of your manuscript, and update any in-text citations to match accordingly. Please see our Supporting Information guidelines for more information: http://journals.plos.org/plosone/s/supporting-information.

RESPONSE: The captions of all supporting information files has been added to the end of the manuscript, and the in-text citations have been revised accordingly.

RESPONSES TO REVIEWER 1

1. Introduction (abstract): The two sentences seem to contradict each other. The first statement mentions a high prevalence of Long COVID, while the second indicates that the study’s goal was to determine the prevalence. Consider rephrasing. Consider highlighting the knowledge gap and problem statement in this section.

RESPONSE: Thank you for highlighting the blind spot. To better illustrate the research gap and the justifications of the present study, the introduction of the abstract has been revised to: “The high prevalence and multiorgan involvement of Long COVID among the general population has raised concerns about the possible long-term effect of this condition among industrial workers”. It was the observation of the high prevalence of Long COVID among the general population that inspired the authors to conduct a study specifically among the manufacturing workers.

2. Materials and methods (abstract): The instruments used are mentioned but the methods used to distribute the instruments are not. Please state accordingly. Consider moving the total number of respondents (i.e., 797) to the results section. Consider adding the inclusion and exclusion criteria of participants. Line 52 – to correct the spelling of the word ‘utilised’.

RESPONSE: The methods of distributing the questionnaires was added to the materials and methods section of the abstract. Due to the word limit of the abstract, we could only add a brief description of the inclusion criteria of the participants. The total number of participants has been moved to the result section as advised and spelling error has been corrected.

3. Results (abstract): For better clarity, consider restructuring the first part of the results section. I would suggest starting with the number of respondents, followed by prevalence and so on. Line 59-60: There is a repetition of information i.e., 42.4%. Consider rephrasing. Regarding the reported risk factors, consider reporting key effect sizes (e.g., adjusted odds ratios) with 95% confidence intervals to allow readers to interpret the magnitude of the associations.

RESPONSE: The number of participants has been added. The repetition of 42.4% was removed. The aOR for all associated risk factors were included.

4. Conclusion (abstract): The conclusion mostly repeats the results and is quite general. Consider making it stronger by emphasizing the main implications of the findings for occupational health practices or workplace policies that support workers dealing with Long COVID. This would give readers a clearer idea of the importance of the study.

RESPONSE: Due to the word limits in abstract, we are unable to extensively elaborate on the impact of the study. However, we concisely emphasised the study implications as follows: “The impact of Long COVID on WRQoL, sickness presenteeism and sickness absenteeism, as well as the multifactorial nature of adverse work outcomes highlights the need for targeted workplace interventions, supportive policies, early detection, and inclusive occupational health frameworks at the workplace”.

5. Study design: I would recommend using the phrase ‘cross-sectional survey’ instead.

RESPONSE: The authors agree that the phrase “cross-sectional survey” is a better description than “cross-sectional study”. Hence, it has been revised accordingly.

6. Data collection protocol: The protocol was described but could be further elaborated. Consider including this information for better clarity:

• Any method used to select the manufacturing companies

• The individual that the researcher reached out to provide the list.

• Were similar data collection protocols used at all companies visited.

• The number of researchers present at the site (if relevant).

• The kind of assistance provided during the data collection e.g., explanation on medical terms such as tachycardia, tinnitus etc.

Consider incorporating the specific types of demographic data gathered in this section. Furthermore, it would be beneficial to indicate the methods used for data collection, such as whether body weight and height were self-reported or measured.

RESPONSE: Initially, manufacturing companies listed in the Federation of Malaysian Manufacturers (FMM) directory that met the eligibility criteria were selected using a multistage sampling approach. However, due to a very low response rate (e.g., companies declining participation), the researchers subsequently adopted a convenience sampling method. Under this approach, any company that provided consent to participate from each zone in Malaysia was included. After obtaining informed consent from the company, the human resource personnel were contacted to provide a list of workers (i.e., the sampling frame). A standardised data collection protocol was applied across all participating companies. During data collection, a team of 2–5 researchers/enumerators (previously trained using the standardised protocol) was present at each site to conduct researcher-assisted interviews while administering the questionnaire, including clarifying items and explaining complex medical terminology. Sociodemographic information, including age, gender, BMI (from self-reported weight and height), and smoking status, was collected.

7. Ethical approval: It is unclear why the study requires MREC approval. To clarify. Consider indicating if any authorisation was provided from the manufacturing companies to visit and conduct data collection.

RESPONSE: The study applied for MREC ethics approval as some of the participants were healthcare professionals working at the Ministry of Health, Malaysia. Written/electronic consent was also obtained from the respective manufacturing companies prior to study collection. Such clarification has been included to the materials and methods section.

RESPONSES TO REVIEWER 2

1. Scope of study: The study implies nationwide coverage (e.g. “entire nation”, “across all states”) but there’s lack in clarity regarding geographical precision; the distribution and number of participating manufacturing companies are not explicitly reported. The sampling strategy and data collection procedures are not sufficiently detailed giving an impression of vague representation.

RESPONSE: A cross-sectional survey was conducted across Malaysia’s five geographical zones (i.e., East Coast, Northern, Southern, Central, and East Malaysia). Initially, a multistage sampling strategy was planned for the selection of manufacturing companies, involving cluster sampling within each geographical zone based on the total number of companies, followed by proportionate stratified sampling to select workers from the states within each zone. However, due to a very low response rate (e.g., companies declining participation), the researchers opted to use a convenience sampling method instead. This resulted in three, four, three, six, and two companies participated across East Coast, Northern, Southern, Central, and East Malaysia, respectively. Under this approach, any company that provided consent to participate from each zone in Malaysia was included. This was followed by a universal sampling to select workers from each factory.

2. Enrolment criteria: The inclusion and exclusion criteria set may not be sufficiently comprehensive and leaving gaps in ruling out confounding symptoms or pre-existing conditions that could overlap with or mimic Long COVID. More explicit criteria are necessary to strengthen internal validity and reduce misclassification bias.

RESPONSE: We acknowledge that the initial inclusion and exclusion criteria may not have fully accounted for potential confounding symptoms or pre-existing conditions that could overlap with or mimic Long COVID, such as bronchial asthma and chronic obstructive pulmonary disease. To address this, several measures were incorporated into the study design and data collection process. Firstly, information on pre-existing comorbidities (e.g., respiratory, cardiovascular, and metabolic conditions) was collected and participants included in the multivariate analysis to allow adjustment for potential confounders (as reported in appendix). Secondly, participants were required to have a confirmed diagnosis of COVID-19 within a defined time frame (i.e., fully fulling the definition of WHO), to ensure temporal relevance to Long COVID assessment. Thirdly, symptoms were assessed in relation to their onset following COVID-19 infection, which helped differentiate new or persistent symptoms from pre-existing conditions. Such discussion has been added to the discussion section.

3. Survey instruments: The use of established, validated instruments to assess outcomes is a strength and contributes to methodological rigour. However, the development and application of the Long COVID instrument are not sufficiently explained. Reporting of “prevalence” of symptoms and adverse outcomes only accounted for workers alleged LC symptoms. Reporting of subjective parameters remains vague, raising analytical and interpretative concerns regarding measurement validity and comparability.

RESPONSE: Our apology for the blind spot. The development and validation process of the Long COVID questionnaire has been reported in our previously published study protocol, available at: Yusoff HM, Mohamed NC, Yew SQ, Nawi AM, Rashidah Ismail OH, Tohit NM, et al. Prevalence and risk factors of long covid and its associated adverse work outcomes among workers in the manufacturing sector in Malaysia – a mixed-methods study protocol. J Health Popul Nutr. 2024 Nov 25;43(1):194. doi:10.1186/s41043-024-00696-z. Meanwhile, the psychometric properties of the Long COVID questionnaire is still in press. This clarification has been added to the materials and method section.

4. Study design limitations and interpretation of findings: The limitations inherent to the cross-sectional design are not adequately addressed. Greater transparency is needed regarding the structure and content of the survey tools, particularly in relation to several paradoxical or counterintuitive findings (e.g. high WRQoL despite symptom burden), which warrant further methodological and analytical clarification.

RESPONSE: To enhance transparency regarding the structure and content of the survey tools, the development and validation process of the Long COVID questionnaire is described in the study protocol (reference added to the manuscript). However, the questionnaire cannot be made publicly available as it is copyrighted by the sponsor (NIOSH Malaysia). With regard to the seemingly paradoxical finding of relatively high WRQoL despite the presence of Long COVID symptoms, several explanations are possible. Firstly, sufficient coping strategies among workers may mitigate the perceived impact of persistent symptoms on WRQoL. For instance, work-focused rehabilitation, multidisciplinary inpatient and outpatient rehabilitation, psychoeducation, pacing, and breathing strategies, have shown to shift focus from symptom monitoring to optimising functional outcomes. These strategies allowed workers affected by Long COVID to maintain good WRQoL. Secondly, many participants may have experienced mild or manageable symptoms (as most participants only experience one symptom) that did not substantially impair their work functioning, as observed in the low proportion of workers with sickness absenteeism, sickness presenteeism, and safety issues at work. These points have now been discussed in the revised discussion section to provide clearer interpretation of the findings.

5. Conceptual framework and conclusions: The scope of the study and its underlying conceptual framework are not clearly articulated. Several claims and conclusions may require revision or qualification in view of the study design and sampling approach.

RESPONSE: The study is based on the premise that Long COVID workers may experience adverse work outcomes, which is associated with socio

Attachment Submitted filename: Responses to Reviewers (Revision 2).pdf

# Editor report: Decision Letter 2

**DOI:** 10.1371/journal.pone.0357125.r005

**Authors:** Shairyzah Ahmad Hisham

13 Jul 2026

Dear Dr. Yew,

Please refer to the comments provided in the attached reviewed manuscript and address the required revisions to further strengthen the scientific rigor and overall quality of the publication.

Please submit your revised manuscript by Aug 27 2026 11:59PM. If you will need more time than this to complete your revisions, please reply to this message or contact the journal office at plosone@plos.org. When you're ready to submit your revision, log on to https://www.editorialmanager.com/pone/ and select the 'Submissions Needing Revision' folder to locate your manuscript file.

- A letter that responds to each point raised by the academic editor and reviewer(s). You should upload this letter as a separate file labeled 'Response to Reviewers'.
- A marked-up copy of your manuscript that highlights changes made to the original version. You should upload this as a separate file labeled 'Revised Manuscript with Track Changes'.
- An unmarked version of your revised paper without tracked changes. You should upload this as a separate file labeled 'Manuscript'.

If you would like to make changes to your financial disclosure, please include your updated statement in your cover letter. Guidelines for resubmitting your figure files are available below the reviewer comments at the end of this letter.

If applicable, we recommend that you deposit your laboratory protocols in protocols.io to enhance the reproducibility of your results. Protocols.io assigns your protocol its own identifier (DOI) so that it can be cited independently in the future. For instructions see: https://journals.plos.org/plosone/s/submission-guidelines#loc-laboratory-protocols. Additionally, PLOS ONE offers an option for publishing peer-reviewed Lab Protocol articles, which describe protocols hosted on protocols.io. Read more information on sharing protocols at https://plos.org/protocols?utm_medium=editorial-email&utm_source=authorletters&utm_campaign=protocols.

As the corresponding author, your ORCID iD is verified in the submission system and will appear in the published article. PLOS supports the use of ORCID, and we encourage all coauthors to register for an ORCID iD and use it as well. Please encourage your coauthors to verify their ORCID iD within the submission system before final acceptance, as unverified ORCID iDs will not appear in the published article. *Only*  the individual author can complete the verification step; PLOS staff *cannot*  verify ORCID iDs on behalf of authors.

We look forward to receiving your revised manuscript.

Kind regards,

Shairyzah Ahmad Hisham, PhD.

Academic Editor

PLOS One

Journal Requirements:

1. If the reviewer comments include a recommendation to cite specific previously published works, please review and evaluate these publications to determine whether they are relevant and should be cited. There is no requirement to cite these works unless the editor has indicated otherwise.

2. Please review your reference list to ensure that it is complete and correct. If you have cited papers that have been retracted, please include the rationale for doing so in the manuscript text, or remove these references and replace them with relevant current references. Any changes to the reference list should be mentioned in the rebuttal letter that accompanies your revised manuscript. If you need to cite a retracted article, indicate the article’s retracted status in the References list and also include a citation and full reference for the retraction notice.

To ensure your figures meet our technical requirements, please review our figure guidelines: https://journals.plos.org/plosone/s/figures

You may also use PLOS’s free figure tool, NAAS, to help you prepare publication quality figures: https://journals.plos.org/plosone/s/figures#loc-tools-for-figure-preparation.

NAAS will assess whether your figures meet our technical requirements by comparing each figure against our figure specifications.

Attachment Submitted filename: PONE-D-25-68765_R2 - reviewed.pdf

# Author comment: Author response to Decision Letter 3

**DOI:** 10.1371/journal.pone.0357125.r006

14 Jul 2026

We sincerely thank the reviewer for this insightful comment, which has helped improve the clarity of the manuscript.

1. Not sure why this is here since all subjects have long COVID. Is it the symptoms?

RESPONSE: Thank you for highlighting this issue. Indeed, all participants had Long COVID. To avoid confusion, the phrase has been revised to “Long COVID symptoms” to clarify that the risk factors examined included both the number of Long COVID symptoms and the presence of specific symptoms.

Other grammatical errors and problematic phrases have been revised accordingly.

Attachment Submitted filename: Response to Reviewer (R3).docx

# Editor report: Decision Letter 3

**DOI:** 10.1371/journal.pone.0357125.r007

**Authors:** Shairyzah Ahmad Hisham

12 Aug 2026

Prevalence and risk factors of adverse work outcomes among manufacturing workers with Long COVID in Malaysia – a nationwide study

PONE-D-25-68765R3

Dear Dr. Yew,

We’re pleased to inform you that your manuscript has been judged scientifically suitable for publication and will be formally accepted for publication once it meets all outstanding technical requirements.

Within one week, you’ll receive an e-mail detailing the required amendments. When these have been addressed, you’ll receive a formal acceptance letter and your manuscript will be scheduled for publication.

An invoice will be generated when your article is formally accepted. Please note, if your institution has a publishing partnership with PLOS and your article meets the relevant criteria, all or part of your publication costs will be covered. Please make sure your user information is up-to-date by logging into Editorial Manager at Editorial Manager® and clicking the ‘Update My Information' link at the top of the page. For questions related to billing, please contact billing support.

If your institution or institutions have a press office, please notify them about your upcoming paper to help maximize its impact. If they’ll be preparing press materials, please inform our press team as soon as possible -- no later than 48 hours after receiving the formal acceptance. Your manuscript will remain under strict press embargo until 2 pm Eastern Time on the date of publication. For more information, please contact onepress@plos.org.

Kind regards,

Shairyzah Ahmad Hisham, PhD.

Academic Editor

PLOS One

Additional Editor Comments (optional):

The revisions made in response to the reviewers’ and Academic Editor’s comments have substantially strengthened the manuscript and improved its scientific rigor. The authors have addressed the concerns satisfactorily.

# Editor report: Acceptance letter

**DOI:** 10.1371/journal.pone.0357125.r008

**Authors:** Shairyzah Ahmad Hisham

PONE-D-25-68765R3

PLOS One

Dear Dr. Yew,

I'm pleased to inform you that your manuscript has been deemed suitable for publication in PLOS One. Congratulations! Your manuscript is now being handed over to our production team.

At this stage, our production department will prepare your paper for publication. This includes ensuring the following:

* All references, tables, and figures are properly cited

* All relevant supporting information is included in the manuscript submission,

* There are no issues that prevent the paper from being properly typeset

You will receive further instructions from the production team, including instructions on how to review your proof when it is ready. Please keep in mind that we are working through a large volume of accepted articles, so please give us a few days to review your paper and let you know the next and final steps.

Lastly, if your institution or institutions have a press office, please let them know about your upcoming paper now to help maximize its impact. If they'll be preparing press materials, please inform our press team within the next 48 hours. Your manuscript will remain under strict press embargo until 2 pm Eastern Time on the date of publication. For more information, please contact onepress@plos.org.

You will receive an invoice from PLOS for your publication fee after your manuscript has reached the completed accept phase. If you receive an email requesting payment before acceptance or for any other service, this may be a phishing scheme. Learn how to identify phishing emails and protect your accounts at https://explore.plos.org/phishing.

If we can help with anything else, please email us at customercare@plos.org.

Thank you for submitting your work to PLOS One and supporting open access.

Kind regards,

PLOS One Editorial Office Staff

on behalf of

Dr. Shairyzah Ahmad Hisham

Academic Editor

PLOS One
